# Cannabinoid impacts on ethnic modulation of atrial septal defect prevalence USA

**DOI:** 10.1038/s41372-026-02717-7

**Published:** 2026-05-27

**Authors:** Albert Stuart Reece, Gary Kenneth Hulse

**Affiliations:** 1 Division of Psychiatry, University of Western Australia, Crawley, WA Australia; 2 School of Medical and Health Sciences, Edith Cowan University, Joondalup, WA Australia

**Keywords:** Pathogenesis, Epidemiology

## Abstract

**Objective:**

To conduct a detailed epidemiological exploration of the relative contributions of cannabis and ethnicity to US atrial septal defect (ASD) rates (ASDR).

**Study design:**

State-based ASDR data from the US National Births Defects Prevention Network, substance use, income and ethnicity data analyzed in RStudio.

**Results:**

Ethnic effects were significant with relative risks amongst African Americans and American Indians and Alaskan Natives of 2·40 (95%C.I. 2·27, 2·54) and 2·31 (2·19, 21·43), Cohen’s D of 1·44 and 1·46 and *P* values of 2·94 × 10^−168^ and 3·01 × 10^−172^ compared to others, respectively. In general, additive models inclusion of ethnicity:cannabinoid and ethnicity:tobacco interactions were significant down to P=zero for cannabis, Δ9THC and cannabidiol. Sequentially doubly robust targeted multiple likelihood estimations confirmed epidemiologically causal relationships under standard assumptions. ASDR amongst Asians and Pacific Islanders in Nevada showed an exponential doubling time of 2.83 years.

**Conclusions:**

Cannabinoid and cannabinoid:ethnicity interactions drive ASDR and meet epidemiological causal criteria.

## Introduction

Whilst official statements from Centers for Disease Control, Atlanta, Georgia (CDC) acknowledge the prevalence of minor congenital cardiac anomalies has increased in recent years, the officially cited atrial septal defect (ASD) rate (ASDR) is only 10.3/10,000 live births based upon data from Atlanta, Georgia 1998–2005 [1] whereas published figures include 51 rates above 300/10,000, including peak rates amongst various ethnicities in Nevada and Mississippi of 884, 849 and 802/10,000 [2].

Official rates published by the CDC-affiliated National Birth Defects Prevention Network (NBDPN) reveal the presence of clear and obvious variations by ethnic background [2]. Whilst some of this variation may arise from an unstable denominators inherent in the smaller minority populations, this does not explain the broadly acting secular increase across time, the widespread discrepancies in ethnic groups, nor the consistency of ASDR elevation confined to certain ethnic populations.

Several recent large population based studies have identified that cannabis is causally related to ASDR, including work conducted in Canada, Australia, Colorado, Hawaii and Europe and an earlier study from USA [3–10]. Cardiovascular complications of cannabinoid exposure are well described [6, 11–15] but less widely recognized than psychoneurological complications. Morphogenesis of the heart and great vessels occurs by a complicated, delicately choreographed process involving the migration of cells from the primary, secondary and lateral heart fields, proepicardium, nuchal crest and pharyngeal arches all carefully controlled by sequential activation of cardiogenic gene cassettes under epigenomic control and the influence of many local morphogen gradients [6, 16]. Cannabinoids are well described to disrupt both multiple morphogen gradients [17–23] and the DNA methylation [6, 9, 24–28] state of multiple cardiogenic genes [24] so that disrupted morphogenesis of the central cardiovasculature is predictable and mechanistically consistent with cannabinoid-induced teratogenicity [3, 6, 9, 14].

Cannabinoid teratogenicity is itself a subset of a wider group of conditions in the family of cannabinoid-related genotoxicity, which includes cannabinoid-induced carcinogenesis, mental impairments and aging. Since early termination of pregnancy for anomaly (ETOPFA) is not practised for ASD, ASDR becomes a key bellwether marker for cannabinoid-related teratogenesis more generally and indeed for the wider group of cannabinoid-related genotoxic disorders [28].

The question naturally arises as to how these two apparent risk factors compare and in particular the issue of whether differing patterns of cannabis use or exposure in ethnic minorities accounts for their apparent increased predisposition to ASD. Pre-specified hypotheses tested by this study were: (1) to examine recent ethnic trends in ASDR particularly for consistency of effect; (2) to examine the extent to which differing patterns of cannabis consumption explained differing ethnic prevalences; (3) to examine the association of particular cannabinoids with ASDR and (4) to conduct statistical modelling on the highest rate of all which was documented amongst the Non-Hispanic Asian Pacific Islander population of Nevada.

## Methods

### Data

Ethnic specific data on ASDR were manually extracted from 12 Periodical Reports of the National Birth Defects Prevention Network (NBDPN) [2], 1989–1990 to 2016–2020, which is affiliated with the Centers for Disease Control (CDC), Atlanta, Georgia. Listed ethnicities included Non-Hispanic White (NHWhite), Non-Hispanic Black (NHBlack), Hispanic, Non-Hispanic Asian/Pacific Islander (NHAsPI), Non-Hispanic American Indian Alaskan Native (NHAIAN) and Total (Overall). NHAsPI was taken as the comparator ASDR. NBDPN reports refer to 5-year periods from which the middle year was used as the indicative year (e.g. for 2008–2012, 2010). State level drug use was from the Restricted Data Analysis Series (RDAS) of National Survey of Drug Use and Health (NSDUH) conducted annually by Substance Abuse and Mental Health Services Administration (SAMHSA) [29]. Data for last month alcohol use (alcmon), last month binge alcohol (bngalc), alcohol dependence (abodalc), last month cigarette (cigmon), last month cannabis (mrjmon), last year analgesic misuse (anlyr) and last year cocaine use (cocyr) were used. Federal level ethnic specific drug use was accessed from NSDUH. Median household income and state ethnic populations were taken from US Census. Cannabis legal status was identified from online sources. Drug Enforcement Agency (DEA) seizure data provided national level cannabinoid data. CDC WONDER provided birth data. Methods of case ascertainment by state were obtained from the CDC quadrennial reports [2]. Legal status was determined from published sources [30, 31]

### Derived data

The state-specific numbers of people of each ethnicity using drugs were compared to the overall prevalence of the ethnic population of that state to derive a relative ethnic rate of drug use in that state (e.g. mrjRel). This was then multiplied by the level of that drug use nationally (e.g. mrjmon) to estimate ethnic cannabis exposure (mrjRelmrj). This was then multiplied by the THC content at the national level to estimate an average Δ9THC exposure for that ethnicity (mrjRelmrj9THC). The product of the federal level cannabinoid concentrations and cannabis use rate in that state was used as state-specific estimates of cannabinoid exposure. Ethnicity was dichotomized into NHBlack and NHAIAN (NHAA_AIAN) v. remainder (Others).

### Statistics

Data was processed in RStudio 2025.05.0 as GUI for R 4.5.2. Data was log transformed in the interests of normality assumptions. Covariates thus transformed included the ASD rate (ASDRt), cannabis, cocaine and analgesic use and median income. Data was manipulated using dplyr and graphs drawn using ggplot, both from tidyverse [32]. Plots were arranged using ggpubr [33]. Package ipw was used to inverse probability weight all regression models except where indicated [34]. nlme, lmerTest, lme4 were used for mixed effects models with State as the random covariate [35–37]. Models were compared with ANOVA tests and the Akaike Information Criterion (AIC). E-Values were calculated using the package Evalue [38, 39]. The classical technique of model reduction was used involving deletion of the least significant term. Effect Sizes were calculated with z- and arcsinh- transformed covariates (to make all data ranges comparable) using packages performance, effectsize and emmeans [40–42]. Model extrapolation was conducted with the predict function from R base on polynomial, exponential and supra-exponential functions of the form y ~ x^z^w^. Panel regression was conducted with package plm [43]. Generalized additive modelling (GAM) was conducted in mgcv [44–46] using the negative binomial distribution (appropriate for rare events) from MASS with log (births) as the offset [47]. Tensor product interactions (te) were used where appropriate. Covariates for GAM’s were both z- and arcsinh- transformed to produce similar covariate ranges and normalize distributions. Directed diagrams were drawn with DiagrammeR and DiagrammeRsvg [48, 49]. *P* < 0.05 was considered significant.

### Correlated random effects (Mundlak decomposition)

To distinguish within-state from between-state exposure effects, we decomposed cannabis exposure and income into state-specific means and within-state deviations. Models included both components simultaneously to assess whether associations were driven by persistent between-state differences or temporal changes within states.

### Doubly robust targeted estimation

Population intervention effects were estimated using the sequentially doubly robust (SDR) estimator implemented in the Longitudinal Modified Treatment Policies (LMTP; “lmtp”) and SuperLearner T packages [50–52]. The intervention consisted of a stochastic shift corresponding to a doubling of cannabis exposure (log-scale shift of +log(2)). Baseline covariates included ethnicity, income, ascertainment methodology, legal status, and year.

Super Learner ensembles combining generalized linear and penalized regression models were used for both exposure and outcome regressions. Variance estimation was based on the efficient influence function with clustering at the state level. Effect estimates are reported as exponentiated contrasts on the log scale and interpreted as multiplicative changes in ASD rates under the specified exposure shift.

#### Causal inference/SDR–TMLE population intervention

##### Estimand and intervention

Population intervention estimand (doubling exposure). Let *Y* denote the log ASD rate, *A* denote log cannabis exposure, and *W* denote measured covariates (ethnicity, income, ascertainment method, legal status, year). We estimated the expected outcome under a modified treatment policy that shifts cannabis exposure upward by log(2)(doubling on the original scale), ${A}^* =A+\log (2)$, with truncation to the observed support when needed. The target estimand was the population mean difference $E[Y({A}^* )]-E[Y(A)]$, and we exponentiated estimates to report multiplicative effects and percent changes on the original ASD rate scale. It should be emphasized that causal inference occurs at the level of populations only, rather than individual participant level.

##### Doubly robust estimation via SDR–TMLE

We used sequentially doubly robust targeted maximum likelihood estimation (SDR–TMLE) as implemented in the lmtp package. SDR–TMLE combines outcome regression and treatment modeling such that the estimand is consistent if either the outcome model or the treatment model is correctly specified (under standard identification assumptions). Nuisance functions were estimated using an ensemble of generalized linear models and penalized regression (e.g., SL.glm, SL.glmnet) with K-fold cross-fitting to reduce overfitting bias.

#### Covariate adjustment and clustering

##### Adjustment set and dependence

The covariate set *W* included ethnicity (Race), income (log MHY), case ascertainment method (Asctt), legal status (Status), and calendar year. Because observations were clustered within states over time, uncertainty was estimated with state-level clustering. Specifically, we formed differences of efficient influence functions between shifted and baseline policies and computed standard errors from state-level means, yielding cluster-robust confidence intervals on the log scale; results were then transformed to rate ratios and percent changes.

### Literature search

The databases PubMed, Medline, Scopus, Embase, Web of Science, Toxline, Mendeley, Current Contents, Biomed Central, Elsevier, and Springer were searched for the terms “cannabis”, “marijuana” and “atrial septal defect” (ASD) across all languages. Seven studies were identified providing moderate to high quality data often fulfilling causal criteria [3–9]. Ethnicity was found to be a clear risk factor for ASD in all of the published reports from National Birth Defects Prevention Centre 1989–1990 top 2016–2020 [2].

## Results

2521 ASDRs were extracted from NBDPN reports. Of these, 1882 were non-zero and fell in the period 2005–2018, which overlapped the NSDUH substance exposure dataset. Data represented 406,893 ASDs and 65,618,252 live births, including Hispanics.

Baseline cohort data are shown in eTable 1 dichotomized by ethnicity. Groups are largely comparable but show different substance exposure and ASDRs.

eTable 2 shows the state ASD Rates by ethnicity which range from 884·0, 849·6, 802·0, 793·1 and 772·8 for ethnicities in Nevada and Mississippi, down to 0·5 and 0·4 /10,000 livebirths in Maryland and Nebraska. eFigures 1 and 2 present state-specific ASDR using different scales for each panel.

Time-aggregated ethnic-specific ASDRs are presented (eTable 3, eFig. 3). eFigure 3A focusses on the spread of the upper outliers. eFigure 3B shows that the notches do not overlap indicating statistically significant differences. eFigure 3C shows marked lack of notch overlap in extreme groups on a log plot. eFigure 4 presents this data as a bar graph with confidence intervals.

**Fig. 1:**
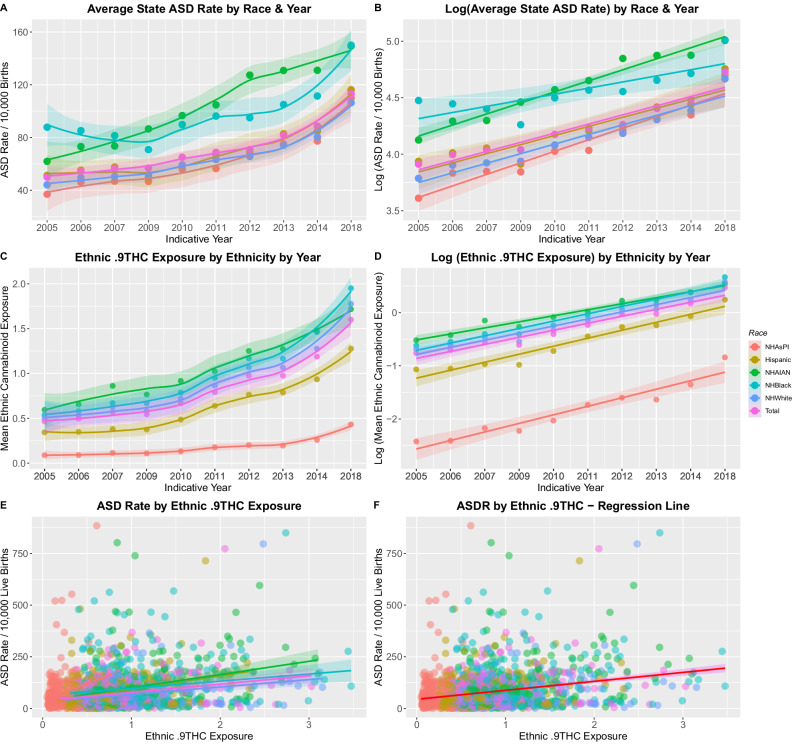
Metrics of ASD Rates. **A** Average State ASD rate by race and year; **B** Log (average State ASD rate) by race and year; **C** Ethnic Δ9THC exposure rate by race and year; **D** Log (ethnic Δ9THC exposure) rate by race and year; **E** ASD Rate by ethnic Δ9THC with separate regression line for each ethnicity and **F** ASD Rate by ethnic Δ9THC with common regression lines for all ethnicities.

ASDRs are shown over time in Fig. 1(A) and (B) as linear and log plots. Figure 1(C) and (D) show ethnic Δ9THC exposure over time as linear and log plots. It is noted that linear progression on a log plot indicates exponential expansion. Mixed effects analysis of these ethnic effects on ASDRs indicates high levels of significance (to 3·01x10^-172^), moderately strong E-values to 4·24 (95% Lower C.I. 3·97), R.R. 2·42 (95%C.I. 2·27, 2·54) and Cohen’s D greatly elevated to 1·44 (1·34, 1·54) and 1·46 (1·36, 1·56), changes most marked in the NHAIAN and NHBlack groups (eTable 3). For these reasons, these two ethnicities were paired and opposed to the other groups and ethnicity was dichotomized on this basis.

Figure 1E and F show the ASDR by ethnic Δ9-tetrahydrocannabinol (Δ9THC) exposure with separate and common regression lines, respectively. Panel E shows that these lines mostly overlap. The regression line shown in Fig. 1F is significant (β-est. = 0·2644, *P* = 3·99 × 10^-17^; model Adj.R.Squ.=0·036, S.D. = 1·07, AIC = 5603, *P* = 3·99 × 10^-17^).

eTable 4 considers the effect of ethnicity on ASDR in mixed models and provides E-values, relative risks and Cohen’s D as a measure of effect size. eTable 5 considers ethnicity as both a dichotomized and as a six-level factor. Considered as a dichotomous factor both cannabis and ethnicity are highly significant and initially have an elevated Cohen’s D at 0·59. However, this collapses to 0·0096 with addition of ethnic cannabis as an additive term and then as an interaction. A similar pattern is observed for the six-level ethnicity factor where Cohen’s D drops from 0·78 to 0·01. When the method of case ascertainment and cannabis legal status are added in to mixed effects modelling, both these covariates are not significant and ethnic cannabis is the most highly significant variable (see ANOVA table; eTable 6).

A series of hybrid correlated random effects (Mundlak) mixed effects models was developed, where within- and between- state ethnic cigarette and cannabis exposure were used to model the effect of variations within state compared to those between them (eTable 7). Tobacco alone accounted for 3% of the variance compared with 8% for cannabis. An additive model including all substances, income and ethnicity accounted for 15% of the variance with a total of 80.8% of the conditional variance accounted for mostly by model structure. Terms incorporating ethnicity were significant at much lower level. Models were compared formally and show rising AIC and marginal variance (eTable 8 and eFig. 5).

eFigure 6A shows log (ASDR) by dichotomized ethnicity. Clear differences emerge (eTable 9). eFigure 6B models ASDR by ethnic THC exposure as a single group with a loess fit. Panels C and D present these by dichotomized ethnicity. Mixed effects models of dichotomized ethnicity and ethnic cannabis are shown (eTable 9).

eTable 10 presents additive and interactive models of ethnic cannabinoid exposure for Δ9THC and cannabidiol. In each case both cannabinoid and ethnicity terms remain significant. The third and fourth models were also run with and without terms for ethnic Δ9THC. The third model including ethnic Δ9THC (AIC = 3080·63) was better than without (AIC = 3110·52; Anova Log.Ratio=33·75, *P* = 6·29 × 10^−9^). The same was true for the fourth model (AIC = 3034·59 v. 3063·82, Anova Log.Ratio = 46·73, *P* = 1·07 × 10^−5^).

Estimated marginal means, trends and effect sizes are shown for ethnicity (eTable 11) and ethnic contrasts (eTable 12) for both Δ9THC and cannabidiol. Effect sizes are shown from the final interactive ethnic Δ9THC model in eTable 10 in eFig. 7 and predictions from this model appear in eFig. 8. Both the form and the scale of these predicted values are markedly similar to those shown in eFig. 6B confirming their accuracy.

As well as considering the ASDR’s themselves it is also of interest to consider the slopes of the cannabis-ASDR regression lines which are known as “elasticities”. These are shown for Δ9THC and cannabidiol in eTable 11 and various ethnic contrasts appear in eTable 12. The elasticities for ethnic cannabis are shown in eTable 13 and the predicted effects on those elasticities of raising cannabis exposure by 50% and 100% appear in eTable 14. In the sample considered overall the effect of doubling cannabis was to raise the ASR elasticity 30.58% (95%C.I. −2.14%, 74.23%; ΔLog 0.27 (−0.02, 0.56), R.R. 1.31 (0.98, 1.74)). Significant effect modification by ethnicity was observed. When panel regression was performed terms including ethnic cannabis and were significant (eTable 15). A Mundlak correlated random effects panel model was also performed (last model shown). In that model 84% of the variance of ASDR was accounted for by between-state effects and 16% was attributable to within-state variation.

**Fig. 2:**
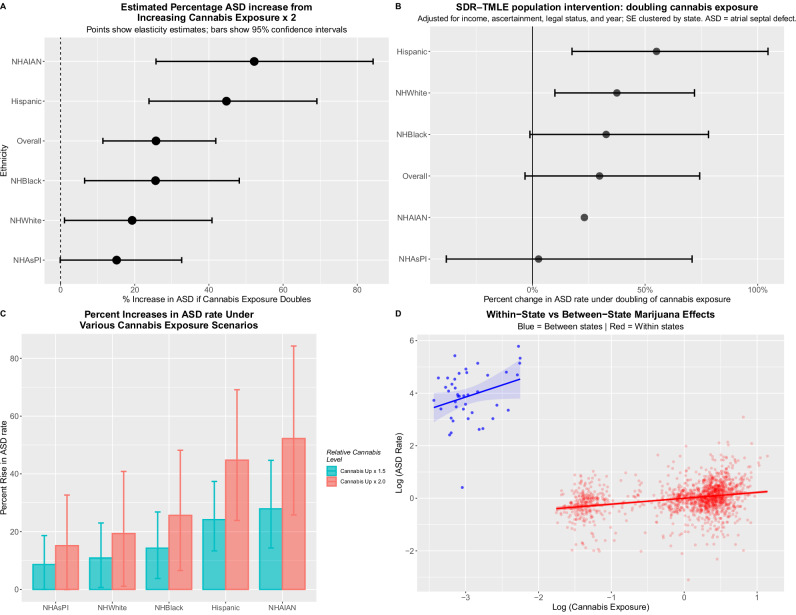
Various ASD-Cannabis Regression Line Slopes. **A** Slopes of ASDR-cannabis regression lines by ethnicity from mixed effects regression models; **B** slopes of ASD-cannabis regression lines under a sequentially doubly robust (SDR) targeted maximum likelihood estimation (TMLE) analytical framework; **C** percentage increase of ASD-cannabis slopes from mixed effects regression models and **D** Between-states variation compared to within states variation from a Mundlak mixed effects regression model.

Some of these changes are summarized in Fig. 2. Panel A shows the effect of doubling cannabis exposure on ASDR from mixed effects models. Panel B shows the effects of doubling cannabis on the elasticities of the ASDR regression lines under a sequentially doubly robust targeted maximum likelihood estimation (SDR-TMLE) analysis. Panel C presents the effects of increasing cannabis exposure by 50% and 100% as bar graphs. Panel D shows that between-state effects account for much more of the ASDR variance than within-state effects (from eTable 15).

eFigure 9 and eTable 16 presents the results of a Difference-in-Difference analysis where the cannabis legal paradigm changed with the reference year taken as the year of change. Most changes are not significant.

Mean cannabis use across the analysis period rose from 5.8% to 11.1% from 2005 to 2018. It was therefore of interest to consider the effects of doubling cannabis exposure. A powerful analytical framework in which to consider this is the longitudinal modified treatment policies (LMTP) framework using the R package lmtp [50, 52]. Most races had data from 42 states; for the NHAIAN race data from 39 states was available. Covariates included ethnic cannabis exposure, income, case ascertainment, legal status, state and year. The impacts on ASDR-ethnic cannabis elasticities are shown in eTable 17 for the change in log slope, change in relative rate and percent change.

A sensitivity analysis was then conducted on these results (eTable 18). Model A was the main SDR-TMLE analysis. Model B included only those states where the legal status of cannabis changed. Model C excluded states where the within-state cannabis use did not change insignificantly. Model D repeated Model A, but without Status as a covariate to remove potential over-adjustment. In all four cases the change in the relative risk was tightly constrained in the range 1.14–1.26 (eTable 18).

**Fig. 3:**
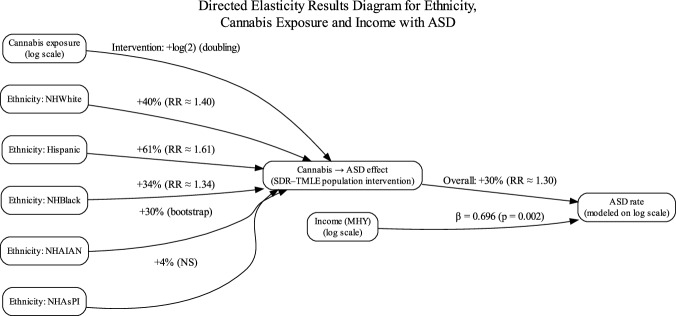
Directed Results Path Diagram for the relationship between ethnicity, cannabis exposure, and income with ASD.

Figure 3 presents all of these elasticity results as a Directed Path for Results Diagram.

Given the clearly non-linear relationship between many of these key parameters it was of interest to apply generalized additive modelling (GAM) to this analysis. Initially both ethnic cannabis and ethnicity are each highly significant with ethnic cannabis more significant (higher Chi Squared) than ethnicity (eTable 19). However, when both the tobacco: ethnicity and cannabis: ethnicity interactions are considered ethnicity loses its significance as a main effect but persists in interaction with cannabis and tobacco (model 3, Table 1). Similarly, ethnicity does not appear in the final GAM model where the three way ethnic tobacco: ethnic cannabis: ethnicity interaction appears and this model reduces to the same as previous (model 4, Table 1). When estimates of ethnic Δ9THC and cannabidiol were studied similar results were obtained (models 5 and 6 Table 1).

**Table 1:** Complex General Additive Models.

Parameters					Model	
Term	edf	ref.df	Statistic	*P*-Value	Metric	Value
*Full Additive Model*						
ASD ~ eCigarettes + eBng.Alcohol + eCannabis + eAnalgesics + eCocaine + Median.Income + Ethnicity						
Ethn.Cigarettes	2.90	2.99	23.3	3.38E-05	AIC	5654
Ethn.Cannabis	2.98	3.00	227.0	0	BIC	5785
Med.Income	7.89	8.66	126.0	0	LogLik	-2799
Ethn.Binge.Alcohol	8.18	8.68	129.0	0	Deviance	2298
Ethnicity	4.71	5.00	917.0	0	Adj.R.Squared	-4.95
*Interactions eCannabis: Race*						
ASD ~ eCigarettes + eBng.Alcohol + eCannabis + eAnalgesics + eCocaine + Median.Income + Ethnicity + Race: eCannabis						
Ethn.Cigarettes	2.90	2.99	23.3	3.38E-05	AIC	5654
Ethn.Cannabis	2.98	3.00	227.0	0	BIC	5785
Med.Income	7.89	8.66	126.0	0	LogLik	-2799
Ethn.Binge.Alcohol	8.18	8.68	129.0	0	Deviance	2298
Ethnicity	4.71	5.00	917.0	0	Adj.R.Squared	-4.95
*Interactions eCannabis: Race + eCigarettes: Race*						
ASD ~ eCigarettes + eBng.Alcohol + eCannabis + eAnalgesics + eCocaine + Median.Income + Ethnicity + Race: eCannabis + Race: eCigarettes						
Ethn.Cannabis	1.53	1.65	8.9	7.77E-03	AIC	2083
Med.Income	5.25	6.43	36.2	-3.21E-06	BIC	2223
Ethn.Analgesics	1.00	1.00	4.0	0.0454	LogLik	-1006
Ethn.Binge.Alcohol	5.43	6.42	20.1	4.16E-03	Deviance	557
Ethn.Cannabis: Race	8.10	23.00	74.8	0	Adj.R.Squared	-0.169
Ethn.Cigarettes: Race	13.20	24.00	47.7	0		
*Interactions 3 Way eCannabis: Race: eCigarettes*						
ASD ~ eCigarettes + eBng.Alcohol + eCannabis + eAnalgesics + eCocaine + Median.Income + Ethnicity + Race: eCannabis: eCigarettes						
Ethn.Cannabis	1.75	1.90	10.6	0.0044	AIC	1972
Med.Income	5.45	6.63	41.5	-3.60E-06	BIC	2119
Ethn.Analgesics	1.00	1.00	4.0	0.0444	LogLik	-949
Ethn.Binge.Alcohol	5.96	6.92	23.5	0.0016	Deviance	577
Ethn.Cannabis: Race	8.12	23.00	85.8	0	Adj.R.Squared	-0.0425
Ethn.Cigarettes: Race	14.1	24.00	55.9	0		
*Cannabinoids*						
*Interactions e*Δ*9THC: Race + eCigarettes: Race*						
ASD ~ eCigarettes + eBng.Alcohol + eCannabis + eAnalgesics + eCocaine + Median.Income + Ethnicity + Race: eΔ9THC + Race: eCigarettes						
Med.Income	2.15	2.52	16.9	0.0005	AIC	2096
Med.Income	5.54	6.74	52.5	0	BIC	2213
Ethn.Analgesics	1.00	1.00	8.8	0.0030	LogLik	-1018
Ethn.Binge.Alcohol	4.93	5.92	25.8	0.0002	Deviance	582
Ethn.Δ9THC: Race	15.00	24.00	499.0	0	Adj.R.Squared	-0.406
*Interactions eCBD: Race + eCigarettes: Race*						
ASD ~ eCigarettes + eBng.Alcohol + eCannabis + eAnalgesics + eCocaine + Median.Income + Ethnicity + Race: eCannabidiol + Race: eCigarettes						
Med.Income	4.89	6.05	47.0	0	AIC	2135
Ethn.Analgesics	1.00	1.00	20.3	7.43E-06	BIC	2285
Ethn.Binge.Alcohol	6.34	7.27	30.3	0.0001	LogLik	-1029
Ethn.Cannabidiol: Race	21.70	24.00	137.0	0	Deviance	603
Ethn.Cigarettes: Race	3.37	22	10.9	0.0067	Adj.R.Squared	-62

eCigarettes, eCannabis and Eth.Cigarettes, Eth.Cannabis etc.—Ethic Exposure to Cigarettes, Cannabis etc. as described in online Methods section.

*AIC* Akiake Information Criterion, *BIC* Bayesian Information Criterion, *LogLik.* Log Likelihood ration at model optimization, *Adj.R.Squared* Adjusted R Squared.

### Nevada

NBDPN data reveal that the ASD numbers in Nevada 2016–2020 in the NHWhite, NHBlack, NHAsPI and Overall populations were 5404, 2127, 1454, 13,821 associated with ASDRs of 796·1, 849·6, 884·0 and 772·8, respectively. Despite the NHAsPI group having the lowest ASDR across the country, the ASDR amongst this ethnicity in Nevada was the highest. As the highest rate in the country it was considered important to model this trend in detail.

It should be noted that the previous ASDR from this group was 367·9 in 2012-2016. Using exponential modelling it can be shown that this datum represents a doubling time of just 2·83 years.

ASDR data from Nevada were presented in eFig. 2 panel 4. These steep rises were modelled with mixed effects, survey and polynomial regression (eTable 20). In each case, both ethnic cannabis exposure and ethnicity were significant.

**Fig. 4:**
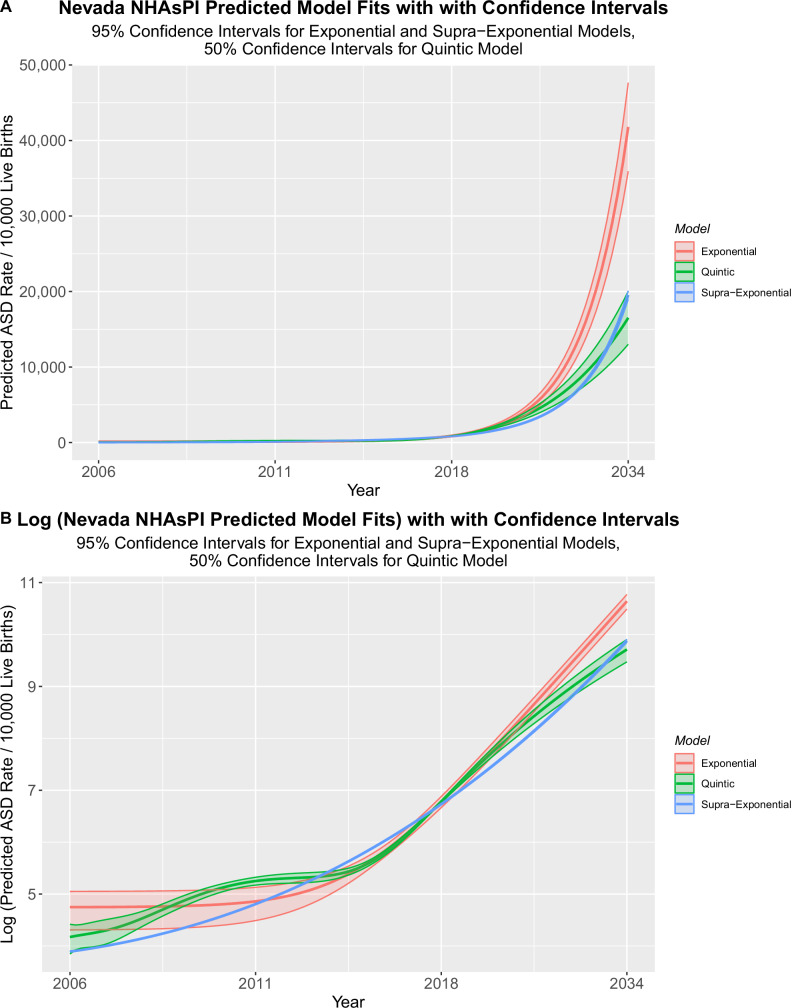
Mathematical Projections of ASD Rates amongst Nevadan Non-Hispanic Asian and Pacific Islander cohorts. Exponential, polynomial quintic and supra-exponential models shown on (**A**) linear and (**B**) logarithmic scales.

Predictive modelling using quintic, exponential and supra-exponential models was considered. Based upon residuals from the known values, the proximity to the 2016–2020 value and AIC the models were rated quintic > exponential > supra-exponential (eTable 21). Models are illustrated in Fig. 4 plotted on ordinal and log scales.

One point of obvious interest is the date at which the neonatal population will be saturated with ASD at 100% prevalence, assuming continuation of current trends. The dates predicted by the quintic, exponential and supra-exponential models were January 19th, 2031, August 6th, 2028 and October 7th, 2030.

## Discussion

### Main results

ASDRs amongst African Americans, American Indians, and Alaskan Natives were significantly higher compared to others ethnic groups respectively. Alarmingly, ASDRs amongst Asians and Pacific Islanders in Nevada showed an exponential doubling time of 2.83 years. Arguably one of the most arresting aspects of the present study is the significant increase of ASD from rare to common in just a few short years in states such as Nevada, Mississippi, Kentucky, and Hawaii. Further, study results provide strong evidence to consider the plausibility of a causal cannabinoid exposure link to ASD at the population and sub-population levels, and a strong foundation for limiting community exposure to cannabinoids across all ethnicities until certainty around these findings is firmly established. Until we more clearly understand dose thresholds at which different cannabinoids may cause congenital and epigenetic changes, not only for the general population but also for ethnic subgroups, neonates, adolescents, pregnant woman, and the elderly to mention only a few, community exposure to cannabinoids urgently need to be minimized.

An understanding of possible biological mechanisms by which cannabinoid exposure can cause ASD, inclusive of cannabinoid genotoxicity, and of study statistics is fundamental to asserting the plausibility of a population-level causal cannabinoid exposure interpretation. Accordingly, these are reviewed and discussed below.

### Nevada

One of the most dramatic findings of the present work was the extraordinary surge in ASDR across all ethnicities in Nevada, and the question naturally arises as to how cannabinoid exposure could so quickly give rise to significant increased incidents of ASD, and if such recent trends might continue, or occur in other jurisdictions.

Several factors and their culmination are likely contenders. Nevada has a publicly documented high level of cannabinoid use. Even a cursory inspection of the city streetscapes indicates that cannabis shops are common. Concern has also been expressed that food chain contamination (crop and animal contamination via waterways, and animal fodder) and percutaneous exposures (CDB skin creams, contaminated water supplies inclusive swimming pools) also constitute important contributing routes of human exposure. Models in the present study highlight that CBD is at least as potent a teratogen as Δ9THC, with both widely available Nevada [28].

Important to understanding the Nevada predicament is understanding that cannabinoids have a well-documented exponential genotoxic dose-response curve. Simply put, a small increment in cannabinoid exposure may result in a major genotoxic outcome, and it is possible that community cannabinoid exposure could rise into the micromolar range where genotoxic outcomes “suddenly” go from rare to become commonplace.

Of course, cannabinoids, including CBD and Δ9THC are also widely available in many other States of USA. It follows that if this exponentiation of genotoxic cannabinoid dose-response and threshold effect continues to be ignored, it may well result in significant morbidity in other US jurisdictions as observed in Nevada.

### Mechanisms

Space precludes a detailed consideration of the cardiogenic impacts of cannabinoids and the interested reader is referred elsewhere [3, 6, 16, 28]. Concisely, the heart and central great vessels are formed by a complex choreographed coalescence over time of cells from the primary, secondary and lateral heart fields, the proepicardium, dorsal mesocardium, nuchal crest and pharyngeal arches in a manner controlled by morphogenic gradients and epigenomic orchestration [6, 16]. Cannabinoids directly disrupt the genome, including chromosomal and DNA breaks, broadly perturb the epigenome and its machinery [9, 24–28] and most of the morphogens which together form the scaffolding upon which cardiogenesis occurs [17–23]. The atrial septum primum forms, degenerates, is largely replaced by the atrial septum secundum and the two gradually fuse anatomically in life’s first year [16]. This delicate process is clearly susceptible to disruption at multiple points.

Cannabinoids have been shown to interact with multiple morphogens, including sonic hedgehog, fibroblast growth factor, including transactivation of the FGF1R by CB1R; bone morphogenetic proteins, retinoic acid signalling, notch signalling (which is very involved in colorectal cancer), Wnt signalling and the hippo pathway.

It is of interest to consider one key morphogen by way of example. The Sonic Hedgehog (Shh) signalling pathway plays a central role in embryonic cardiogenesis by regulating cardiac progenitor proliferation, secondary heart field (SHF) specification, atrial septation and outflow tract development. Shh ligand, secreted from the notochord and pharyngeal endoderm, binds to the transmembrane receptor PTCH1. In the absence of Shh, PTCH1 inhibits Smoothened (SMO), a key signal transducer. Shh binding relieves this inhibition, activating SMO and initiating a cascade that leads to the regulation of GLI transcription factors (GLI1, GLI2, GLI3). These GLI proteins translocate to the nucleus to control expression of genes essential for cardiac development, including those promoting progenitor proliferation such as *Cyclin D1*.

In the SHF, Shh signalling ensures expansion of cardiac progenitors contributing to the atrial myocardium, right ventricle and outflow tract. Shh signalling also influences the endocardial cushions and epithelial-to-mesenchymal transition which is involved in atrioventricular septation. Several workers have confirmed the involvement of Shh in atrial septation. Loss of Shh or downstream components like GLI2 results in hypoplasia of these regions and conotruncal defects. Additionally, Shh influences cardiac neural crest cell survival and migration, indirectly supporting outflow tract septation. The pathway also establishes left-right asymmetry, a prerequisite for correct heart tube looping, through its interaction with *Nodal* and *Pitx2* expression. Moreover Shh interacts with retinoid morphogen and signalling pathways to control cardiac developmental dextrorotation and normal cardiopulmonary asymmetries through their dual control of Lefty-1. Altogether, the PTCH1–SMO–GLI axis acts as a molecular switch integrating spatial cues to regulate cardiogenic gene networks. Disruption of any component leads to structural congenital heart defects, highlighting Shh signalling as a master regulator of heart morphogenesis.

Studies have shown that cannabinoids Δ9THC and cannabidiol directly inhibit Shh and epigenomically inhibit Shh, PTCH1, SMO and GLI [24].

### Cannabinoid genotoxicity

Cannabis has been linked with 44 of 64 congenital anomalies in USA [7, 9] and 89 of 95 anomalies in Europe by population-wide studies [53]. Early termination of pregnancy for anomaly (ETOPFA) is practised for many of these major anomalies so that their true rates are often difficult to ascertain. This implies that the present results for ASD where ETOPFA does not occur may well have wider bellwether implications for many other congenital anomalies more broadly.

Moreover cannabinoid teratogenicity is just one aspect of cannabinoid genotoxicity more broadly which may be expected to also manifest in cancerogenesis, mental retardation and aging. Thus the present results in relation to ASD may have broad public health implications across many health domains.

### Statistical analysis

Study data show that the significant effects of ethnicity on ASDR are greatly reduced when ethnic exposure to cannabis and the cannabinoids Δ9THC and cannabidiol are considered and of the two, cannabinoids predominates over ethnicity. Both between- and within- state effects are significant with the former being four times more powerful than the latter. In GAM modelling, the main effect of ethnicity disappears and only interactive terms between ethnicity, cannabis and cannabinoids remain in final models.

This study presents a striking convergence of findings from multiple regression analytical frameworks including mixed effects, correlated random effects of Mundlak, panel, negative binomial general additive models (GAM), sequentially doubly robust targeted maximum likelihood estimation (SDR–TMLE), cluster robust interference, policy restriction sensitivity, variability restriction sensitivity, over-adjustment sensitivity, and difference-in-difference designs which together provide powerful triangulated confirmation of the robustness of the cannabis-ASDR link. Data are consistent with spatiotemporal analyses reported elsewhere [3, 9, 54]. The SDR–TMLE models are particularly powerful and have become the current gold standard for causal inference. Mixed effects models were inverse probability weighted, which is also a powerful inferential strategy. Ethic heterogeneity was prominent, with the largest ethnic quantum being seen in NHAIAN groups and the largest regression slope elasticity seen amongst people of Hispanic background. Findings were directionally consistent with regression-based elasticity conversions and persisted across robustness checks.

The convergence of findings across structurally distinct modelling approaches strengthens the plausibility of a population-level causal interpretation under the assumptions of no unmeasured confounding, positivity, and correct specification within the doubly robust framework. SDR–TMLE is particularly relevant because it combines outcome regression and exposure modelling and remains consistent if either nuisance model is correctly specified. State-clustered inference further accounts for within-state dependence.

Methods of case ascertainment and legal status were not a primary focus of this study but are investigated in detail in other manuscripts [3, 54, 55]. These covariates were used in sophisticated mixed effects and panel analyses as described.

### Causality

LMTP models directly address the central conundrum of causal inference relating to “What if …” the counterfactual scenario had occurred under the treatment assumptions. LMTP sequentially doubly robust TMLE (SDR-TMLE) models are widely regarded as a gold standard for causal inference with continuous exposures because they estimate causal effects under realistic modified treatment policies while remaining doubly robust, meaning the estimator is consistent if either the exposure model or the outcome model is correctly specified. By combining machine learning for flexible confounder adjustment with targeted estimation that yields valid statistical inference, SDR-TMLE reduces model-misspecification bias and achieves near-optimal efficiency compared with conventional regression approaches.

#### Explicit causal identification assumptions

Causal interpretation requires (i) consistency, (ii) positivity (sufficient overlap in exposure distributions across covariates and time), (iii) conditional exchangeability given measured covariates and (iv) correct specification of at least one nuisance model: SDR–TMLE remains consistent if either the outcome model or exposure model is correctly specified. Because unmeasured state-level factors (e.g., diagnostic intensity, policy/culture) may influence both cannabis exposure and ASD ascertainment, results are interpreted as population intervention effects under measured covariate control and are complemented by regression and panel robustness checks.

The present ASD-cannabinoid findings fulfil the classical Hill criteria of causality [56], including strength of association, consistency, specificity, temporality, biological gradient, plausibility, coherence, experiment and analogy as shown by the present and other studies [3–9]. Strong evidence for multiple biological pathways makes the mechanistic argument particularly powerful.

Both mixed effects and GAM models shown were inverse probability weighted. This has the effect of making the populations from which they were drawn pseudorandomized. Results can therefore be considered within a causal framework for epidemiological studies analogous to those from randomized controlled trials.

### Generalizability

The present results are widely generalizable due to the multiplicity of biological mechanisms implicated, their applicability across ethnicities, their concordance with space-time studies [3, 9, 54] and their consistency with external studies [3–9].

Mechanistically, there is also reason to suggest that present findings may relate more broadly to other areas of cannabinoid teratogenicity [7] and genotoxicity, including mental illness [10], aging [27, 28], carcinogenicity and autism as has previously been demonstrated.

### Limitations

In line with many epidemiological studies individual participant data were not available. The ecological structure of state-level exposure data precludes individual-level causal inference. State-level cannabinoid exposure level was similarly unavailable. States reporting low and zero ASDRs may need to address issues of data quality and data completeness. Space-time considerations are addressed elsewhere [3, 9, 54]. Residual confounding by unmeasured policy, gestational timing, environmental, or cultural factors cannot be excluded as some covariates were not included in the present analysis and could be addressed in subsequent work.

## Conclusion

Advanced modelling techniques reveal that ethnic exposure to cannabinoids is a more powerful predictor of ASDR than ethnicity itself. Both Δ9THC and cannabidiol were directly implicated, and other cannabinoids are also likely to be involved. Triangulation across mixed effects, panel, semiparametric, general additive, causal SDR–TMLE and spatial models increases confidence that the observed association is not an artifact of a single modelling strategy. The consistency of direction, relative magnitude, and ethnicity modification across frameworks supports the robustness of the population-level findings. Temporally exponentiating ASDR curves appear to be following well described exponential cannabinoid genotoxic dose-response curves, and the two fulfill both quantitative and qualitative criteria for being causally related in the forward direction. Recent surges where ASDR has moved from rare to common in Nevada and elsewhere clearly illustrate the public health impacts of this exponentiation. That ASD is likely a bellwether marker for other forms of cannabinoid teratogenicity and genotoxicity more generally, the multiplicity of cannabinoids involved, the ubiquity of cannabinoid exposure in the current US context, potential food chain contamination and the intergenerational nature of this disorder amplify present concerns.

## Supplementary information

Supplementary Materials_Ethnicity - Figures 1–9 & Tables 1-21

## Data Availability

Data and computational code are openly available via Mendeley at 10.17632/pbbzkbd562.3.
