# Supplementary material for: Cannabinoid impacts on ethnic modulation of atrial septal defect prevalence USA
Source: J Perinatol. 2026 May 27;46(7):1323–33. doi: 10.1038/s41372-026-02717-7 (PMC13423878; doi:10.1038/s41372-026-02717-7)
Supplement: Supplementary file 1 — Supplementary Materials_Ethnicity - Figures 1–9 & Tables 1-21 [file 41372_2026_2717_MOESM1_ESM.pdf]

SUPPLEMENTARY TABLES

List of eTables

eTable 1.: Sociodemographic and Substance Exposure Data dichotomized by ethnicity.

eTable 2.: ASD Rates by Ethnicity and State.

eTable 3.: Average ASD rates by Ethnicity and year.

eTable 4.: Mixed Effects regression summary of ASD Rate against ethnicity. Comparator group is NHAsPI.

eTable 5.: Mixed effects Additive and Interactive models for cannabis exposure against both ethnicity and dichotomized ethnicity.

eTable 6.: Mixed effects model of ASDR against ethnicity, cannabis exposure, income, case ascertainment method and cannabis legal status including an interaction between cannabis and ethnicity.

eTable 7.: Hybrid mixed effects modelling of cannabis exposure as both a within-state and between-state covariate. Bivariate, additive, interactive and Mundlak models.

eTable 8.: Model Comparison table for the Overall Model Parameters of eTable 5. See also eFigure 5.

eTable 9.: Mixed effects analysis of dichotomized ethnicity.

eTable 10.: Mixed effects analysis of ethnic Δ9THC exposure.

eTable 11.: Effects sizes for Model 4 from eTable 8 for ethnicity as a function of both Δ9THC and cannabidiol.

eTable 12.: Effects sizes for Model 4 from eTable 8 for contrasts between the different levels of ethnicity as a function of both Δ9THC and cannabidiol.

eTable 13.: Comparative slopes for the ASDR-cannabis relationship from mixed effects models for historical data (from R package emmeans).

eTable 14.: Comparative slopes for the ASDR-cannabis relationship from mixed effects models for historical data together with predictions of the percentage effect of 50% and 100% rises in cannabis exposure (from R package emmeans).

eTable 15.: Panel regression models comparing within and between state variations in the ASDR-cannabis relationship (from R package plm).

eTable 16.: Difference-in-Difference analyses of ASDR-cannabis relationship after a change in cannabis legal status by estimates (upper table) and by P-value (lower table).

eTable 17.: Effects of doubling cannabis exposure under a sequentially doubly robust (SDR) targeted maximum likelihood estimation (TMLE) analytical framework by ethnicity.

eTable 18.: Sensitivity analysis of four models: (A) Main analysis of a scenario involving doubling cannabis exposure in a sequentially doubly robust (SDR) targeted maximum likelihood estimation (TMLE) framework; (B) same but only looking at states where the legalization status changed, (C) same but excluding states manifesting minimal within state change in cannabis and (D) same as (A) but without legal status in the model thereby correcting for over-adjustment.

eTable 19.: Generalized additive regression, Introductory models.

eTable 20.: Modelling of ASD Rates in Nevada by ethnic cannabis exposure as mixed effects, survey regression, and polynomial quintic and quadratic functions. A quadratic function was used as only the first two levels of the quintic function were statistically significant.

eTable 21.: Historical and predicted ASD rates for each of the exponential, quintic and supra-exponential models.

eTable 1.: Sociodemographic and Substance Exposure Data Dichotomized by Ethnicity

Measure	NHAA_AIAN	Other	P-Value	ΔSMD
Sample Size (N)	581	1301		
ASD Rate (median [IQR])	64.90 [32.70, 129.20]	41.70 [23.60, 92.60]	<0.001	0.353
Log (ASD Rate) (median [IQR])	4.17 [3.49, 4.86]	3.73 [3.16, 4.53]	<0.001	0.355
Births (mean (SD))	725.51 (1537.19)	30102 (64944)	0.945	0.355
Numbers Atrial Septal Defect (Total)	40,965	365,928	<0.001	0.232
Births (Total)	5,095,686	60,522,566	0.587	0.003
Cigarettes, Monthly (median [IQR])	0.25 [0.23, 0.28]	0.25 [0.22, 0.28]	0.559	0.033
Alcohol, Monthly (median [IQR])	0.57 [0.51, 0.61]	0.57 [0.51, 0.61]	0.795	0.001
Alcoholism (median [IQR])	0.07 [0.06, 0.08]	0.07 [0.06, 0.08]	0.752	0.019
Binge Alcohol (median [IQR])	0.25 [0.22, 0.27]	0.25 [0.22, 0.27]	0.684	0.017
Cannabis, Monthly (median [IQR])	0.06 [0.05, 0.08]	0.06 [0.05, 0.08]	0.943	0.010
Analgesics, Annual (median [IQR])	0.04 [0.04, 0.05]	0.04 [0.04, 0.05]	0.660	0.027
Cocaine, Annual (median [IQR])	0.02 [0.01, 0.02]	0.02 [0.01, 0.02]	0.998	0.007
Δ9THC Content (median [IQR])	12.30 [8.76, 14.10]	12.30 [8.76, 14.10]	0.856	0.012
Cannabidiol (median [IQR])	0.28 [0.20, 0.46]	0.28 [0.20, 0.46]	0.867	0.008
Cannabigerol (median [IQR])	0.43 [0.40, 0.46]	0.43 [0.40, 0.46]	0.952	0.003
Cannabinol (median [IQR])	0.45 [0.37, 0.63]	0.45 [0.37, 0.63]	0.838	0.009
Cannabichromene (median [IQR])	0.24 [0.24, 0.26]	0.24 [0.24, 0.26]	0.837	0.008
Tetrahydrocannabivarin (median [IQR])	0.09 [0.09, 0.10]	0.09 [0.09, 0.10]	0.938	0.003
Δ9THC * Cannabis, Monthly (median [IQR])	0.73 [0.51, 1.05]	0.73 [0.52, 1.04]	0.891	0.004
Median Income (median [IQR])	50721 [45407, 57598]	51006 [45407, 57810]	0.650	0.027
Ethnic Cigarette Exposure (median [IQR])	1.06 [0.96, 1.40]	0.83 [0.68, 1.09]	<0.001	1.379
Ethnic Analgesic Exposure (median [IQR])	1.07 [0.94, 1.94]	0.98 [0.85, 1.03]	<0.001	1.257
Ethnic Binge Alcohol (median [IQR])	0.89 [0.82, 0.93]	0.96 [0.87, 1.08]	<0.001	0.253
Ethnic Cannabis Exposure (median [IQR])	1.19 [1.17, 1.23]	0.81 [0.66, 1.06]	<0.001	1.862
Ethnic Analgesic Exposure (median [IQR])	0.92 [0.77, 1.36]	0.96 [0.78, 1.06]	<0.001	0.780
Ethnic Cocaine Exposure (median [IQR])	0.88 [0.81, 1.15]	0.97 [0.85, 1.05]	0.613	0.620
Legal Status of Cannabis (%)			0.874	0.041
Medical	66 (11.4)	132 (10.1)		
Legal	356 (61.3)	813 (62.5)		
Illegal	22 (3.8)	47 (3.6)		
Decriminalized	137 (23.6)	309 (23.8)		
Number Illegal (%)	225 (38.7)	488 (37.5)	0.652	0.025
Number Legal (%)	559 (96.2)	1254 (96.4)	0.958	0.009

Table Key: IQR – Interquartile range; NH_AA_AIAN – Non-Hispanic African American and Non-Hispanic American Indican Alaska Natives; Other – Other ethnicities.

eTable 2.: ASD Rates by Ethnicity and State.

State	Year	Race	ASD_Rate
Nevada	2018	NHAsPI	884.00
Nevada	2018	NHBlack	849.60
Mississippi	2013	NHAIAN	802.00
Nevada	2018	NHWhite	796.10
Nevada	2018	Total	772.80
Mississippi	2014	NHAIAN	739.10
Nevada	2018	Hispanic	714.30
Nevada	2018	NHAIAN	595.10
Kentucky	2018	NHBlack	568.30
Hawaii	2007	NHBlack	562.30
Kentucky	2018	NHAsPI	552.80
Kentucky	2014	NHAsPI	523.00
Kentucky	2010	NHBlack	520.40
Kentucky	2013	NHAsPI	519.90
Hawaii	2007	Hispanic	481.90
North Dakota	2005	NHBlack	478.70
Mississippi	2011	NHAIAN	469.20
Mississippi	2012	NHAIAN	466.10
Kentucky	2014	NHBlack	466.00
Vermont	2012	NHAIAN	465.10
Kentucky	2013	NHBlack	463.40
Kentucky	2012	NHBlack	453.40
Nevada	2014	NHBlack	444.10
Kentucky	2012	NHAsPI	405.40
Mississippi	2010	NHAIAN	378.70
Kentucky	2011	NHBlack	372.50
Nevada	2014	NHAsPI	367.90
Nevada	2014	Total	367.90
Nevada	2014	NHWhite	365.30
Kentucky	2014	NHAIAN	357.10
Hawaii	2006	NHBlack	349.70
New Mexico	2018	NHAIAN	349.40
Kentucky	2018	Hispanic	344.60
Oregon	2012	NHAIAN	336.80
New Mexico	2018	NHBlack	334.90
Kentucky	2018	Total	332.80
North Dakota	2006	NHBlack	332.20
Hawaii	2005	Hispanic	330.00
Nevada	2013	NHBlack	329.80
Mississippi	2014	NHBlack	323.40
New Mexico	2018	NHAsPI	321.30
Kentucky	2007	NHBlack	317.40

New Mexico	2018	Total	316.50
Oregon	2014	NHAIAN	315.90
New Mexico	2018	Hispanic	313.40
New Mexico	2018	NHWhite	306.00
Tennessee	2018	NHBlack	305.70
Oregon	2013	NHAIAN	305.60
Nevada	2014	Hispanic	304.20
Kentucky	2013	NHAIAN	301.90
Kentucky	2018	NHWhite	300.10
West Virginia	2018	NHBlack	295.90
Alaska	2018	NHAIAN	295.80
Kentucky	2014	Total	295.30
Kentucky	2012	Total	293.40
Hawaii	2005	NHBlack	292.60
Kentucky	2009	NHBlack	291.70
Mississippi	2013	NHBlack	290.60
Kentucky	2006	NHBlack	290.00
Kentucky	2005	NHBlack	285.60
Kentucky	2013	Total	283.90
Oregon	2014	NHBlack	282.90
Mississippi	2013	Hispanic	280.20
Vermont	2009	NHAIAN	277.80
Kentucky	2006	NHAIAN	277.80
Oregon	2018	NHBlack	276.30
Kentucky	2018	NHAIAN	275.60
Mississippi	2009	NHAIAN	275.50
Nevada	2013	Total	273.10
Mississippi	2014	Total	271.20
Oregon	2012	NHBlack	269.90
Kentucky	2014	NHWhite	269.50
North Dakota	2012	NHBlack	269.20
Nevada	2013	NHWhite	268.60
Ohio	2014	NHAIAN	266.80
Hawaii	2006	Hispanic	266.00
Mississippi	2014	Hispanic	264.50
Kentucky	2012	NHWhite	264.40
Kentucky	2013	NHWhite	263.70
Kentucky	2014	Hispanic	261.90
Oregon	2013	NHBlack	261.40
West Virginia	2018	NHAIAN	256.40
Alaska	2014	NHAIAN	251.70
Michigan	2018	NHBlack	251.60
North Dakota	2012	NHAIAN	250.80
Mississippi	2013	Total	250.10
Nevada	2013	NHAsPI	245.70
North Dakota	2011	NHAIAN	244.20

Vermont	2010	NHAIAN	243.90
Vermont	2011	NHAIAN	243.90
Alaska	2013	NHAIAN	243.20
Oregon	2018	NHAIAN	242.40
Kentucky	2012	NHAIAN	240.50
Alaska	2012	NHAIAN	240.30
Nevada	2011	NHBlack	240.30
New York	2018	NHBlack	239.50
Tennessee	2014	NHBlack	238.10
Oregon	2014	Hispanic	230.40
North Dakota	2011	NHBlack	226.80
Alaska	2007	NHAIAN	224.90
Mississippi	2014	NHWhite	224.60
Tennessee	2013	NHBlack	223.00
Nevada	2010	NHBlack	222.80
Oregon	2018	Hispanic	221.60
North Dakota	2010	NHAIAN	220.80
Ohio	2014	NHBlack	220.60
Missouri	2018	NHBlack	219.90
Oregon	2013	Hispanic	218.50
Kentucky	2011	Total	217.60
Tennessee	2018	Total	217.30
Tennessee	2012	NHBlack	213.60
Colorado	2018	NHAIAN	213.50
Oregon	2012	Hispanic	212.70
Missouri	2010	NHAIAN	211.60
West Virginia	2011	NHBlack	209.90
Nevada	2013	Hispanic	209.20
Alaska	2014	NHBlack	207.70
Michigan	2018	Total	207.70
Kentucky	2010	Total	206.90
Tennessee	2011	NHBlack	204.70
Kentucky	2011	NHWhite	204.50
Missouri	2013	NHBlack	202.80
Michigan	2013	NHAsPI	201.70
Colorado	2012	NHAIAN	200.10
Tennessee	2018	NHWhite	199.70
Missouri	2018	NHAIAN	199.60
North Dakota	2009	NHAIAN	199.30
Alaska	2013	NHBlack	196.20
Tennessee	2010	NHBlack	196.00
Mississippi	2013	NHWhite	195.90
West Virginia	2010	NHBlack	193.10
Missouri	2011	NHAIAN	192.30
Tennessee	2018	NHAIAN	190.50
Nevada	2014	NHAIAN	190.20

Alaska	2012	NHAsPI	188.30
Missouri	2012	NHBlack	187.70
Colorado	2011	NHBlack	187.20
Michigan	2013	NHBlack	187.10
Kentucky	2009	Total	187.00
Kentucky	2013	Hispanic	186.20
Tennessee	2009	NHBlack	185.70
Michigan	2014	NHBlack	185.50
Oregon	2018	Total	185.40
Vermont	2013	NHAIAN	185.20
Alaska	2012	NHBlack	185.00
Kentucky	2012	Hispanic	184.50
Oregon	2014	Total	183.70
Missouri	2011	NHBlack	182.90
Missouri	2010	NHBlack	181.70
Michigan	2018	NHWhite	181.50
Mississippi	2011	NHBlack	180.30
Tennessee	2018	NHAsPI	180.20
Michigan	2013	NHAIAN	179.20
Oregon	2013	Total	179.20
Vermont	2014	NHAIAN	178.60
Tennessee	2018	Hispanic	178.50
Michigan	2018	Hispanic	177.90
Alaska	2005	NHAIAN	177.60
Florida	2014	NHAIAN	176.80
Oregon	2018	NHWhite	176.10
Colorado	2010	NHBlack	175.70
Alaska	2013	NHAsPI	175.40
Alaska	2014	Total	175.20
Michigan	2018	NHAIAN	175.20
Colorado	2012	NHBlack	175.10
Kentucky	2006	NHAsPI	175.10
West Virginia	2018	Hispanic	174.90
West Virginia	2018	Total	174.00
Alaska	2014	Hispanic	173.50
Nevada	2011	NHAsPI	173.00
Oregon	2012	Total	173.00
West Virginia	2011	Total	172.40
Alaska	2018	Total	172.10
West Virginia	2011	NHWhite	171.60
Alaska	2013	Total	171.10
Tennessee	2014	Total	170.70
West Virginia	2018	NHWhite	170.70
Ohio	2014	Total	170.50
Kentucky	2007	Total	170.30
New York	2018	Hispanic	169.90

Virginia	2010	Hispanic	169.60
Florida	2012	NHAIAN	168.30
Oregon	2014	NHWhite	168.10
Nevada	2011	Total	167.10
Mississippi	2012	NHBlack	166.90
Nevada	2009	NHBlack	166.80
Vermont	2018	NHAIAN	166.70
Colorado	2013	NHAIAN	166.60
Oregon	2013	NHWhite	165.50
Kentucky	2010	NHWhite	165.30
Nevada	2010	NHAsPI	165.00
Missouri	2014	NHBlack	164.00
Kentucky	2009	NHAsPI	163.40
West Virginia	2012	NHBlack	163.30
Colorado	2013	NHBlack	163.00
Virginia	2009	Hispanic	162.20
Alaska	2014	NHAsPI	162.10
Ohio	2014	Hispanic	162.00
Missouri	2018	Total	161.90
North Dakota	2010	NHBlack	161.60
Kentucky	2006	Total	161.60
Nevada	2010	Total	161.30
Alaska	2013	Hispanic	161.20
Florida	2013	NHAIAN	161.20
Missouri	2012	NHAIAN	161.10
Ohio	2014	NHWhite	161.00
Virginia	2013	Hispanic	159.50
West Virginia	2010	Total	159.40
Virginia	2006	Hispanic	159.10
West Virginia	2012	Total	158.70
Nevada	2011	NHWhite	158.60
Alaska	2012	Total	158.30
Oregon	2012	NHWhite	157.80
Colorado	2011	NHAIAN	157.70
West Virginia	2012	NHWhite	157.50
Tennessee	2014	NHWhite	157.30
Tennessee	2013	Total	157.20
Virginia	2012	Hispanic	156.90
West Virginia	2014	NHBlack	156.60
Colorado	2009	NHBlack	156.40
North Dakota	2007	NHAIAN	156.20
Virginia	2011	Hispanic	156.10
Kentucky	2010	NHAsPI	155.70
Mississippi	2011	Total	155.50
Mississippi	2013	NHAsPI	155.50
Florida	2011	NHAIAN	155.30

Virginia	2010	NHAsPI	155.20
Virginia	2013	NHAIAN	154.40
West Virginia	2010	NHWhite	153.60
Kentucky	2005	Total	153.10
Missouri	2018	Hispanic	152.60
Louisiana	2006	NHAIAN	152.20
Kentucky	2007	NHWhite	152.10
New York	2018	Total	152.00
Michigan	2012	NHBlack	151.80
Nevada	2010	NHWhite	151.70
Nevada	2011	Hispanic	151.70
New York	2018	NHAsPI	151.60
Mississippi	2010	NHBlack	151.30
Missouri	2018	NHWhite	151.30
Louisiana	2018	NHAIAN	150.90
Virginia	2018	NHBlack	150.70
Nevada	2010	Hispanic	150.40
Virginia	2011	NHAsPI	150.20
Missouri	2013	Total	149.90
Alaska	2018	Hispanic	149.20
Alaska	2018	NHAsPI	148.70
Alaska	2007	Total	148.60
Tennessee	2012	Total	148.60
North Dakota	2007	NHBlack	148.10
Virginia	2009	NHAsPI	147.50
Missouri	2018	NHAsPI	147.30
Vermont	2012	Hispanic	147.10
Vermont	2011	Hispanic	146.30
Alaska	2012	NHWhite	146.00
Missouri	2007	NHBlack	145.90
Mississippi	2012	Total	145.80
West Virginia	2013	NHWhite	145.80
Colorado	2014	NHAIAN	145.50
Illinois	2014	NHAIAN	145.20
Kentucky	2009	NHWhite	144.90
Kentucky	2006	NHWhite	144.40
Nevada	2013	NHAIAN	144.40
Tennessee	2013	NHWhite	144.30
West Virginia	2013	Total	144.10
Colorado	2011	NHAsPI	142.10
Michigan	2014	NHAIAN	141.50
Michigan	2018	NHAsPI	141.40
Michigan	2011	NHBlack	141.10
Missouri	2013	NHWhite	141.00
Michigan	2012	NHAIAN	140.90
Missouri	2007	NHAIAN	140.60

Kentucky	2011	Hispanic	140.50
Kentucky	2007	NHAIAN	139.70
Colorado	2018	NHBlack	139.60
Tennessee	2011	Total	139.50
Missouri	2012	Total	139.40
Illinois	2013	NHAIAN	138.40
Colorado	2007	NHBlack	138.30
Colorado	2011	Hispanic	137.90
Mississippi	2010	Total	137.60
Tennessee	2014	Hispanic	137.50
Michigan	2011	NHAIAN	137.20
Colorado	2012	Hispanic	137.00
Mississippi	2014	NHAsPI	137.00
Missouri	2011	Total	136.90
West Virginia	2014	NHWhite	136.90
Florida	2012	Hispanic	136.50
Alaska	2014	NHWhite	136.40
Tennessee	2010	Total	136.40
West Virginia	2014	Total	136.40
Mississippi	2007	NHAIAN	136.10
Tennessee	2012	NHWhite	135.60
Virginia	2013	NHBlack	135.60
Missouri	2013	Hispanic	135.50
Kentucky	2005	NHWhite	135.20
West Virginia	2013	NHBlack	135.20
Kentucky	2007	Hispanic	135.00
Florida	2011	Hispanic	134.80
Hawaii	2011	Hispanic	134.20
Hawaii	2012	Hispanic	134.20
Hawaii	2013	Hispanic	134.20
Missouri	2010	Total	134.20
Hawaii	2007	NHAsPI	134.10
Colorado	2011	Total	134.00
Florida	2010	Hispanic	134.00
Oregon	2014	NHAsPI	133.90
Kentucky	2006	Hispanic	133.80
Colorado	2012	NHAsPI	133.50
Colorado	2014	Total	133.20
Virginia	2007	NHAsPI	133.20
Kentucky	2009	Hispanic	132.80
Michigan	2014	Total	132.80
Kentucky	2005	NHAIAN	132.40
Florida	2013	Hispanic	132.30
Virginia	2018	Hispanic	132.20
Kentucky	2010	Hispanic	131.80
Colorado	2010	NHAsPI	131.60

Missouri	2012	NHWhite	131.60
Oregon	2018	NHAsPI	131.60
Mississippi	2011	NHWhite	131.30
Florida	2013	NHBlack	131.10
Virginia	2006	NHAsPI	130.90
Colorado	2013	Hispanic	130.90
Colorado	2013	Total	130.90
Florida	2012	NHBlack	130.50
Alaska	2018	NHWhite	130.40
Tennessee	2007	NHBlack	130.30
New York	2018	NHAIAN	130.20
Alaska	2013	NHWhite	129.90
Colorado	2012	Total	129.80
Missouri	2011	NHWhite	129.50
Alaska	2018	NHBlack	129.20
Florida	2010	NHBlack	129.20
Florida	2011	NHBlack	128.80
Louisiana	2006	NHWhite	128.40
Michigan	2013	Total	128.10
Florida	2014	NHBlack	127.90
North Dakota	2012	Total	127.50
Missouri	2010	NHWhite	127.40
Hawaii	2007	Total	127.20
Vermont	2010	Hispanic	127.20
Colorado	2014	NHBlack	127.10
Colorado	2009	NHAsPI	126.80
Virginia	2013	NHAsPI	126.80
Vermont	2013	Hispanic	126.60
Tennessee	2011	NHWhite	126.50
Tennessee	2009	Total	126.40
Virginia	2011	NHBlack	126.30
Colorado	2010	Hispanic	126.00
Florida	2014	Hispanic	126.00
Tennessee	2013	Hispanic	126.00
Colorado	2013	NHAsPI	125.70
Virginia	2010	NHBlack	125.70
Missouri	2014	Total	125.40
North Dakota	2012	NHAsPI	125.30
Oregon	2013	NHAsPI	125.30
Missouri	2012	Hispanic	125.20
Hawaii	2005	NHAsPI	125.00
Virginia	2012	NHAsPI	124.50
Tennessee	2010	NHWhite	124.40
Louisiana	2018	NHBlack	124.30
New Jersey	2010	NHAIAN	124.30
Colorado	2010	Total	124.10

Colorado	2011	NHWhite	124.10
Kentucky	2007	NHAsPI	124.00
Michigan	2014	Hispanic	123.90
Missouri	2011	Hispanic	123.60
Ohio	2014	NHAsPI	123.60
Colorado	2012	NHWhite	123.50
Missouri	2013	NHAsPI	123.50
Tennessee	2014	NHAIAN	123.00
Missouri	2014	Hispanic	122.90
North Dakota	2009	NHBlack	122.20
Mississippi	2009	NHBlack	122.00
Tennessee	2012	Hispanic	121.60
New Jersey	2009	NHAIAN	121.50
Virginia	2009	NHBlack	121.10
Mississippi	2012	NHWhite	120.50
Tennessee	2013	NHAIAN	120.50
Virginia	2018	Total	120.50
Alaska	2005	Total	120.20
Alaska	2007	NHWhite	119.90
Missouri	2014	NHAsPI	119.80
Kentucky	2005	Hispanic	119.60
Florida	2012	Total	119.40
West Virginia	2009	Total	119.10
Michigan	2013	Hispanic	118.70
West Virginia	2010	NHAsPI	118.70
New York	2014	NHBlack	118.30
Michigan	2010	NHBlack	118.00
Missouri	2014	NHWhite	118.00
Virginia	2012	NHBlack	117.90
Colorado	2010	NHWhite	117.80
New York	2018	NHWhite	117.80
Florida	2011	Total	117.70
Nevada	2009	NHAsPI	117.70
Florida	2010	NHAIAN	117.50
Missouri	2007	NHAsPI	117.50
Florida	2013	Total	117.40
Virginia	2010	Total	116.90
Colorado	2013	NHWhite	116.80
Virginia	2013	Total	116.50
Maine	2011	Hispanic	116.30
Michigan	2007	NHBlack	116.20
Florida	2010	Total	116.00
Colorado	2010	NHAIAN	115.90
Nevada	2009	Total	115.20
Florida	2014	Total	114.90
Michigan	2009	NHBlack	114.80

North Dakota	2012	Hispanic	114.70
Missouri	2007	Total	114.60
Tennessee	2014	NHAsPI	114.10
Tennessee	2009	NHWhite	114.00
Hawaii	2006	NHAsPI	113.70
Colorado	2009	Hispanic	113.60
Virginia	2007	Hispanic	113.50
Virginia	2009	Total	113.40
Hawaii	2005	Total	113.30
Tennessee	2011	Hispanic	113.20
Colorado	2018	Hispanic	113.10
Missouri	2010	Hispanic	113.10
Mississippi	2010	NHWhite	113.00
Vermont	2018	NHAsPI	113.00
Colorado	2009	Total	112.80
Michigan	2009	NHAIAN	112.70
Colorado	2018	Total	112.10
Michigan	2014	NHWhite	112.10
Oregon	2012	NHAsPI	112.10
Virginia	2011	Total	111.90
North Dakota	2012	NHWhite	111.80
Louisiana	2006	Total	111.10
Michigan	2013	NHWhite	111.00
Vermont	2014	Hispanic	110.70
Louisiana	2018	Total	110.40
Tennessee	2010	Hispanic	110.40
Virginia	2007	Total	110.30
Colorado	2014	Hispanic	110.10
Virginia	2018	NHWhite	110.10
North Dakota	2006	NHAIAN	109.70
Michigan	2006	NHBlack	109.50
Vermont	2014	NHBlack	109.40
Mississippi	2009	Total	109.20
Missouri	2007	NHWhite	109.00
Colorado	2018	NHWhite	108.90
Missouri	2012	NHAsPI	108.90
Colorado	2005	NHBlack	108.60
Vermont	2018	NHBlack	108.10
Nevada	2009	Hispanic	107.80
Maine	2012	Hispanic	107.50
Virginia	2018	NHAIAN	107.50
Virginia	2006	Total	107.10
Colorado	2009	NHWhite	106.80
Louisiana	2014	NHAIAN	106.70
Vermont	2010	NHBlack	106.70
Vermont	2018	Total	106.50

New York	2013	NHBlack	106.20
Vermont	2012	NHAsPI	106.20
Vermont	2009	Hispanic	105.80
Mississippi	2006	NHAIAN	105.40
Virginia	2012	Total	105.20
Florida	2012	NHWhite	104.70
Nevada	2007	NHBlack	104.70
Hawaii	2005	NHAIAN	104.30
Kentucky	2010	NHAIAN	104.20
Tennessee	2006	NHBlack	104.20
Hawaii	2006	Total	104.10
Louisiana	2018	NHWhite	103.90
Missouri	2007	Hispanic	103.80
Nevada	2009	NHWhite	103.80
Florida	2013	NHWhite	103.40
Florida	2014	NHWhite	103.30
Florida	2011	NHWhite	103.00
Vermont	2011	NHBlack	102.60
Michigan	2014	NHAsPI	102.40
Missouri	2011	NHAsPI	102.40
North Dakota	2011	Total	102.40
Vermont	2018	NHWhite	102.40
Nevada	2009	NHAIAN	100.80
Colorado	2007	Hispanic	100.70
Tennessee	2009	Hispanic	100.60
Florida	2010	NHWhite	100.20
Texas	2018	Hispanic	100.00
Nevada	2010	NHAIAN	99.10
Wisconsin	2013	NHAIAN	99.00
Vermont	2013	NHWhite	98.90
Nevada	2006	NHBlack	98.80
Vermont	2013	NHAsPI	98.60
Oklahoma	2007	NHAIAN	98.50
Vermont	2013	Total	98.40
West Virginia	2009	NHWhite	98.30
Virginia	2006	NHBlack	98.20
Virginia	2006	NHAIAN	97.60
Colorado	2007	NHAsPI	97.60
Virginia	2010	NHWhite	97.60
Colorado	2007	Total	97.20
Vermont	2014	NHWhite	97.20
Texas	2018	NHAIAN	96.90
Alaska	2005	NHWhite	96.40
Vermont	2014	Total	96.40
Colorado	2014	NHWhite	96.30
Maine	2013	Hispanic	96.30

Indiana	2010	NHAIAN	96.20
Virginia	2013	NHWhite	96.10
Mississippi	2011	NHAsPI	96.00
Nevada	2011	NHAIAN	96.00
Texas	2018	NHBlack	96.00
Michigan	2010	NHAIAN	95.80
Virginia	2009	NHWhite	95.60
West Virginia	2005	NHAIAN	95.20
Vermont	2018	Hispanic	95.20
Michigan	2012	Total	95.10
Indiana	2018	NHBlack	94.90
Colorado	2009	NHAIAN	94.80
Vermont	2012	Total	94.70
Louisiana	2018	Hispanic	94.40
Mississippi	2009	NHWhite	94.30
Oklahoma	2007	NHBlack	94.30
Vermont	2012	NHBlack	94.30
Louisiana	2006	NHBlack	94.20
Vermont	2012	NHWhite	94.00
Michigan	2007	Total	93.80
Texas	2014	Hispanic	93.50
Virginia	2009	NHAIAN	93.50
Tennessee	2007	Total	93.30
Michigan	2010	NHAsPI	93.20
Michigan	2011	Total	93.10
Virginia	2011	NHWhite	92.60
Virginia	2011	NHAIAN	92.40
Kentucky	2009	NHAIAN	92.30
Wisconsin	2010	NHAIAN	92.30
New York	2012	NHBlack	92.20
Virginia	2018	NHAsPI	92.00
Colorado	2007	NHWhite	91.90
New York	2014	Hispanic	91.90
Louisiana	2006	Hispanic	91.70
Michigan	2012	NHAsPI	91.60
Michigan	2011	NHAsPI	91.50
Indiana	2018	Hispanic	91.40
Virginia	2006	NHWhite	91.30
Minnesota	2018	NHAIAN	91.10
Wisconsin	2009	NHAIAN	90.90
Texas	2013	Hispanic	90.80
Kansas	2018	NHWhite	90.70
Texas	2014	NHBlack	90.50
Vermont	2013	NHBlack	90.30
Virginia	2007	NHBlack	90.30
Florida	2012	NHAsPI	90.10

Florida	2014	NHAsPI	90.10
Wisconsin	2012	NHAIAN	90.00
Florida	2011	NHAsPI	89.90
Texas	2018	Total	89.90
Michigan	2007	NHWhite	89.60
Tennessee	2013	NHAsPI	89.60
New York	2011	NHBlack	89.00
Colorado	2018	NHAsPI	88.90
New Jersey	2011	NHAIAN	88.70
West Virginia	2006	NHAIAN	88.50
Michigan	2007	NHAIAN	88.30
Wisconsin	2011	NHAIAN	88.30
Colorado	2014	NHAsPI	88.10
North Dakota	2010	Total	88.10
Texas	2013	NHBlack	88.10
Kentucky	2005	NHAsPI	88.10
Michigan	2006	Total	87.90
Texas	2012	NHBlack	87.70
Virginia	2012	NHWhite	87.40
Michigan	2010	Total	87.20
Missouri	2010	NHAsPI	87.10
Louisiana	2014	NHBlack	86.70
Texas	2014	Total	86.70
Florida	2010	NHAsPI	86.60
Texas	2012	Hispanic	86.60
Michigan	2009	Total	86.40
New York	2010	NHBlack	86.40
North Dakota	2011	NHWhite	86.30
Tennessee	2007	NHWhite	86.30
Florida	2013	NHAsPI	86.20
Maine	2011	NHAIAN	86.20
Virginia	2007	NHWhite	86.10
Oklahoma	2005	NHAIAN	86.00
North Dakota	2005	NHAIAN	85.90
Texas	2013	Total	85.50
Wisconsin	2006	NHAIAN	85.50
Missouri	2014	NHAIAN	85.40
Colorado	2005	NHAIAN	85.00
Colorado	2005	Hispanic	84.90
Michigan	2006	NHWhite	84.70
Kansas	2018	Total	84.70
Virginia	2012	NHAIAN	84.60
Texas	2011	NHBlack	84.20
West Virginia	2012	NHAsPI	84.20
North Dakota	2009	Total	84.10
Michigan	2009	NHAsPI	83.60

Virginia	2010	NHAIAN	83.60
Michigan	2011	NHWhite	83.50
Vermont	2014	NHAsPI	83.40
West Virginia	2014	NHAIAN	83.30
North Carolina	2011	NHAIAN	83.10
Ohio	2009	NHAIAN	82.90
Oklahoma	2009	NHAIAN	82.90
Texas	2012	Total	82.80
Michigan	2012	NHWhite	82.50
Ohio	2009	NHBlack	82.00
Oklahoma	2009	NHBlack	82.00
Texas	2011	Hispanic	81.70
Tennessee	2010	NHAsPI	81.60
Colorado	2005	Total	81.20
New Jersey	2007	NHAIAN	81.20
Michigan	2009	NHWhite	81.10
New Jersey	2018	NHBlack	80.50
Michigan	2010	NHWhite	80.40
Oklahoma	2007	NHWhite	80.40
Texas	2013	NHWhite	80.40
Kansas	2018	NHBlack	80.20
Texas	2014	NHWhite	80.00
Wisconsin	2007	NHAIAN	80.00
North Dakota	2011	NHAsPI	79.90
Texas	2018	NHWhite	79.90
Vermont	2011	Total	79.60
West Virginia	2011	NHAsPI	79.60
Wisconsin	2014	NHAIAN	79.40
Texas	2010	NHBlack	79.30
Texas	2011	Total	79.30
Vermont	2009	NHBlack	79.20
Vermont	2011	NHWhite	78.90
Tennessee	2005	NHBlack	78.70
Arkansas	2010	NHAsPI	78.70
Tennessee	2006	Total	78.70
Michigan	2012	Hispanic	78.60
Tennessee	2011	NHAsPI	78.50
Texas	2012	NHWhite	78.40
North Carolina	2009	NHAIAN	78.30
Tennessee	2012	NHAsPI	78.10
New Jersey	2014	NHBlack	77.70
Arkansas	2009	NHAsPI	77.60
New York	2009	NHBlack	77.50
Colorado	2005	NHWhite	77.40
Texas	2010	Hispanic	77.20
Tennessee	2009	NHAsPI	77.00

Missouri	2013	NHAIAN	76.90
Rhode Island	2010	NHAIAN	76.90
Indiana	2018	Total	76.70
New York	2013	Hispanic	76.70
New York	2014	Total	76.50
West Virginia	2006	Total	76.30
Texas	2011	NHWhite	76.30
Mississippi	2012	NHAsPI	76.20
Oklahoma	2010	NHBlack	76.20
Rhode Island	2006	NHAIAN	76.10
North Carolina	2010	NHAIAN	76.10
New York	2006	NHBlack	75.80
West Virginia	2007	Total	75.80
Michigan	2006	Hispanic	75.50
North Carolina	2018	NHAIAN	75.10
Texas	2010	Total	74.90
North Dakota	2006	NHAsPI	74.80
Mississippi	2010	NHAsPI	74.50
West Virginia	2006	NHWhite	74.40
Tennessee	2006	NHWhite	74.30
North Dakota	2005	NHAsPI	74.20
North Carolina	2012	NHAIAN	74.20
Oklahoma	2006	NHAIAN	74.20
Hawaii	2007	NHAIAN	74.10
Indiana	2014	NHAIAN	74.10
Indiana	2018	NHWhite	73.90
Michigan	2007	NHAsPI	73.70
West Virginia	2012	Hispanic	73.70
Michigan	2006	NHAIAN	73.40
Mississippi	2005	NHAIAN	73.10
Colorado	2007	NHAIAN	73.10
New Jersey	2013	NHBlack	72.90
Louisiana	2013	NHBlack	72.60
Connecticut	2007	NHBlack	72.50
New York	2007	NHBlack	72.40
Oklahoma	2005	NHBlack	72.30
Louisiana	2007	NHAIAN	72.30
New York	2005	NHBlack	72.30
North Dakota	2009	NHWhite	72.30
Texas	2011	NHAIAN	72.30
North Dakota	2010	NHWhite	72.10
Mississippi	2009	NHAsPI	72.00
Louisiana	2014	Total	71.90
Ohio	2009	NHWhite	71.90
Oklahoma	2009	NHWhite	71.90
Texas	2009	NHBlack	71.70

Texas	2010	NHWhite	71.70
Louisiana	2006	NHAsPI	71.30
Tennessee	2007	Hispanic	71.00
New Jersey	2012	NHAIAN	70.90
Rhode Island	2007	NHAIAN	70.80
Louisiana	2013	NHAIAN	70.50
Indiana	2018	NHAIAN	70.40
North Dakota	2007	Total	70.20
Oklahoma	2010	NHWhite	70.00
Colorado	2005	NHAsPI	69.90
Ohio	2009	Total	69.80
Oklahoma	2009	Total	69.80
Maine	2014	Hispanic	69.70
Michigan	2010	Hispanic	69.70
New York	2013	Total	69.60
New York	2014	NHAsPI	69.60
Oklahoma	2010	NHAIAN	69.60
Texas	2007	NHBlack	69.30
Nevada	2007	NHAsPI	69.10
Nevada	2006	Hispanic	69.00
Nevada	2007	Total	68.90
North Carolina	2013	NHAIAN	68.90
New Jersey	2018	NHAIAN	68.70
West Virginia	2018	NHAsPI	68.70
Oklahoma	2005	NHWhite	68.60
Texas	2006	NHAIAN	68.50
New York	2012	Hispanic	68.40
Texas	2014	NHAIAN	68.40
Nevada	2007	Hispanic	68.00
Oklahoma	2007	Total	68.00
Nevada	2006	Total	67.80
Hawaii	2007	NHWhite	67.80
Kansas	2018	Hispanic	67.70
Michigan	2011	Hispanic	67.60
Vermont	2010	Total	67.60
Texas	2012	NHAsPI	67.50
Louisiana	2018	NHAsPI	67.30
Oklahoma	2011	NHBlack	67.30
West Virginia	2005	NHWhite	67.30
Utah	2005	NHBlack	67.20
Nevada	2006	NHAsPI	67.10
New York	2012	NHAsPI	67.10
North Dakota	2010	NHAsPI	67.10
Oklahoma	2005	Total	67.00
Mississippi	2011	Hispanic	66.90
Oklahoma	2010	Total	66.70

Texas	2007	NHAIAN	66.70
West Virginia	2005	Total	66.30
Vermont	2010	NHWhite	66.30
West Virginia	2011	Hispanic	66.20
West Virginia	2007	NHWhite	66.10
Georgia	2007	NHAIAN	66.00
North Carolina	2011	NHBlack	66.00
Texas	2009	Hispanic	66.00
Oklahoma	2006	NHBlack	65.80
Virginia	2007	NHAIAN	65.80
Rhode Island	2005	NHAIAN	65.80
North Dakota	2006	Total	65.70
Texas	2013	NHAsPI	65.70
Michigan	2009	Hispanic	65.60
Oklahoma	2011	NHWhite	65.60
Texas	2009	Total	65.60
North Dakota	2005	Total	65.30
Texas	2006	NHBlack	65.20
New York	2011	NHAsPI	65.20
Texas	2007	Hispanic	65.20
Texas	2014	NHAsPI	65.00
West Virginia	2009	NHBlack	65.00
Rhode Island	2009	NHAIAN	64.90
Texas	2011	NHAsPI	64.80
Vermont	2011	NHAsPI	64.80
Texas	2007	Total	64.70
Texas	2009	NHWhite	64.60
Indiana	2018	NHAsPI	64.40
Texas	2009	NHAIAN	64.40
New York	2012	Total	64.30
Texas	2007	NHWhite	64.30
New Jersey	2012	NHBlack	64.20
North Carolina	2010	NHBlack	64.10
Texas	2012	NHAIAN	64.10
Texas	2006	Hispanic	64.00
North Dakota	2009	NHAsPI	63.50
Oklahoma	2011	NHAIAN	63.50
Louisiana	2013	Total	63.20
North Carolina	2012	NHBlack	63.20
North Carolina	2014	NHAIAN	63.20
New York	2013	NHAsPI	63.00
New York	2011	Hispanic	62.90
Louisiana	2014	NHWhite	62.70
Mississippi	2007	NHBlack	62.70
Texas	2006	Total	62.50
Tennessee	2005	Total	62.50

Rhode Island	2014	NHAIAN	62.30
Oklahoma	2011	Total	62.20
Oklahoma	2006	NHWhite	62.00
Nevada	2007	NHWhite	61.90
New York	2010	Hispanic	61.80
Rhode Island	2009	NHBlack	61.80
North Carolina	2007	NHAIAN	61.40
Texas	2006	NHWhite	61.30
Louisiana	2010	NHAIAN	61.20
North Carolina	2009	NHBlack	61.20
Utah	2006	NHBlack	61.20
Nevada	2006	NHWhite	61.10
Texas	2005	NHBlack	60.80
Connecticut	2005	NHBlack	60.60
Louisiana	2014	Hispanic	60.60
North Dakota	2007	NHWhite	60.60
Tennessee	2005	NHWhite	60.50
New Jersey	2013	NHAIAN	60.50
Tennessee	2006	Hispanic	60.50
Indiana	2010	NHAsPI	60.40
North Carolina	2013	NHBlack	60.30
Louisiana	2009	NHBlack	60.20
Louisiana	2013	Hispanic	60.10
Texas	2010	NHAsPI	60.10
Louisiana	2012	NHBlack	59.90
Indiana	2014	NHBlack	59.60
Texas	2005	Hispanic	59.50
West Virginia	2006	NHAsPI	59.50
Louisiana	2007	NHAsPI	59.40
Mississippi	2010	Hispanic	59.40
Oklahoma	2006	Total	59.40
New York	2014	NHWhite	59.00
Oklahoma	2012	NHWhite	59.00
Delaware	2018	NHAIAN	58.80
Iowa	2007	NHAIAN	58.70
Texas	2010	NHAIAN	58.70
Texas	2013	NHAIAN	58.50
Tennessee	2007	NHAsPI	58.40
North Carolina	2005	NHAIAN	58.40
Arkansas	2011	NHAsPI	58.30
Texas	2005	Total	58.30
Hawaii	2006	NHAIAN	58.20
North Carolina	2014	NHBlack	58.10
Texas	2018	NHAsPI	58.10
Michigan	2006	NHAsPI	57.90
Oklahoma	2005	Hispanic	57.80

Utah	2007	NHBlack	57.70
New Jersey	2006	NHBlack	57.40
Louisiana	2009	Total	57.30
North Dakota	2005	NHWhite	57.10
Louisiana	2013	NHWhite	57.10
Rhode Island	2013	NHAIAN	57.10
Georgia	2006	NHAIAN	57.00
North Dakota	2006	NHWhite	56.90
Hawaii	2005	NHWhite	56.70
Oklahoma	2012	NHBlack	56.70
Louisiana	2007	NHWhite	56.50
North Carolina	2011	Total	56.50
Oklahoma	2012	Total	56.50
Texas	2005	NHWhite	56.50
Iowa	2006	NHAIAN	56.40
North Carolina	2012	Total	56.30
Louisiana	2009	NHWhite	56.20
Indiana	2010	NHBlack	56.00
Utah	2011	NHAsPI	56.00
West Virginia	2010	Hispanic	56.00
New Jersey	2005	NHBlack	55.70
New York	2011	Total	55.50
North Carolina	2018	NHBlack	55.40
Utah	2009	NHAsPI	55.40
Utah	2010	NHAsPI	55.40
Louisiana	2007	Total	55.10
Michigan	2007	Hispanic	55.10
North Carolina	2006	NHAIAN	55.00
North Carolina	2012	NHWhite	54.70
Oklahoma	2012	NHAIAN	54.60
Louisiana	2012	Total	54.50
Vermont	2009	Total	54.40
North Carolina	2013	Total	54.30
West Virginia	2013	Hispanic	54.10
New York	2009	Hispanic	54.00
North Carolina	2010	Total	54.00
Arkansas	2014	NHAsPI	53.80
Louisiana	2012	Hispanic	53.80
North Carolina	2011	NHWhite	53.80
Louisiana	2007	NHBlack	53.70
Indiana	2014	Hispanic	53.60
North Carolina	2013	NHWhite	53.50
Vermont	2009	NHWhite	53.30
Louisiana	2012	NHAIAN	53.20
North Carolina	2012	Hispanic	53.00
Wisconsin	2013	NHWhite	52.90

Wisconsin	2014	NHWhite	52.90
Wisconsin	2014	NHBlack	52.70
Connecticut	2007	NHAsPI	52.50
Wisconsin	2013	Total	52.40
Wisconsin	2014	Total	52.40
Mississippi	2007	Total	52.30
New Jersey	2011	NHBlack	52.30
Iowa	2009	NHAIAN	52.20
New York	2010	Total	52.20
North Carolina	2011	Hispanic	52.20
Indiana	2010	Total	52.00
Louisiana	2014	NHAsPI	52.00
New Jersey	2007	NHBlack	51.90
Wisconsin	2013	NHBlack	51.90
North Carolina	2009	Total	51.70
Rhode Island	2005	NHBlack	51.60
New York	2010	NHAsPI	51.60
New York	2013	NHWhite	51.60
North Carolina	2007	NHBlack	51.60
Rhode Island	2007	NHBlack	51.60
New Jersey	2009	NHBlack	51.50
Indiana	2010	NHWhite	51.30
Nevada	2007	NHAIAN	51.30
North Carolina	2010	Hispanic	51.30
Vermont	2010	NHAsPI	51.30
Mississippi	2012	Hispanic	51.10
North Carolina	2014	Total	51.10
Louisiana	2007	Hispanic	51.00
Wisconsin	2012	NHWhite	51.00
Connecticut	2005	NHAsPI	50.90
Louisiana	2012	NHWhite	50.90
Connecticut	2007	Hispanic	50.70
North Carolina	2010	NHWhite	50.70
Texas	2009	NHAsPI	50.70
Connecticut	2007	Total	50.60
Wisconsin	2012	Total	50.60
Wisconsin	2014	Hispanic	50.40
Oklahoma	2013	NHWhite	50.30
Hawaii	2006	NHWhite	50.10
North Carolina	2014	NHWhite	50.00
Indiana	2014	Total	49.90
Utah	2018	NHAIAN	49.90
Wisconsin	2012	Hispanic	49.90
Tennessee	2012	NHAIAN	49.80
North Carolina	2013	Hispanic	49.60
Oklahoma	2013	Total	49.50

Wisconsin	2013	NHAsPI	49.50
New Jersey	2010	NHBlack	49.40
Indiana	2014	NHWhite	49.30
Maine	2013	NHAIAN	49.30
North Carolina	2009	Hispanic	49.30
Wisconsin	2010	Hispanic	49.30
Georgia	2005	NHAIAN	49.30
Iowa	2010	NHBlack	49.00
Tennessee	2006	NHAsPI	48.80
New York	2005	NHAsPI	48.60
North Carolina	2018	Total	48.50
Wisconsin	2011	NHWhite	48.50
Texas	2005	NHAIAN	48.50
New York	2009	NHAsPI	48.40
North Carolina	2009	NHWhite	48.40
North Carolina	2005	NHBlack	48.40
West Virginia	2005	NHBlack	48.30
Texas	2007	NHAsPI	48.20
Wisconsin	2011	Total	48.20
Utah	2011	NHBlack	48.10
New York	2006	NHAsPI	48.00
Arkansas	2012	NHAsPI	47.90
New York	2009	Total	47.90
Wisconsin	2011	Hispanic	47.80
North Carolina	2018	Hispanic	47.50
Wisconsin	2012	NHBlack	47.40
Iowa	2009	NHBlack	47.30
Oklahoma	2010	Hispanic	47.30
Utah	2007	NHAIAN	47.30
New Jersey	2014	Hispanic	47.20
Rhode Island	2010	NHBlack	47.20
Wisconsin	2013	Hispanic	47.20
North Carolina	2006	NHBlack	47.10
Oklahoma	2011	Hispanic	47.00
Wisconsin	2014	NHAsPI	46.90
Oklahoma	2006	Hispanic	46.80
Connecticut	2007	NHWhite	46.80
New Jersey	2006	NHAIAN	46.70
Illinois	2011	NHAIAN	46.60
Utah	2011	Hispanic	46.60
Mississippi	2009	Hispanic	46.50
Oklahoma	2013	NHBlack	46.50
Utah	2009	Hispanic	46.50
Wisconsin	2011	NHBlack	46.50
Louisiana	2010	NHBlack	46.40
New Jersey	2018	Hispanic	46.40

Rhode Island	2011	NHAIAN	46.40
Utah	2010	Hispanic	46.40
Wisconsin	2010	NHWhite	46.40
Wisconsin	2010	Total	46.40
North Carolina	2018	NHWhite	46.30
North Carolina	2014	Hispanic	46.20
Mississippi	2006	NHBlack	46.10
Oklahoma	2007	Hispanic	46.00
Oklahoma	2014	NHWhite	46.00
New York	2006	Total	46.00
Oklahoma	2014	Total	45.90
Indiana	2010	Hispanic	45.80
New York	2007	Total	45.80
Utah	2006	NHAIAN	45.80
Iowa	2012	NHBlack	45.70
New York	2007	NHAsPI	45.70
Ohio	2009	Hispanic	45.70
Oklahoma	2009	Hispanic	45.70
Texas	2006	NHAsPI	45.70
Illinois	2018	NHBlack	45.60
Louisiana	2009	Hispanic	45.60
Utah	2012	Hispanic	45.60
New York	2005	Total	45.60
Tennessee	2005	Hispanic	45.50
New York	2007	Hispanic	45.50
Utah	2007	NHAsPI	45.40
New York	2005	Hispanic	45.30
Louisiana	2010	Total	45.20
Massachusetts	2018	NHAIAN	45.20
Oklahoma	2012	Hispanic	45.20
Rhode Island	2006	NHBlack	45.20
Rhode Island	2012	NHAIAN	45.10
Connecticut	2005	Total	45.10
Delaware	2014	Hispanic	45.00
Arkansas	2007	NHAIAN	44.90
Oklahoma	2013	NHAIAN	44.80
Louisiana	2013	NHAsPI	44.60
Louisiana	2010	NHWhite	44.50
Illinois	2012	NHAIAN	44.40
New York	2012	NHWhite	44.30
Wisconsin	2012	NHAsPI	44.30
New York	2006	Hispanic	44.10
Louisiana	2010	Hispanic	44.10
Utah	2013	NHAIAN	44.10
Iowa	2011	NHBlack	43.90
Utah	2013	Hispanic	43.80

Utah	2009	NHAIAN	43.70
New Hampshire	2010	NHAIAN	43.50
New Jersey	2013	Hispanic	43.50
Iowa	2010	NHAIAN	43.40
Mississippi	2007	NHAsPI	43.30
New York	2007	NHAIAN	43.30
Utah	2010	NHAIAN	43.30
Wisconsin	2009	NHWhite	43.30
Connecticut	2005	Hispanic	43.20
Delaware	2013	Hispanic	43.10
Utah	2009	Total	43.10
Utah	2010	Total	42.90
North Carolina	2012	NHAsPI	42.70
Utah	2011	Total	42.70
Connecticut	2005	NHWhite	42.60
Wisconsin	2009	Total	42.60
Delaware	2012	Hispanic	42.40
Utah	2009	NHWhite	42.30
Rhode Island	2011	NHBlack	42.20
Utah	2010	NHWhite	42.10
Utah	2011	NHAIAN	42.10
Mississippi	2007	NHWhite	42.00
Nevada	2006	NHAIAN	41.80
North Carolina	2013	NHAsPI	41.80
Oklahoma	2014	NHBlack	41.80
Rhode Island	2006	Hispanic	41.70
Iowa	2007	NHBlack	41.70
Iowa	2013	NHBlack	41.70
Kansas	2018	NHAsPI	41.70
Utah	2011	NHWhite	41.70
Utah	2014	NHAIAN	41.70
Louisiana	2010	NHAsPI	41.60
Oklahoma	2013	Hispanic	41.60
Delaware	2018	NHBlack	41.50
Utah	2012	NHAsPI	41.50
Wisconsin	2010	NHBlack	41.50
Utah	2009	NHBlack	41.30
Oklahoma	2007	NHAsPI	41.20
Utah	2012	Total	41.20
North Carolina	2011	NHAsPI	41.10
West Virginia	2006	NHBlack	41.10
Illinois	2013	Hispanic	41.00
Indiana	2014	NHAsPI	41.00
Maine	2011	NHAsPI	41.00
Iowa	2006	NHBlack	40.90
Utah	2005	NHAIAN	40.80

Wisconsin	2009	Hispanic	40.70
Delaware	2018	Hispanic	40.60
New Jersey	2014	Total	40.60
North Carolina	2007	Total	40.60
Rhode Island	2013	NHBlack	40.60
Utah	2013	NHAsPI	40.60
Iowa	2014	NHBlack	40.50
Utah	2012	NHWhite	40.40
Illinois	2018	NHAsPI	40.30
Oklahoma	2014	NHAIAN	40.30
Utah	2014	Hispanic	40.20
Delaware	2011	Hispanic	40.10
Oklahoma	2014	Hispanic	40.00
New Jersey	2006	Hispanic	39.90
Arkansas	2018	NHWhite	39.70
Utah	2007	Hispanic	39.60
Oklahoma	2014	NHAsPI	39.50
Rhode Island	2007	Hispanic	39.50
West Virginia	2009	Hispanic	39.50
Utah	2006	Hispanic	39.40
New York	2009	NHAIAN	39.40
New Jersey	2005	Hispanic	39.30
Utah	2013	Total	39.20
Arkansas	2014	NHBlack	39.10
Wisconsin	2011	NHAsPI	39.10
New Jersey	2018	Total	39.00
Utah	2006	NHAsPI	38.90
Arkansas	2011	Total	38.80
Illinois	2014	NHAsPI	38.80
Iowa	2018	NHAIAN	38.80
Minnesota	2014	NHAIAN	38.80
New York	2011	NHWhite	38.70
North Carolina	2014	NHAsPI	38.70
Utah	2013	NHWhite	38.70
Arkansas	2009	NHAIAN	38.60
Utah	2013	NHBlack	38.50
New Jersey	2012	Hispanic	38.30
Arkansas	2011	Hispanic	38.10
Utah	2014	NHAsPI	38.10
Utah	2005	Hispanic	38.00
Minnesota	2013	NHAIAN	37.90
Rhode Island	2007	Total	37.90
Georgia	2005	Hispanic	37.90
Utah	2005	NHAsPI	37.90
Arkansas	2012	Hispanic	37.70
Illinois	2018	Hispanic	37.70

North Carolina	2005	Total	37.70
Rhode Island	2007	NHWhite	37.70
Utah	2007	Total	37.70
Mississippi	2006	Total	37.70
North Carolina	2006	Total	37.60
North Carolina	2007	NHWhite	37.60
Wisconsin	2007	NHBlack	37.60
Wisconsin	2009	NHBlack	37.60
Indiana	2007	NHAsPI	37.50
New Hampshire	2007	Hispanic	37.50
Utah	2010	NHBlack	37.50
Arkansas	2018	NHBlack	37.40
Illinois	2018	Total	37.40
North Carolina	2009	NHAsPI	37.40
Tennessee	2005	NHAIAN	37.40
Iowa	2005	NHAIAN	37.30
Maine	2012	NHAIAN	37.30
Maine	2018	NHBlack	37.30
New Jersey	2013	Total	37.30
New York	2010	NHWhite	37.30
North Carolina	2010	NHAsPI	37.30
Tennessee	2005	NHAsPI	37.30
Georgia	2005	NHBlack	37.30
Wisconsin	2006	NHBlack	37.30
New Jersey	2007	Hispanic	37.20
Rhode Island	2009	Total	37.20
Maine	2013	NHBlack	37.10
Wisconsin	2007	NHWhite	37.10
Georgia	2005	NHWhite	37.10
Georgia	2005	Total	37.00
New York	2007	NHWhite	37.00
Wisconsin	2007	Total	37.00
Maine	2014	NHAIAN	36.90
Utah	2007	NHWhite	36.90
Indiana	2005	NHWhite	36.80
Massachusetts	2007	NHAIAN	36.80
Arkansas	2018	Total	36.70
Delaware	2018	Total	36.70
Massachusetts	2014	Hispanic	36.70
Utah	2018	Hispanic	36.70
West Virginia	2005	NHAsPI	36.70
Arkansas	2010	NHAIAN	36.60
Arkansas	2014	Hispanic	36.60
Arkansas	2014	Total	36.60
Oklahoma	2012	NHAsPI	36.60
New York	2006	NHWhite	36.50

Wisconsin	2010	NHAsPI	36.40
Iowa	2005	NHBlack	36.40
Oklahoma	2005	NHAsPI	36.40
Rhode Island	2009	Hispanic	36.30
Indiana	2007	NHWhite	36.20
Utah	2012	NHBlack	36.20
New York	2005	NHWhite	36.10
Illinois	2018	NHWhite	36.00
Utah	2006	Total	35.90
Arkansas	2012	NHBlack	35.90
Rhode Island	2009	NHWhite	35.90
Arkansas	2018	NHAsPI	35.60
Iowa	2010	Hispanic	35.60
Louisiana	2011	NHBlack	35.60
Georgia	2006	NHWhite	35.60
Louisiana	2011	Hispanic	35.50
New York	2009	NHWhite	35.50
Arkansas	2010	NHBlack	35.40
New York	2014	NHAIAN	35.40
Rhode Island	2005	Hispanic	35.40
Rhode Island	2006	Total	35.40
Arkansas	2009	NHWhite	35.30
New Jersey	2009	Hispanic	35.30
North Dakota	2007	NHAsPI	35.30
Oklahoma	2006	NHAsPI	35.30
Arkansas	2009	Total	35.20
Illinois	2014	NHBlack	35.20
New York	2010	NHAIAN	35.20
North Carolina	2007	Hispanic	35.20
Utah	2014	Total	35.20
West Virginia	2014	Hispanic	35.20
Arkansas	2011	NHBlack	35.10
North Carolina	2006	NHWhite	35.10
Arkansas	2012	NHWhite	34.90
West Virginia	2013	NHAsPI	34.90
West Virginia	2014	NHAsPI	34.90
Utah	2006	NHWhite	34.80
Arkansas	2010	Total	34.70
Arkansas	2011	NHWhite	34.70
North Carolina	2018	NHAsPI	34.70
Rhode Island	2005	Total	34.60
West Virginia	2007	NHAsPI	34.60
New York	2006	NHAIAN	34.50
Arkansas	2005	Hispanic	34.50
Illinois	2014	Hispanic	34.50
Oklahoma	2013	NHAsPI	34.50

Wisconsin	2007	Hispanic	34.50
North Carolina	2005	NHWhite	34.50
Arkansas	2010	NHWhite	34.40
Louisiana	2011	Total	34.40
Louisiana	2012	NHAsPI	34.40
Indiana	2005	Total	34.30
Tennessee	2010	NHAIAN	34.20
Indiana	2007	Total	34.10
Mississippi	2005	NHBlack	34.00
Arkansas	2014	NHWhite	33.90
Louisiana	2011	NHWhite	33.90
Wisconsin	2006	Total	33.90
Massachusetts	2018	NHBlack	33.80
West Virginia	2007	NHBlack	33.80
West Virginia	2005	Hispanic	33.80
Maine	2011	Total	33.70
New Jersey	2011	Hispanic	33.70
Utah	2005	Total	33.70
Utah	2014	NHWhite	33.70
New Jersey	2012	Total	33.60
New Jersey	2005	Total	33.60
Maine	2014	NHBlack	33.50
Rhode Island	2005	NHWhite	33.50
Delaware	2018	NHAsPI	33.30
Iowa	2018	NHBlack	33.30
New Jersey	2006	Total	33.30
North Carolina	2006	Hispanic	33.10
Arkansas	2007	NHAsPI	33.10
Arkansas	2009	NHBlack	33.10
Maryland	2018	NHAIAN	33.10
Wisconsin	2006	NHWhite	33.00
Arkansas	2009	Hispanic	33.00
Delaware	2018	NHWhite	33.00
Utah	2018	NHAsPI	33.00
North Carolina	2005	Hispanic	32.90
North Carolina	2005	NHAsPI	32.80
Illinois	2013	NHBlack	32.70
Rhode Island	2010	Hispanic	32.60
Wisconsin	2006	Hispanic	32.50
Georgia	2006	Total	32.40
Utah	2005	NHWhite	32.30
Delaware	2014	Total	32.30
Illinois	2014	Total	32.30
Maine	2011	NHWhite	32.30
Iowa	2006	NHWhite	32.20
Utah	2018	Total	32.20

Rhode Island	2010	Total	32.10
New Jersey	2007	Total	32.00
New Jersey	2010	Hispanic	32.00
Iowa	2012	NHWhite	31.90
Iowa	2012	Total	31.90
Georgia	2006	NHBlack	31.90
Illinois	2013	NHAsPI	31.80
Rhode Island	2006	NHWhite	31.80
Arkansas	2005	NHWhite	31.80
Indiana	2006	NHWhite	31.70
Iowa	2006	Total	31.70
Iowa	2005	NHWhite	31.70
Georgia	2006	Hispanic	31.50
Rhode Island	2012	NHBlack	31.50
Indiana	2012	NHBlack	31.30
Illinois	2006	NHBlack	31.20
Iowa	2011	Hispanic	31.20
Iowa	2011	Total	31.20
Iowa	2013	NHWhite	31.20
Nebraska	2009	NHAsPI	31.10
Iowa	2005	Total	31.00
Maine	2013	Total	31.00
Iowa	2007	NHWhite	30.90
Iowa	2009	Total	30.90
Iowa	2011	NHWhite	30.90
Iowa	2013	Hispanic	30.90
Iowa	2013	Total	30.90
Minnesota	2018	Hispanic	30.90
Utah	2018	NHWhite	30.90
Arkansas	2005	Total	30.80
Illinois	2014	NHWhite	30.80
Illinois	2005	NHBlack	30.80
New Jersey	2005	NHAIAN	30.70
Tennessee	2006	NHAIAN	30.70
New Jersey	2009	Total	30.70
North Carolina	2007	NHAsPI	30.70
New Jersey	2005	NHAsPI	30.60
Georgia	2007	NHWhite	30.60
Indiana	2007	NHBlack	30.60
Iowa	2009	NHWhite	30.60
Arkansas	2010	Hispanic	30.50
Delaware	2013	Total	30.50
Delaware	2014	NHBlack	30.50
Iowa	2007	Total	30.50
Indiana	2006	NHAsPI	30.50
Iowa	2012	Hispanic	30.40

Iowa	2010	Total	30.30
Arkansas	2014	NHAIAN	30.20
Nebraska	2007	NHBlack	30.20
Rhode Island	2010	NHWhite	30.20
Indiana	2006	Total	30.10
Illinois	2013	Total	30.10
Indiana	2013	NHBlack	30.10
Iowa	2009	Hispanic	30.10
Kansas	2014	NHBlack	30.10
Minnesota	2012	NHAIAN	30.10
Minnesota	2018	NHBlack	30.10
New Jersey	2014	NHAsPI	30.10
Utah	2014	NHBlack	30.10
New Hampshire	2006	Hispanic	30.10
Mississippi	2006	NHWhite	30.00
Delaware	2014	NHWhite	30.00
Illinois	2012	NHAsPI	30.00
Massachusetts	2014	NHBlack	30.00
New York	2013	NHAIAN	30.00
Oklahoma	2011	NHAsPI	30.00
Rhode Island	2018	Hispanic	30.00
Wisconsin	2009	NHAsPI	29.90
Illinois	2012	NHBlack	29.80
Massachusetts	2010	NHBlack	29.80
Delaware	2012	Total	29.70
Massachusetts	2018	Hispanic	29.70
New Jersey	2011	Total	29.70
Delaware	2012	NHWhite	29.60
New York	2005	NHAIAN	29.50
Arkansas	2007	NHWhite	29.50
Iowa	2014	NHWhite	29.50
Illinois	2012	Hispanic	29.40
Iowa	2014	Total	29.40
Kansas	2011	NHBlack	29.40
Delaware	2009	Hispanic	29.30
Illinois	2005	Total	29.30
Delaware	2013	NHWhite	29.20
Illinois	2007	NHBlack	29.20
Rhode Island	2014	NHBlack	29.20
Iowa	2010	NHWhite	29.10
West Virginia	2006	Hispanic	29.10
Illinois	2011	NHBlack	29.00
Maine	2012	Total	29.00
New Jersey	2010	Total	29.00
North Carolina	2006	NHAsPI	28.90
Georgia	2018	NHBlack	28.90

Utah	2012	NHAIAN	28.90
South Carolina	2007	Hispanic	28.80
New Jersey	2006	NHAsPI	28.70
Maine	2013	NHWhite	28.60
Massachusetts	2011	NHBlack	28.60
New Jersey	2013	NHAsPI	28.60
Maine	2014	Total	28.40
Delaware	2010	Hispanic	28.30
Delaware	2011	NHWhite	28.10
Illinois	2010	NHBlack	28.10
Kansas	2014	Hispanic	28.10
Indiana	2005	NHBlack	28.00
Arkansas	2007	Total	28.00
New Jersey	2014	NHWhite	28.00
Ohio	2009	NHAsPI	28.00
Oklahoma	2009	NHAsPI	28.00
Illinois	2009	NHBlack	27.90
Delaware	2010	NHWhite	27.80
Illinois	2012	Total	27.80
Minnesota	2018	Total	27.80
Utah	2018	NHBlack	27.80
Delaware	2014	NHAsPI	27.70
Illinois	2011	Hispanic	27.70
Georgia	2007	Total	27.50
New York	2011	NHAIAN	27.50
North Dakota	2006	Hispanic	27.40
Illinois	2012	NHWhite	27.30
Maine	2012	NHWhite	27.30
Rhode Island	2011	Total	27.30
California	2018	NHBlack	27.20
Illinois	2011	NHAsPI	27.20
Massachusetts	2013	NHBlack	27.20
Massachusetts	2018	Total	27.20
New Jersey	2018	NHAsPI	27.20
Georgia	2007	NHBlack	27.10
Arkansas	2005	NHBlack	27.00
Delaware	2011	Total	27.00
Delaware	2012	NHAsPI	27.00
Delaware	2013	NHBlack	27.00
Massachusetts	2012	NHBlack	27.00
New Jersey	2007	NHAsPI	27.00
Illinois	2006	Total	27.00
Iowa	2018	NHAsPI	26.90
Illinois	2006	NHWhite	26.80
Georgia	2005	NHAsPI	26.80
Illinois	2013	NHWhite	26.80

Delaware	2007	Hispanic	26.70
Delaware	2009	NHWhite	26.70
Illinois	2011	Total	26.70
New Jersey	2018	NHWhite	26.70
Illinois	2005	NHWhite	26.60
Illinois	2005	Hispanic	26.60
Maine	2014	NHWhite	26.60
Massachusetts	2009	NHBlack	26.60
Nebraska	2009	Total	26.60
Massachusetts	2006	NHAIAN	26.50
Iowa	2005	Hispanic	26.50
Iowa	2010	NHAsPI	26.50
Rhode Island	2011	NHWhite	26.50
Indiana	2013	NHWhite	26.40
Kansas	2010	NHBlack	26.40
Massachusetts	2014	Total	26.40
Nebraska	2009	Hispanic	26.40
Georgia	2018	Hispanic	26.30
Massachusetts	2014	NHWhite	26.20
Minnesota	2018	NHWhite	26.20
Georgia	2007	Hispanic	26.10
Indiana	2013	Total	26.10
New Jersey	2005	NHWhite	26.10
Indiana	2005	NHAsPI	26.00
Nebraska	2009	NHWhite	26.00
New Hampshire	2009	NHBlack	26.00
Ohio	2013	NHAIAN	26.00
Massachusetts	2018	NHWhite	25.90
Rhode Island	2018	Total	25.90
Georgia	2018	Total	25.80
Illinois	2011	NHWhite	25.80
Indiana	2012	Total	25.80
Iowa	2006	Hispanic	25.80
Massachusetts	2005	NHAIAN	25.70
Minnesota	2007	NHBlack	25.70
Delaware	2013	NHAsPI	25.60
Indiana	2012	NHWhite	25.60
Minnesota	2013	NHAsPI	25.60
Kansas	2010	Hispanic	25.50
Minnesota	2014	Hispanic	25.40
Nebraska	2009	NHBlack	25.30
North Dakota	2010	Hispanic	25.30
Massachusetts	2005	NHBlack	25.20
Wisconsin	2007	NHAsPI	25.20
Mississippi	2005	Total	25.00
Indiana	2007	Hispanic	25.00

Louisiana	2011	NHAsPI	25.00
New Jersey	2006	NHWhite	25.00
Massachusetts	2006	NHBlack	24.90
Indiana	2012	Hispanic	24.80
Indiana	2006	NHBlack	24.80
Wisconsin	2006	NHAsPI	24.70
Illinois	2010	Total	24.70
South Carolina	2007	NHBlack	24.70
Massachusetts	2013	Hispanic	24.60
New Jersey	2007	NHWhite	24.60
Illinois	2007	Total	24.50
Illinois	2010	Hispanic	24.50
New Jersey	2009	NHAsPI	24.50
Indiana	2005	Hispanic	24.40
Illinois	2010	NHWhite	24.40
New Jersey	2012	NHAsPI	24.40
Oklahoma	2018	NHBlack	24.40
Rhode Island	2012	Total	24.40
Arkansas	2007	Hispanic	24.30
Illinois	2007	NHWhite	24.30
California	2018	NHWhite	24.20
Delaware	2009	Total	24.20
Delaware	2010	Total	24.20
Indiana	2013	NHAIAN	24.20
Iowa	2014	Hispanic	24.20
Massachusetts	2007	NHBlack	24.20
Massachusetts	2013	Total	24.20
Minnesota	2013	NHBlack	24.20
Rhode Island	2012	NHWhite	24.20
Illinois	2006	Hispanic	24.10
North Dakota	2005	Hispanic	24.10
Delaware	2012	NHBlack	24.00
Indiana	2007	NHAIAN	24.00
Iowa	2014	NHAsPI	24.00
Massachusetts	2013	NHWhite	24.00
New Jersey	2013	NHWhite	24.00
Oklahoma	2018	NHWhite	24.00
Arkansas	2007	NHBlack	23.90
Delaware	2007	NHWhite	23.90
Oklahoma	2018	Hispanic	23.90
South Carolina	2010	Hispanic	23.90
Oklahoma	2010	NHAsPI	23.80
South Carolina	2007	Total	23.80
Georgia	2014	NHBlack	23.70
Georgia	2018	NHWhite	23.70
Illinois	2009	Total	23.70

Nebraska	2010	NHAsPI	23.70
Illinois	2009	NHWhite	23.60
Iowa	2018	Total	23.60
North Dakota	2009	Hispanic	23.60
South Carolina	2007	NHAsPI	23.60
Oklahoma	2018	Total	23.50
Rhode Island	2013	Total	23.50
Rhode Island	2014	Hispanic	23.50
Nebraska	2010	NHWhite	23.40
California	2018	Total	23.30
Indiana	2013	Hispanic	23.30
Iowa	2007	Hispanic	23.30
Kansas	2013	NHBlack	23.30
Massachusetts	2010	Hispanic	23.30
Mississippi	2007	Hispanic	23.30
Rhode Island	2013	NHWhite	23.20
Indiana	2006	Hispanic	23.10
California	2018	NHAsPI	22.90
Kansas	2011	Hispanic	22.90
Massachusetts	2014	NHAsPI	22.90
Georgia	2006	NHAsPI	22.80
Massachusetts	2012	NHWhite	22.80
Massachusetts	2012	Total	22.80
Minnesota	2011	NHBlack	22.70
Nebraska	2010	Total	22.70
Illinois	2009	Hispanic	22.60
Maine	2012	NHBlack	22.60
Massachusetts	2011	Total	22.60
California	2018	Hispanic	22.50
Minnesota	2012	NHBlack	22.50
Minnesota	2013	Total	22.50
Kansas	2009	Total	22.40
Massachusetts	2010	Total	22.40
Massachusetts	2011	NHWhite	22.40
New Jersey	2012	NHWhite	22.40
Delaware	2007	Total	22.30
Illinois	2007	Hispanic	22.30
Minnesota	2013	Hispanic	22.30
Minnesota	2018	NHAsPI	22.30
Rhode Island	2018	NHWhite	22.30
Georgia	2013	NHBlack	22.20
Iowa	2018	Hispanic	22.20
Kansas	2012	NHBlack	22.20
Massachusetts	2012	Hispanic	22.20
Nebraska	2007	Total	22.20
New Jersey	2009	NHWhite	22.20

New Jersey	2011	NHAsPI	22.20
Minnesota	2011	NHAIAN	22.10
Illinois	2018	NHAIAN	22.00
Massachusetts	2018	NHAsPI	22.00
Nebraska	2007	NHWhite	22.00
Rhode Island	2014	Total	22.00
South Carolina	2007	NHWhite	22.00
Minnesota	2014	NHBlack	21.90
Iowa	2018	NHWhite	21.80
Arkansas	2005	NHAsPI	21.80
Nebraska	2006	NHBlack	21.70
New Jersey	2011	NHWhite	21.70
Georgia	2018	NHAsPI	21.60
Massachusetts	2011	Hispanic	21.60
New Jersey	2014	NHAIAN	21.60
Texas	2005	NHAsPI	21.60
Kansas	2009	Hispanic	21.50
Kansas	2014	Total	21.50
Massachusetts	2010	NHWhite	21.50
Minnesota	2010	NHAIAN	21.50
Minnesota	2009	NHBlack	21.40
Illinois	2009	NHAIAN	21.30
New Jersey	2010	NHWhite	21.20
Minnesota	2014	Total	21.10
Massachusetts	2011	NHAsPI	21.00
Tennessee	2011	NHAIAN	21.00
Delaware	2011	NHBlack	20.80
Massachusetts	2013	NHAsPI	20.80
Minnesota	2012	Hispanic	20.80
Rhode Island	2013	Hispanic	20.80
Minnesota	2014	NHAsPI	20.70
Oklahoma	2018	NHAsPI	20.70
Minnesota	2013	NHWhite	20.60
Kansas	2013	Hispanic	20.50
Maine	2012	NHAsPI	20.50
Massachusetts	2009	Total	20.50
Massachusetts	2012	NHAsPI	20.30
Minnesota	2007	NHAsPI	20.30
Rhode Island	2018	NHBlack	20.30
Delaware	2007	NHBlack	20.20
Indiana	2012	NHAsPI	20.20
Massachusetts	2010	NHAsPI	20.20
Massachusetts	2009	Hispanic	20.10
Minnesota	2010	NHBlack	20.10
Massachusetts	2009	NHAsPI	20.00
Minnesota	2014	NHWhite	20.00

Massachusetts	2009	NHWhite	19.90
Minnesota	2012	Total	19.90
Rhode Island	2011	Hispanic	19.90
Indiana	2013	NHAsPI	19.80
Nebraska	2011	NHAsPI	19.80
Delaware	2009	NHBlack	19.70
Nebraska	2010	Hispanic	19.70
Rhode Island	2014	NHWhite	19.70
Oklahoma	2018	NHAIAN	19.60
West Virginia	2009	NHAsPI	19.60
New Hampshire	2006	NHBlack	19.60
Maine	2018	Total	19.50
Minnesota	2012	NHAsPI	19.50
Kansas	2014	NHWhite	19.40
New Jersey	2010	NHAsPI	19.40
Nebraska	2007	NHAsPI	19.30
Rhode Island	2012	Hispanic	19.30
Nebraska	2007	Hispanic	19.20
Rhode Island	2006	NHAsPI	19.20
California	2007	NHAIAN	19.10
Iowa	2007	NHAsPI	19.00
Massachusetts	2007	NHWhite	19.00
Massachusetts	2007	Total	19.00
California	2009	NHBlack	18.90
Hawaii	2018	Hispanic	18.90
Vermont	2009	NHAsPI	18.80
Georgia	2014	Total	18.70
Minnesota	2012	NHWhite	18.70
Delaware	2010	NHBlack	18.60
South Carolina	2014	NHAsPI	18.60
Hawaii	2011	Total	18.50
Hawaii	2012	Total	18.50
Iowa	2011	NHAsPI	18.50
Rhode Island	2007	NHAsPI	18.50
Rhode Island	2009	NHAsPI	18.50
Hawaii	2013	Total	18.40
Nebraska	2014	NHAsPI	18.40
Rhode Island	2010	NHAsPI	18.40
Iowa	2012	NHAsPI	18.30
Nebraska	2006	NHWhite	18.30
Massachusetts	2006	Total	18.00
Massachusetts	2005	NHWhite	17.90
California	2007	NHBlack	17.90
Georgia	2012	NHBlack	17.90
Iowa	2009	NHAsPI	17.90
Massachusetts	2006	NHWhite	17.90

Massachusetts	2005	Total	17.90
Georgia	2013	Total	17.80
Minnesota	2007	Total	17.70
Kansas	2010	Total	17.60
Nebraska	2005	NHBlack	17.50
California	2007	NHAsPI	17.50
Illinois	2010	NHAsPI	17.50
Arkansas	2005	NHAIAN	17.50
Massachusetts	2007	Hispanic	17.40
Illinois	2009	NHAsPI	17.30
Minnesota	2007	Hispanic	17.30
Nebraska	2010	NHBlack	17.30
Nebraska	2011	NHWhite	17.30
Illinois	2006	NHAsPI	17.20
Minnesota	2011	Total	17.20
Nebraska	2006	Total	17.20
West Virginia	2007	Hispanic	17.10
Iowa	2006	NHAsPI	17.00
Kansas	2014	NHAsPI	17.00
Minnesota	2011	Hispanic	17.00
Kansas	2012	Hispanic	16.90
Mississippi	2005	NHWhite	16.90
Mississippi	2005	NHAsPI	16.80
Minnesota	2009	Total	16.80
South Carolina	2010	NHWhite	16.80
California	2007	Hispanic	16.70
Nebraska	2011	Total	16.70
Nebraska	2005	NHWhite	16.60
Minnesota	2009	NHAsPI	16.50
New Hampshire	2009	NHAsPI	16.40
New Hampshire	2010	NHBlack	16.40
Georgia	2007	NHAsPI	16.30
Hawaii	2011	NHWhite	16.30
Hawaii	2012	NHWhite	16.30
Hawaii	2013	NHWhite	16.30
New Hampshire	2007	NHAsPI	16.20
Tennessee	2009	NHAIAN	16.20
Georgia	2012	Total	16.10
Minnesota	2011	NHAsPI	16.10
Massachusetts	2006	Hispanic	16.10
Kansas	2013	Total	16.00
Minnesota	2010	NHAsPI	16.00
Minnesota	2010	Total	16.00
Tennessee	2007	NHAIAN	16.00
California	2007	Total	15.90
Georgia	2009	NHWhite	15.90

Iowa	2013	NHAsPI	15.90
Kansas	2009	NHBlack	15.90
Mississippi	2006	Hispanic	15.90
Rhode Island	2018	NHAsPI	15.70
Illinois	2005	NHAsPI	15.60
South Carolina	2010	Total	15.60
Georgia	2011	NHBlack	15.50
Massachusetts	2007	NHAsPI	15.50
Minnesota	2011	NHWhite	15.50
Nebraska	2005	Total	15.40
Massachusetts	2006	NHAsPI	15.40
Nebraska	2011	NHAIAN	15.40
Nebraska	2010	NHAIAN	15.30
Kansas	2011	Total	15.20
Minnesota	2009	Hispanic	15.20
Maine	2018	NHWhite	15.10
Iowa	2005	NHAsPI	14.90
Maine	2013	NHAsPI	14.90
Massachusetts	2005	Hispanic	14.80
Nebraska	2009	NHAIAN	14.80
Arkansas	2018	NHAIAN	14.70
Georgia	2009	NHBlack	14.70
Georgia	2011	Total	14.70
New York	2012	NHAIAN	14.60
South Carolina	2013	NHAsPI	14.60
Georgia	2009	Total	14.50
Minnesota	2010	Hispanic	14.40
Nebraska	2011	Hispanic	14.40
California	2012	NHAsPI	14.30
Georgia	2010	NHWhite	14.30
Minnesota	2010	NHWhite	14.30
South Carolina	2009	Hispanic	14.30
Minnesota	2007	NHWhite	14.20
Delaware	2011	NHAsPI	14.00
Georgia	2013	NHWhite	14.00
Massachusetts	2005	NHAsPI	14.00
Georgia	2010	Total	13.90
California	2007	NHWhite	13.70
California	2009	Total	13.70
Georgia	2014	NHWhite	13.60
Nebraska	2005	NHAsPI	13.50
California	2012	NHBlack	13.50
California	2012	Hispanic	13.50
Georgia	2010	NHBlack	13.50
Minnesota	2009	NHWhite	13.50
Mississippi	2006	NHAsPI	13.50

New Hampshire	2006	Total	13.50
Hawaii	2011	NHAsPI	13.30
Hawaii	2012	NHAsPI	13.30
Hawaii	2013	NHAsPI	13.30
Illinois	2007	NHAsPI	13.30
Arkansas	2018	Hispanic	13.20
Georgia	2012	NHWhite	13.20
Kansas	2013	NHWhite	13.20
California	2014	Hispanic	13.10
Nebraska	2012	NHWhite	13.10
California	2009	NHWhite	13.00
California	2014	NHAsPI	13.00
Nebraska	2014	Total	13.00
New Hampshire	2007	Total	13.00
California	2012	Total	12.90
Nebraska	2007	NHAIAN	12.90
Nebraska	2014	NHWhite	12.80
Nebraska	2006	Hispanic	12.70
Arkansas	2011	NHAIAN	12.60
California	2014	Total	12.60
New Hampshire	2010	NHAsPI	12.60
South Carolina	2014	Hispanic	12.40
Georgia	2011	NHWhite	12.30
Georgia	2012	NHAsPI	12.30
New Hampshire	2006	NHAsPI	12.20
Georgia	2013	Hispanic	12.20
Maryland	2018	NHBlack	12.10
California	2009	Hispanic	12.10
Maryland	2018	Total	12.10
California	2009	NHAsPI	12.00
California	2014	NHWhite	12.00
Georgia	2012	Hispanic	12.00
Arkansas	2012	NHAIAN	11.90
New Hampshire	2009	Total	11.90
Ohio	2013	NHAsPI	11.90
Mississippi	2005	Hispanic	11.90
Ohio	2013	NHWhite	11.80
Ohio	2013	NHBlack	11.80
Rhode Island	2011	NHAsPI	11.80
Georgia	2009	Hispanic	11.70
Georgia	2014	Hispanic	11.70
Iowa	2014	NHAIAN	11.70
Kansas	2012	Total	11.70
Nebraska	2012	Total	11.70
Ohio	2013	Total	11.70
California	2010	Hispanic	11.50

California	2011	Hispanic	11.50
California	2012	NHWhite	11.50
Georgia	2014	NHAsPI	11.50
South Carolina	2012	NHAsPI	11.50
Maine	2014	NHAsPI	11.40
Massachusetts	2009	NHAIAN	11.20
Indiana	2006	NHAIAN	11.20
California	2010	Total	11.10
California	2011	Total	11.10
Georgia	2011	Hispanic	11.10
South Carolina	2010	NHBlack	11.00
New Hampshire	2009	NHWhite	10.90
California	2010	NHBlack	10.80
Georgia	2013	NHAsPI	10.80
New Hampshire	2010	Total	10.80
South Carolina	2013	Hispanic	10.80
Nebraska	2005	Hispanic	10.60
California	2010	NHWhite	10.50
Indiana	2005	NHAIAN	10.30
Kansas	2013	NHAsPI	10.30
Nebraska	2011	NHBlack	10.30
California	2011	NHWhite	10.20
Kansas	2009	NHWhite	10.20
Nebraska	2014	NHAIAN	10.20
New Hampshire	2010	NHWhite	10.20
Massachusetts	2010	NHAIAN	10.10
Georgia	2010	Hispanic	10.00
New Hampshire	2005	Total	9.80
Kansas	2010	NHWhite	9.70
California	2011	NHBlack	9.60
Massachusetts	2011	NHAIAN	9.60
South Carolina	2010	NHAsPI	9.60
South Carolina	2014	NHBlack	9.60
Nebraska	2006	NHAIAN	9.40
Rhode Island	2005	NHAsPI	9.40
California	2014	NHBlack	9.30
Kansas	2011	NHWhite	9.30
Maine	2018	NHAsPI	9.30
New Hampshire	2007	NHWhite	9.30
New Hampshire	2007	NHBlack	9.30
Nebraska	2005	NHAIAN	9.30
South Carolina	2014	Total	9.20
Hawaii	2018	NHWhite	9.10
Louisiana	2009	NHAsPI	9.00
South Carolina	2009	Total	9.00
Massachusetts	2012	NHAIAN	8.90

Georgia	2011	NHAsPI	8.80
Hawaii	2018	NHAsPI	8.80
Nebraska	2014	NHBlack	8.80
Hawaii	2018	Total	8.70
Kansas	2010	NHAsPI	8.70
Minnesota	2007	NHAIAN	8.70
Ohio	2013	Hispanic	8.70
South Carolina	2013	NHBlack	8.70
California	2010	NHAsPI	8.50
California	2011	NHAsPI	8.50
South Carolina	2009	NHWhite	8.50
South Carolina	2012	Hispanic	8.50
South Carolina	2013	Total	8.40
New Hampshire	2006	NHWhite	8.40
Massachusetts	2013	NHAIAN	8.30
Massachusetts	2014	NHAIAN	8.10
South Carolina	2009	NHBlack	8.10
Nebraska	2012	NHAsPI	8.00
South Carolina	2014	NHWhite	8.00
South Carolina	2009	NHAsPI	7.80
Kansas	2012	NHWhite	7.70
South Carolina	2013	NHWhite	7.30
Georgia	2010	NHAsPI	7.10
Maryland	2018	NHWhite	7.00
South Carolina	2012	NHBlack	6.90
South Carolina	2012	Total	6.90
Georgia	2009	NHAsPI	6.80
Minnesota	2009	NHAIAN	6.80
South Carolina	2012	NHWhite	6.20
Nebraska	2014	Hispanic	5.90
New Hampshire	2018	NHWhite	5.60
Nebraska	2012	NHAIAN	5.20
Nebraska	2013	NHAIAN	5.20
New Hampshire	2018	Total	5.10
Nebraska	2013	NHAsPI	5.00
New Hampshire	2005	NHWhite	4.90
Delaware	2009	NHAsPI	4.80
Delaware	2010	NHAsPI	4.70
Arkansas	2012	Total	4.10
Rhode Island	2013	NHAsPI	4.10
Rhode Island	2012	NHAsPI	3.90
Rhode Island	2014	NHAsPI	3.80
Nebraska	2013	NHWhite	3.50
Nebraska	2012	NHBlack	3.40
Nebraska	2006	NHAsPI	3.40
Nebraska	2012	Hispanic	3.10

Nebraska	2013	Total	3.10
Maryland	2014	Total	3.00
Maryland	2006	Hispanic	2.80
Maryland	2014	NHBlack	2.80
Maryland	2007	Hispanic	2.40
Maryland	2007	NHBlack	2.30
Maryland	2006	NHBlack	2.20
Maryland	2006	Total	2.10
Maryland	2005	NHBlack	2.10
Maryland	2005	Hispanic	2.00
Maryland	2006	NHWhite	1.90
Maryland	2007	Total	1.90
Maryland	2005	Total	1.90
Maryland	2013	Total	1.80
Maryland	2014	NHWhite	1.80
Maryland	2005	NHAsPI	1.80
Maryland	2006	NHAsPI	1.70
Maryland	2005	NHWhite	1.70
Maryland	2007	NHWhite	1.60
Maryland	2007	NHAsPI	1.60
Maryland	2009	NHBlack	1.60
Maryland	2013	NHWhite	1.60
Maryland	2013	Hispanic	1.50
Maryland	2014	Hispanic	1.40
Maryland	2009	NHWhite	1.30
Maryland	2009	Total	1.30
Maryland	2013	NHBlack	1.30
Maryland	2009	Hispanic	1.20
Maryland	2018	NHAsPI	1.20
Nebraska	2013	NHBlack	1.10
Maryland	2010	NHBlack	1.00
Maryland	2010	Hispanic	1.00
Maryland	2012	NHWhite	1.00
Maryland	2012	Hispanic	1.00
Maryland	2012	Total	1.00
Maryland	2010	NHWhite	0.90
Maryland	2010	Total	0.90
Maryland	2011	NHBlack	0.70
Maryland	2012	NHBlack	0.60
Maryland	2011	NHWhite	0.50
Maryland	2011	Total	0.50
Maryland	2018	Hispanic	0.50
Nebraska	2013	Hispanic	0.50
Maryland	2011	Hispanic	0.40
Maryland	2013	NHAsPI	0.40
Maryland	2014	NHAsPI	0.40

eTable 3.: Average ASD rates by Ethnicity and year.

Years	Indicative Year	Period	Race	ASD_Rate
2003-2007	2005	3	NHAsPI	36.99
2003-2007	2005	3	Hispanic	51.24
2003-2007	2005	3	NHAIAN	61.91
2003-2007	2005	3	NHBlack	87.81
2003-2007	2005	3	NHWhite	44.11
2003-2007	2005	3	Total	50.00
2004-2008	2006	4	NHAsPI	46.24
2004-2008	2006	4	Hispanic	55.22
2004-2008	2006	4	NHAIAN	73.14
2004-2008	2006	4	NHBlack	85.23
2004-2008	2006	4	NHWhite	49.54
2004-2008	2006	4	Total	54.38
2005-2009	2007	5	NHAsPI	46.90
2005-2009	2007	5	Hispanic	57.60
2005-2009	2007	5	NHAIAN	73.52
2005-2009	2007	5	NHBlack	81.46
2005-2009	2007	5	NHWhite	50.55
2005-2009	2007	5	Total	56.35
2007-2011	2009	6	NHAsPI	46.71
2007-2011	2009	6	Hispanic	50.02
2007-2011	2009	6	NHAIAN	86.53
2007-2011	2009	6	NHBlack	70.85
2007-2011	2009	6	NHWhite	51.27
2007-2011	2009	6	Total	56.80
2008-2012	2010	7	NHAsPI	56.05
2008-2012	2010	7	Hispanic	59.09
2008-2012	2010	7	NHAIAN	96.65
2008-2012	2010	7	NHBlack	89.82
2008-2012	2010	7	NHWhite	59.21
2008-2012	2010	7	Total	65.03
2009-2012	2011	8	NHAsPI	56.44
2009-2012	2011	8	Hispanic	67.23
2009-2012	2011	8	NHAIAN	104.73
2009-2012	2011	8	NHBlack	96.26
2009-2012	2011	8	NHWhite	63.31
2009-2012	2011	8	Total	68.75
2010-2014	2012	9	NHAsPI	67.14
2010-2014	2012	9	Hispanic	69.56
2010-2014	2012	9	NHAIAN	127.33
2010-2014	2012	9	NHBlack	95.03
2010-2014	2012	9	NHWhite	65.67
2010-2014	2012	9	Total	70.89
2011-2015	2013	10	NHAsPI	75.05

2011-2015	2013	10	Hispanic	82.86
2011-2015	2013	10	NHAIAN	130.85
2011-2015	2013	10	NHBlack	105.01
2011-2015	2013	10	NHWhite	74.09
2011-2015	2013	10	Total	81.86
2012-2016	2014	11	NHAsPI	77.31
2012-2016	2014	11	Hispanic	84.85
2012-2016	2014	11	NHAIAN	130.97
2012-2016	2014	11	NHBlack	111.47
2012-2016	2014	11	NHWhite	80.33
2012-2016	2014	11	Total	88.62
2016-2020	2018	12	NHAsPI	109.95
2016-2020	2018	12	Hispanic	116.18
2016-2020	2018	12	NHAIAN	149.37
2016-2020	2018	12	NHBlack	150.01
2016-2020	2018	12	NHWhite	106.38
2016-2020	2018	12	Total	113.13

eTable 4.: Mixed Effects regression summary of ASD Rate against ethnicity.
Comparator group is NHAsPI.

Parameter	estimate	Std. Error	t-statistic	P-Value	E-Values	R.R.	Cohen's D
Mixed Effects Regression							
NHWhite	0.738	0.027	27.791	3.62E-142	(3.60, 3.38)	2.09 (1.99, 2.20)	1.30 (1.20, 1.40)
Overall	0.662	0.027	24.939	1.85E-118	(3.29, 3.08)	1.94 (1.84, 2.04)	1.16 (1.07, 1.26)
Hispanic	0.472	0.027	17.654	1.52E-64	(2.59, 2.41)	1.60 (1.52, 1.69)	0.82 (0.73, 0.92)
NHAIAN	0.876	0.028	30.799	2.94E-168	(4.24, 3.97)	2.40 (2.27, 2.54)	1.44 (1.34, 1.54)
NHBlack	0.835	0.027	31.250	3.01E-172	(4.04, 3.80)	2.31 (2.19, 2.43)	1.46 (1.36, 1.56)

eTable 5.: Mixed effects Additive and Interactive models for cannabis exposure against both ethnicity and dichotomized ethnicity.

Parameter				Model	
Parameter	Estimate (C.I.)	P-Value	Cohen's D	Parameter	Value
Bivariate in Dichotomized Race				AIC	1923
ASD ~ fRace				BIC	1945
fRaceNHAA_AIAN	0.22 (0.19, 0.26)	1.23E-30	0.59 (0.49, 0.69)	LogLik	-958
				S.D.	0.382
Bivariate in Cannabis				AIC	1923
ASD ~ Ethnic.Cannabis				BIC	1945
Ethnic.Cannabis	0.18 (0.15, 0.20)	1.38E-35	0.59 (0.50, 0.68)	LogLik	-958
				S.D.	0.382
Additive				AIC	1872
ASD ~ Ethnic.Cannabis + fRace				BIC	1900
fRaceNHAA_AIAN	0.13 (0.09, 0.18)	1.47E-09	0.36 (0.24, 0.48)	LogLik	-931
Ethnic.Cannabis	0.13 (0.09, 0.16)	1.4E-14		S.D.	0.376
Interactive					
ASD ~ Ethnic.Cannabis * fRace				AIC	1875
fRaceNHAA_AIAN	0.11 (0.06, 0.16)	7.25E-06	0.29 (0.16, 0.43)	BIC	1908
Ethnic.Cannabis	0.12 (0.08, 0.15)	5.83E-12		LogLik	-932
fRaceNHAA_AIAN:Ethnic.Cannabis	0.06 (0, 0.12)	0.0390		S.D.	0.375
Interaction Only				AIC	1872
ASD ~ Ethnic.Cannabis : fRace				BIC	1900
fRaceNHAA_AIAN : Ethnic.Cannabis	0.26 (0.22, 0.31)	7.25E-06		LogLik	-931
fRaceOther : Ethnic.Cannabis	0.14 (0.11, 0.17)	5.83E-12	0.0096 (0.0053, 0.0139)	S.D.	0.376
Bivariate in Race					
ASD ~ Race					
Hispanic	0.1 (0.04, 0.15)	0.0016	0.25 (0.09, 0.41)	AIC	1936
NHAIAN	0.28 (0.21, 0.34)	1.33E-17	0.72 (0.56, 0.90)	BIC	1980
NHBlack	0.3 (0.24, 0.36)	1.56E-22	0.78 (0.62, 0.94)	LogLik	-960
NHWhite	0.05 (-0.01, 0.11)	0.0859	0.13 (0.02, 0.29)	S.D.	0.38
Overall	0.11 (0.05, 0.17)	2.70E-04	0.28 (0.13, 0.44)		
Additive					
ASD ~ Ethnic.Cannabis + Race					
Hispanic	-0.11 (-0.18, -0.04)	0.0025	0.29 (0.10, 0.50)	AIC	1857
NHAIAN	-0.14 (-0.24, -0.03)	0.0114	0.37 (0.07, 0.66)	BIC	1907
NHBlack	-0.08 (-0.18, 0.01)	0.0955	0.22 (0.05, 0.50)	LogLik	-920

NHWhite	-0.29 (-0.38, -0.2)	7.66E-10	0.77 (0.52, 1.02)	S.D.	0.372
Overall	-0.2 (-0.28, -0.11)	8.21E-06	0.52 (0.29, 0.76)		
Ethnic.Cannabis	0.25 (0.2, 0.31)	1.79E-20			
Interactive					
ASD ~ Ethnic.Cannabis * Race					
Hispanic	-0.91 (-1.29, -0.54)	2.05E-06	2.42 (1.40, 3.45)	AIC	1847
NHAIAN	-1.05 (-1.45, -0.66)	2.02E-07	2.80 (1.72, 3.88)	BIC	1925
NHBlack	-0.92 (-1.3, -0.53)	4.24E-06	2.41 (1.36, 3.47)	LogLik	-910
NHWhite	-1.12 (-1.5, -0.74)	1.35E-08	2.97 (1.92 4.02)	S.D.	0.369
Overall	-1.04 (-1.42, -0.66)	1.16E-07	2.75 (1.71, 3.79)		
Ethnic.Cannabis	1.02 (0.68, 1.36)	6.18E-09			
Hispanic: Ethnic.Cannabis	-0.62 (-0.96, -0.29)	0.0002			
NHAIAN: Ethnic.Cannabis	-0.63 (-0.96, -0.3)	0.0002			
NHBlack: Ethnic.Cannabis	-0.79 (-1.12, -0.46)	3.26E-06			
NHWhite: Ethnic.Cannabis	-0.8 (-1.13, -0.47)	2.56E-06			
Interaction Only					
ASD ~ Ethnic.Cannabis : Race					
NHAsPI : Ethnic.Cannabis	0.12 (0.08, 0.16)	1.97E-08			
Hispanic : Ethnic.Cannabis	0.27 (0.19, 0.35)	3.55E-12	0.011 (0.005, 0.018)	AIC	1857
NHAIAN : Ethnic.Cannabis	0.30 (0.24, 0.37)	3.56E-19	0.014 (0.007, 0.020)	BIC	19078
NHBlack : Ethnic.Cannabis	0.25 (0.18, 0.31)	5.73E-14	0.010 (0.003, 0.016)	LogLik	-920
NHWhite : Ethnic.Cannabis	0.09 (0.02, 0.17)	0.0097	-0.002 (-0.009, 0.005)	S.D.	0.372
NHOverall : Ethnic.Cannabis	0.18 (0.11, 0.26)	2.74E-06	0.005 (-0.002, 0.012)		

eTable 6.: Mixed Effects Model Including

Case Ascertainment and Legal Status

Multiple Regression Summary Table

**Parameters**			**Model**	
**Parameter**	**Estimate (C.I.)**	**P-Value**	**Parameter**	**Value**
Ethnic.Cannabis	0.49 (0.27, 0.70)	1.18E-05	AIC	3117
Race.NHAIAN	-0.08 (-0.24, 0.07)	0.2978	BIC	3227
Race.NHAsPI	-0.02 (-0.29, 0.26)	0.9101	LogLik	-1538
Race.NHBlack	0.11 (-0.02, 0.24)	0.0839	S.D.	0.512
Race.NHWhite	-0.14 (-0.25, -0.03)	0.0118		
Race.Total	-0.06 (-0.15, 0.04)	0.2609		
Median Income	0.59 (0.20, 0.98)	0.0031		
Status.Illegal	0.06 (-0.20, 0.33)	0.6390		
Status.Legal	0.26 (-0.04, 0.55)	0.0949		
Status.Medical	0.16 (-0.11, 0.44)	0.2374		
Ascertainment.Active	-0.03 (-0.17, 0.11)	0.6512		
Ascertainment.Mixed	-0.03 (-0.14, 0.07)	0.5271		
Ethn.Cannabis: Race.NHAIAN	0.07 (-0.18, 0.32)	0.5919		
Ethn.Cannabis: Race.NHAsPI	-0.33 (-0.54, -0.11)	0.0026		
Ethn.Cannabis: Race.NHBlack	-0.21 (-0.44, 0.02)	0.0767		
Ethn.Cannabis: Race.NHWhite	-0.28 (-0.51, -0.05)	0.0171		
Ethn.Cannabis: Race.Total	-0.20 (-0.43, 0.03)	0.0819		

Anova Table:

**Parameter**	**Sum of Squares**	**Mean Squares**	**Numerator Deg. Freedom**	**Denominator Deg. Freedom**	**F-Value**	**P-Value**
Ethnic.Cannabis	3.7682	3.7682	1	1862.823	14.3889	1.53E-04
Race	5.4665	1.0933	5	1830.614	4.1748	8.94E-04
Median Income	2.2950	2.2950	1	1849.740	8.7636	0.0031
Legal Status	1.1991	0.3997	3	1835.104	1.5262	0.2058
Case Ascertainment	0.1079	0.0540	2	1858.893	0.2060	0.8138
Ethn.Cannabis: Race	4.3693	0.8739	5	1823.147	3.3369	0.0053

Line Slopes by Ethnicity (from emmeans::emtrends)

Race	Ethnic.Cannabis Trend (Slope)	P-Value
Hispanic	0.49 (0.27, 0.70)	<0.0001
NHAIAN	0.56 (0.29, 0.82)	<0.0001
NHAsPI	0.16 (-0.04, 0.36)	0.1198
NHBlack	0.28 (0.05, 0.51)	0.0174
NHWhite	0.21 (-0.02, 0.44)	0.0774
Overall	0.29 (0.07, 0.51)	0.0112

eTable 7.: Hybrid mixed effects modelling of cannabis exposure as both a within-state and between-state covariate. Bivariate, additive, interactive and Mundlak models.

**Parameter**			**Model**	
**Parameter**	**Estimate (C.I.)**	**P-Value**	**Parameter**	**Value**
Bivariate - Cigarettes			AIC	1956
ASD ~ Within.Cigarettes + Between.Cigarettes			BIC	1984
Within_Cigarette	-0.25 (-0.33, -0.16)	7.62E-09	LogLik	-973
Between_Cigarette	0.15 (0.05, 0.25)	3.37E-03	S.D.	0.327
			Conditional	0.78 (0.74, 0.83)
			Marginal	0.03 (0.00, 0.09)
Bivariate - Cannabis			AIC	1817
ASD ~ Within.Cannabis + Between.Cannabis			BIC	1844
Within_Cannabis	0.35 (0.30, 0.41)	9.08E-37	LogLik	-903
Between_Cannabis	0.25 (0.14, 0.35)	3.31E-06	S.D.	0.314
			Conditional	0.80 (0.77, 0.83)
			Marginal	0.08 (0.04, 0.12)
Additive				
ASD ~ Within_Cannabis + Between_Cannabis + Within_Cigarette + Between_Cigarette + Ethn.Analgesics + Ethn.Bing.Alcohol + Ethn.Cocaine + Med.Income + Race				
Within_Cannabis	0.4 (0.34, 0.46)	1.04E-34	AIC	1754
Between_Cannabis	0.5 (0.35, 0.65)	3.85E-10	BIC	1837
Within_Cigarette	0.21 (0.1, 0.32)	0.0001	LogLik	-862
Between_Cigarette	0.49 (0.26, 0.73)	6.01E-05	S.D.	0.307
Ethn.Analgesics	-0.09 (-0.15, -0.02)	0.0070	Conditional	0.81 (0.79, 0.84)
Ethn.Bing.Alcohol	-0.84 (-1.25, -0.44)	4.69E-05	Marginal	0.16 (0.11, 0.24)
Ethn.Cocaine	-0.12 (-0.15, -0.08)	5.15E-10		
RaceNHAIAN	-0.83 (-1.54, -0.12)	0.0224		
RaceNHBlack	-0.59 (-1.13, -0.06)	0.0307		
RaceNHWhite	-0.68 (-1.27, -0.1)	0.0226		
RaceTotal	-0.53 (-1.05, 0)	0.0493		
Interactive (Mundlak)				
ASD ~ Within_Cannabis * Between_Cannabis * Between_Cigarette + Within_Cigarette + Ethn.Analgesics + Ethn.Cocaine + Med.Income + Race				
Within_Cannabis	0.41 (0.34, 0.48)	1.49E-29	AIC	1750
Between_Cannabis	0.50 (0.34, 0.65)	6.13E-10	BIC	1844
Between_Cigarette	0.49 (0.25, 0.73)	7.97E-05	LogLik	-858
Within_Cigarette	0.18 (0.07, 0.29)	0.0015	S.D.	0.306
Ethn.Analgesics	-0.08 (-0.15, -0.02)	0.0120	Conditional	0.81 (0.79, 0.84)
Ethn.Bing.Alcohol	-0.71 (-1.13, -0.30)	0.0008	Marginal	0.16 (0.12, 0.25)
Ethn.Cocaine	-0.11 (-0.15, -0.08)	5.17E-10		

RaceNHAIAN	-0.88 (-1.60, -0.17)	0.0155		
RaceNHBlack	-0.64 (-1.17, -0.10)	0.0203		
RaceNHWhite	-0.75 (-1.34, -0.16)	0.0128		
RaceTotal	-0.59 (-1.12, -0.06)	0.0293		
Within_Cannabis:Between_Cannabis	-0.17 (-0.26, -0.07)	0.0005		
Within_Cannabis:Between_Cigarette	0.15 (0.05, 0.25)	0.0039		

eTable 8.: Model Comparison table for the Overall Model Parameters of eTable 5.
See also eFigure 5.

Name	Model	AIC	AIC_wt	AICc	AICc_wt	BIC	BIC_wt	R2_conditional	R2_marginal	Sigma
Cigarette_Hybrid	lmerModLmerTest	1942.791	1.75E-54	1942.823	2.04E-54	1970.491	2.60E-46	0.7850	0.0316	0.3270
Cannabis_Hybrid	lmerModLmerTest	1802.435	5.27E-24	1802.467	6.12E-24	1830.136	7.82E-16	0.8017	0.0800	0.3143
Comprehensive_Additive_Hybrid	lmerModLmerTest	1710.706	4.37E-04	1710.802	4.91E-04	1760.566	1.0000	0.8070	0.1264	0.3070
Comprehensive_Interactive_Hybrid	lmerModLmerTest	1695.237	0.9996	1695.566	0.9995	1789.419	5.43E-07	0.8098	0.1561	0.3056

eTable 9.: Mixed Effects Analysis of Dichotomized Ethnicity.

Parameter					Model			
**Parameter**	**Value**	**Std.Error**	**t-value**	**P-Value**	**AIC**	**BIC**	**LogLik**	**S.D.**
Factor Race Model								
ASD ~ fRace								
NHAA_AIAN	0.289	0.027	10.862	1.11E-26	3177.37	3199.53	-1584.685	0.531
THC Exposure Model								
ASD ~ Cannabis_Grouping								
Ethnic Cannabis * THC Exposure	0.641	0.033	19.199	5.00E-75	3933.70	3955.85	-1962.849	0.667
Additive Model								
ASD ~ Ethnic Cannabis * THC Exposure + Race_Group								
Ethnic Cannabis * THC Exposure	0.173	0.018	9.547	4.10E-21	3096.517	3124.209	-1543.258	0.518
NHAA_AIAN	0.178	0.028	6.259	4.28E-10				
Interactive Model								
ASD ~ Ethnic Cannabis * THC Exposure * Race_Group								
Ethnic Cannabis * THC Exposure	0.163	0.018	8.831	2.36E-18	3096.406	3129.633	-1542.203	0.517
NHAA_AIAN	0.193	0.029	6.652	3.80E-11				
Race_Group: Ethnic THC	0.129	0.052	2.489	0.0129				
Interactive Only Model								
ASD ~ Ethnic Cannabis * THC Exposure : Race_Group								
Race_Group: Ethnic THC_NHAA_AIAN	0.273	0.0519	5.26	1.57E-07	3132.918	3160.61	-1561.459	0.523
Race_Group: Ethnic THC_Other	0.217	0.0169	12.9	2.71E-36				

eTable 10.: Mixed Effects Analysis of Ethnic Δ9THC Exposure.

Parameters			Model	
**Parameter**	**Estimate (C.I.)**	**P-Value**	**Metric**	**Value**
Additive Model				
ASD ~ eCigarettes + eTHC + eAUD + eAnalgesics + eCocaine + Income + Race				
Ethn.Cigarettes	2.48 (1.36, 3.6)	1.54E-05	AIC	3071.487
Ethn.THC	0.22 (0.13, 0.31)	3.17E-06	BIC	3137.904
Ethn.Analgesics	-7.05 (-11.6, -2.5)	0.003	logLik	-1523.744
Median.Income	0.61 (0.16, 1.06)	0.008	S.D.	0.5114
RaceNHAsPI	0.47 (0.19, 0.75)	0.001	Marginal*	1.2461
RaceTotal	0.14 (0.06, 0.22)	0.002	Marginal P	0.0330
RaceHispanic	0.28 (0.16, 0.41)	6.53E-06		
RaceNHAIAN	0.13 (0.01, 0.25)	0.027		
RaceNHBlack	0.27 (0.17, 0.36)	4.34E-08		
*Interactive eTHC * eCigarettes*				
ASD ~ eCigarettes * eTHC + eAUD + eAnalgesics + eCocaine + Income + Race				
Ethn.Cigarettes	2.71 (1.57, 3.85)	0.000	AIC	3082.071
Ethn.THC	0.16 (0.02, 0.3)	0.033	BIC	3176.116
RaceNHAsPI	0.34 (0.03, 0.65)	0.033	logLik	-1524.035
RaceTotal	0.14 (0.05, 0.23)	0.003	S.D.	0.5098
RaceHispanic	0.39 (0.25, 0.54)	7.49E-08	Marginal*	1.3512
RaceNHAIAN	0.14 (0.02, 0.26)	0.023	Marginal P	7.28E-07
RaceNHBlack	0.27 (0.17, 0.37)	3.74E-08		
Ethn.Analgesics	-7.88 (-12.54, -3.22)	0.001		
Median.Income	0.59 (0.14, 1.03)	0.010		
Ethn.THC:RaceHispanic	0.21 (0.05, 0.37)	0.011		
Ethn.THC:RaceNHAIAN	0.23 (0.04, 0.42)	0.015		
*Interactive eTHC * Race*			AIC	3062.281
ASD ~ eCigarettes + eTHC * Race + eAUD + eAnalgesics + eCocaine + Income			BIC	3128.698
Ethn.Cigarettes	2.53 (1.41, 3.65)	9.49E-06	logLik	-1519.141
Ethn.Analgesics	-6.41 (-10.96, -1.86)	0.006	S.D.	0.5104
Median.Income	0.68 (0.27, 1.09)	0.001	Marginal*	1.3191
Ethn.Cigarettes: Ethn.THC	0.96 (0.61, 1.31)	1.15E-07	Marginal P	1.11E-07
*Interactive eTHC * cCigarettes * Race*				
ASD ~ eCigarettes * eTHC * Race + eAUD + eAnalgesics + eCocaine + Income				
Ethn.Cigarettes	16.03 (7.05, 25.01)	0.001	AIC	3034.568
Ethn.Cigarettes: Ethn.THC	3.96 (1.73, 6.19)	0.001	BIC	3156.233
RaceHispanic	0.66 (0.17, 1.15)	0.008	logLik	-1495.293
RaceNHAIAN	0.66 (0.14, 1.18)	0.014	S.D.	0.5067
Median.Income	0.57 (0.14, 1)	0.009	Marginal*	1.6161
Ethn.Cigarettes: Ethn.THC: RaceNHAIAN	-2.75 (-4.91, -0.59)	0.013	Marginal P	2.42E-08
Ethn.Cigarettes: Ethn.THC: RaceTotal	-3.02 (-5.17, -0.87)	0.006		
Ethn.Cigarettes: Ethn.THC: RaceNHWhite	-3.05 (-5.2, -0.9)	0.006		
Ethn.Cigarettes: Ethn.THC: RaceNHBlack	-3.1 (-5.26, -0.93)	0.005		
Ethn.Analgesics	-7.51 (-12.17, -2.85)	0.002		
Ethn.Cigarettes: RaceNHWhite	-11.26 (-19.87, -2.65)	0.010		

Ethn.Cigarettes: RaceTotal	-12.29 (-20.95, -3.64)	0.005		
Ethn.Cigarettes: RaceNHBlack	-12.76 (-21.38, -4.14)	0.004		
Ethn.Cigarettes: RaceHispanic	-13.15 (-21.71, -4.6)	0.003		
Ethn.Cigarettes: RaceNHAIAN	-14.02 (-22.76, -5.28)	0.002		
*Interactive eCBD * cCigarettes * Race*				
ASD ~ eCigarettes * eCBD * Race + eAUD + eAnalgesics + eCocaine + Income				
Ethn.Cigarettes	-23.2 (-39.6, -6.78)	0.006	AIC	3084
Ethn.CBD	0.273 (0.105, 0.44)	0.002	BIC	3211
Ethn.Analgesics	-6.88 (-11.6, -2.19)	0.004	S.D.	0.51
Median.Income	1.12 (0.742, 1.49)	6.73E-09	LogLik	-1519
Ethn.Cigarettes: Ethn.CBD	-5.17 (-8.25, -2.08)	0.001	Marginal*	
Ethn.Cigarettes: RaceHispanic	19.5 (7.1, 31.9)	0.002	Marginal P	
Ethn.Cigarettes: RaceNHAIAN	22.9 (8.61, 37.1)	0.002		
Ethn.Cigarettes: RaceNHBlack	22.1 (8.72, 35.5)	0.001		
Ethn.Cigarettes: RaceNHWhite	20.8 (7.17, 34.4)	0.003		
Ethn.Cigarettes: RaceTotal	20.7 (7.33, 34.1)	0.002		
Ethn.CBD: RaceNHWhite	0.22 (0.09, 0.35)	7.03E-04		
Ethn.CBD: RaceTotal	0.11 (0, 0.21)	0.043		
Ethn.Cigarettes: Ethn.CBD: RaceHispanic	4.38 (2.12, 6.63)	1.48E-04		
Ethn.Cigarettes: Ethn.CBD: RaceNHAIAN	4.53 (1.95, 7.11)	5.97E-04		
Ethn.Cigarettes: Ethn.CBD: RaceNHBlack	4.01 (1.61, 6.41)	0.001		
Ethn.Cigarettes: Ethn.CBD: RaceNHWhite	3.47 (1.02, 5.92)	0.006		
Ethn.Cigarettes: Ethn.CBD: RaceTotal	3.73 (1.3, 6.17)	0.003		

eTable 11.: Effects sizes for Model 4 from eTable 8 for Ethnicity as a Function of both Δ9THC and Cannabidiol.

Race	emmean C.I.)	P-Value	emtrends (C.I.)	P-Value	CohensD
**Δ9THC**					
NHAsPI	6.28 (4.69, 7.86)	1.49E-14	0.90 (0.39, 1.40)	0.0005	0.36 (0.27, 0.46)
Hispanic	4.08 (3.74, 4.42)	1.89E-33	0.46 (0.27, 0.65)	1.37E-06	6.02 (4.86, 7.17)
NHAIAN	3.92 (3.57, 4.28)	1.59E-34	0.27 (0.16, 0.39)	2.22E-06	5.11 (4.17, 6.04)
NHBlack	3.98 (3.67, 4.30)	1.30E-28	0.20 (0.06, 0.33)	0.0038	7.52 (5.87, 9.15)
NHWhite	3.63 (3.31, 3.96)	6.39E-28	0.21 (0.08, 0.33)	0.0014	6.43 (5.05, 7.8)
Total	3.84 (3.53, 4.16)	1.87E-27	0.21 (0.08, 0.35)	0.0018	7.47 (5.8, 9.13)
**Cannabidiol**					
NHAsPI	2.77 (1.60, 3.93)	3.77e- 6	-0.89 (-1.45, -0.34)	0.002	0.23 (0.13, 0.33)
Hispanic	3.84 (3.49, 4.18)	5.78E-29	-0.01 (-0.16, 0.14)	0.862	6.09 (4.82, 7.35)
NHAIAN	3.94 (3.57, 4.32)	2.55E-33	0.16 (0.04, 0.29)	0.012	4.90 (3.99, 5.80)
NHBlack	4.01 (3.68, 4.34)	1.27E-27	0.10 (0.00, 0.20)	0.053	7.31 (5.69, 8.92)
NHWhite	3.66 (3.33, 4.00)	8.30E-27	0.11 (0.02, 0.20)	0.023	6.31 (4.93, 7.68)
Total	3.85 (3.53, 4.18)	1.38E-26	0.05 (-0.04, 0.15)	0.254	7.16 (5.54, 8.76)

eTable 12.: Effects Sizes for Model 4 from eTable 8 for Contrasts between the Different Levels of Ethnicity as a Function of both Δ9THC and Cannabidiol.

Contrast	effectsize (C.I.)	P-Value	emmeans (C.I.)	P-Value	CohensD	emtrends (C.I.)	P-Value
**Δ9THC**							
NHAsPI - Hispanic	4.34 (1.26, 7.42)	0.006	2.2 (-0.02, 4.42)	0.055	-2.12	0.43 (-0.28, 1.15)	0.5152
NHAsPI - NHAIAN	4.64 (1.41, 7.88)	0.005	2.35 (0.01, 4.70)	0.048	-2.33	0.62 (-0.09, 1.33)	0.1276
NHAsPI - NHBlack	4.52 (1.33, 7.72)	0.007	2.29 (0.00, 4.58)	0.049	-2.46	0.70 (-0.01, 1.41)	0.0568
NHAsPI - NHWhite	5.22 (2.00, 8.43)	0.002	2.64 (0.33, 4.95)	0.014	-1.77	0.69 (-0.02, 1.40)	0.0611
NHAsPI - Total	4.80 (1.61, 7.99)	0.004	2.43 (0.15, 4.72)	0.029	-2.15	0.68 (-0.03, 1.39)	0.0668
Hispanic - NHAIAN	0.30 (-0.21, 0.82)	0.242	0.15 (-0.22, 0.52)	0.846	-0.21	0.19 (-0.10, 0.47)	0.4297
Hispanic - NHBlack	0.18 (-0.19, 0.56)	0.327	0.09 (-0.17, 0.36)	0.921	-0.35	0.27 (-0.03, 0.56)	0.1067
Hispanic - NHWhite	0.88 (0.47, 1.28)	0.000	0.44 (0.16, 0.73)	0.000	0.34	0.26 (-0.04, 0.55)	0.1247
Hispanic - Total	0.46 (0.12, 0.81)	0.010	0.23 (-0.01, 0.48)	0.077	-0.03	0.25 (-0.05, 0.54)	0.1603
NHAIAN - NHBlack	-0.12 (-0.51, 0.27)	0.536	-0.06 (-0.34, 0.22)	0.989	-0.14	0.08 (-0.14, 0.30)	0.9103
NHAIAN - NHWhite	0.57 (0.2, 0.94)	0.003	0.29 (0.03, 0.55)	0.023	0.55	0.07 (-0.14, 0.28)	0.9424
NHAIAN - Total	0.16 (-0.21, 0.53)	0.395	0.08 (-0.19, 0.35)	0.956	0.18	0.06 (-0.16, 0.28)	0.9690
NHBlack - NHWhite	0.69 (0.45, 0.94)	<.0001	0.35 (0.18, 0.53)	<.0001	0.69	-0.01 (-0.24, 0.22)	1.0000
NHBlack - Total	0.28 (0.08, 0.47)	0.006	0.14 (0.00, 0.28)	0.047	0.31	-0.02 (-0.25, 0.22)	0.9999
NHWhite - Total	-0.41 (-0.64, -0.19)	0.001	-0.21 (-0.37, -0.05)	0.002	-0.38	-0.01 (-0.24, 0.22)	1.0000
**Cannabidiol**							
NHAsPI - Hispanic	-2.1 (-4.29, 0.09)	0.059	-1.07 (-2.66, 0.52)	0.386	-2.10	-0.88 (-1.64, -0.12)	0.012
NHAsPI - NHAIAN	-2.31 (-4.64, 0.02)	0.052	-1.18 (-2.88, 0.52)	0.355	-2.31	-1.05 (-1.97, -0.14)	0.013
NHAsPI - NHBlack	-2.45 (-4.73, -0.16)	0.036	-1.25 (-2.90, 0.40)	0.258	-2.45	-0.99 (-1.83, -0.16)	0.009
NHAsPI - NHWhite	-1.76 (-4.06, 0.54)	0.131	-0.9 (-2.56, 0.77)	0.640	-1.76	-1.00 (-1.86, -0.15)	0.010
NHAsPI - Total	-2.14 (-4.42, 0.15)	0.067	-1.09 (-2.74, 0.56)	0.414	-2.13	-0.95 (-1.78, -0.12)	0.014
Hispanic - NHAIAN	-0.21 (-0.69, 0.27)	0.385	-0.11 (-0.46, 0.24)	0.952	-0.21	-0.17 (-0.49, 0.15)	0.631
Hispanic - NHBlack	-0.34 (-0.65, -0.04)	0.029	-0.18 (-0.40, 0.05)	0.211	-0.34	-0.11 (-0.38, 0.15)	0.842
Hispanic - NHWhite	0.34 (0.00, 0.68)	0.048	0.17 (-0.07, 0.42)	0.328	0.34	-0.12 (-0.39, 0.15)	0.788
Hispanic - Total	-0.03 (-0.32, 0.26)	0.820	-0.02 (-0.23, 0.19)	1.000	-0.03	-0.07 (-0.33, 0.19)	0.976
NHAIAN - NHBlack	-0.14 (-0.53, 0.27)	0.501	-0.07 (-0.36, 0.22)	0.984	-0.13	0.06 (-0.15, 0.28)	0.959

NHAIAN - NHWhite	0.55 (0.17, 0.93)	0.005	0.28 (0.01, 0.56)	0.042	0.55	0.05 (-0.15, 0.25)	0.978
NHAIAN - Total	0.18 (-0.21, 0.56)	0.360	0.09 (-0.19, 0.37)	0.940	0.18	0.11 (-0.11, 0.32)	0.720
NHBlack - NHWhite	0.69 (0.45, 0.92)	<.0001	0.35 (0.18, 0.52)	<.0001	0.68	-0.01 (-0.20, 0.18)	1.000
NHBlack - Total	0.31 (0.12, 0.50)	0.002	0.16 (0.02, 0.30)	0.013	0.31	0.04 (-0.15, 0.23)	0.988
NHWhite - Total	-0.37 (-0.59, -0.16)	0.001	-0.19 (-0.35, -0.04)	0.007	-0.37	0.06 (-0.13, 0.24)	0.956

eTable 13.: ASDR – Cannabis Regression Slopes from Mixed Models by Ethnicity (from emmeans)

Race	Elasticity (β, C.I.)	P-Value
NHAIAN	0.61 (0.33,0.88)	<0.0001
Hispanic	0.53 (0.31,0.76)	<0.0001
NHBlack	0.33 (0.09,0.57)	0.0068
NHWhite	0.26 (0.02,0.49)	0.0370
NHAsPI	0.20 (0.00,0.41)	0.0514

eTable 14.: ASDR – Cannabis Regression Slopes from Mixed Models by Ethnicity

Under Paradigms of 50% and 100% Increased Cannabis Exposure (from emmeans)

**Race**	**Elasticity (C.I.)**	**1.5 x Cannabis Δ% (C.I.)**	**2.0 x Cannabis Δ% (C.I.)**
NHAIAN	0.61 (0.33, 0.88)	27.86 (14.34, 44.66)	52.22 (25.74, 84.27)
Hispanic	0.53 (0.31, 0.76)	24.14 (13.33, 37.36)	44.73 (23.86, 69.12)
NHBlack	0.33 (0.09, 0.57)	14.27 (3.75, 26.81)	25.62 (6.49, 48.18)
NHWhite	0.25 (0.02, 0.49)	10.88 (0.63, 22.98)	19.31 (1.07, 40.83)
NHAsPI	0.20 (0.00, 0.41)	8.59 (-0.05, 18.61)	15.13 (-0.08, 32.66)

eTable 15.: Panel Models of Ethnic Cannabis, Ethnicity and Income

Parameters			Model	
**Parameter**	**Estimate (C.I.)**	**P-Value**	**Metric**	**Value**
Overall Panel Model				
*ASD ~ Ethn.Cannabis * Race + Income, Model = Random*				
Ethnic.Cannabis	0.37 (0.16, 0.59)	6.41E-04	R.Squared	0.1765
Race.Hispanic	-0.26 (-0.73, 0.21)	0.2750	Adj.R.Squared	0.1712
Race.NHAIAN	-0.28 (-0.82, 0.26)	0.3113	F Statistic	101.07
Race.NHBlack	-0.36 (-0.86, 0.14)	0.1555	Model P-Value	3.32E-17
Race.NHWhite	-0.55 (-1.03, -0.06)	0.0282		
Median Income	0.13 (-0.23, 0.5)	0.4680		
Ethn.Cannabis: Race.Hispanic	0.09 (-0.23, 0.4)	0.5885		
Ethn.Cannabis: Race.NHAIAN	0.37 (-0.08, 0.83)	0.1096		
Ethn.Cannabis: Race.NHBlack	0.32 (-0.02, 0.66)	0.0662		
Ethn.Cannabis: Race.NHWhite	0.13 (-0.21, 0.47)	0.4430		
Within States Variation				
*ASD ~ Ethn.Cannabis * Race + Income, Model = Within*				
Ethnic.Cannabis	0.16 (-0.04, 0.37)	0.1230	R.Squared	0.1370
Race.Hispanic	0 (-0.28, 0.27)	0.9730	Adj.R.Squared	0.1080
Race.NHAIAN	-0.09 (-0.46, 0.28)	0.6160	F Statistic	23.800
Race.NHBlack	0.12 (-0.23, 0.46)	0.5040	Model P-Value	4.67E-42
Race.NHWhite	-0.13 (-0.46, 0.19)	0.4210		
Median Income	0.85 (0.4, 1.3)	0.0002		
Ethn.Cannabis: Race.Hispanic	0.33 (0.1, 0.55)	0.0041		
Ethn.Cannabis: Race.NHAIAN	0.4 (0.14, 0.65)	0.0026		
Ethn.Cannabis: Race.NHBlack	0.12 (-0.11, 0.35)	0.3020		
Ethn.Cannabis: Race.NHWhite	0.05 (-0.18, 0.27)	0.6930		
Between States Variation				
*ASD ~ Ethn.Cannabis * Race + Income, Model = Between*				
Ethnic.Cannabis	23.55 (-7.15, 54.25)	0.1429	R.Squared	0.5780
Race.Hispanic	-50.5 (-113.66, 12.66)	0.1273	Adj.R.Squared	0.4410
Race.NHAIAN	-48.43 (-108.67, 11.81)	0.1253	F Statistic	4.2400
Race.NHBlack	27.49 (-56.18, 111.15)	0.5244	Model P-Value	0.0009
Race.NHWhite	-103.96 (-218.39, 10.46)	0.0847		
Median Income	-1.74 (-3.61, 0.13)	0.0775		
Ethn.Cannabis: Race.Hispanic	0.89 (-52.4, 54.18)	0.9741		
Ethn.Cannabis: Race.NHAIAN	-12.07 (-42.56, 18.43)	0.4439		
Ethn.Cannabis: Race.NHBlack	-161.78 (-324.07, 0.52)	0.0598		
Ethn.Cannabis: Race.NHWhite	59.86 (-65.26, 184.98)	0.3557		

Mundlak Model				
*ASD ~ Ethn.Cannabis_Within * Race + Income + Ethn.Cannabis_Between + Income_Between, Model = Random*				
Ethnic.Cannabis.Within	0.23 (0.01, 0.45)	0.0435	R.Squared	0.1930
Race.Hispanic	-1.32 (-2, -0.64)	0.0001	Adj.R.Squared	0.1867
Race.NHAIAN	-1.57 (-2.45, -0.68)	0.0005	F Statistic	149.3884
Race.NHBlack	-1.65 (-2.5, -0.8)	0.0001	Model P-Value	7.54E-26
Race.NHWhite	-1.86 (-2.67, -1.04)	7.00E-06		
Median.Income.Within	0.74 (0.35, 1.13)	0.0002		
Ethnic.Cannabis.Between	1.2 (0.77, 1.63)	5.04E-08		
Median.Income.Between	-2.49 (-3.36, -1.62)	2.10E-08		
Ethn.Cannabis: Race.Hispanic	0.03 (-0.29, 0.34)	0.8561		
Ethn.Cannabis: Race.NHAIAN	0.37 (-0.12, 0.85)	0.1379		
Ethn.Cannabis: Race.NHBlack	0.32 (-0.03, 0.68)	0.0711		
Ethn.Cannabis: Race.NHWhite	0.1 (-0.25, 0.45)	0.5687		

eTable 16.: Difference in Difference Analysis – Estimates and confidence Intervals

Estimates and Confidence Intervals:

Event_time	NHWhite	NHBlack	Hispanic	NHAsPI	NHAIAN
-4	-0.11 (-0.41, 0.19)	0.10 (-0.18, 0.37)	0.07 (-0.27, 0.42)	0.09 (-0.32, 0.51)	-0.00 (-0.52, 0.51)
-3	-0.20 (-0.42, 0.03)	-0.02 (-0.22, 0.18)	0.01 (-0.27, 0.29)	0.12 (-0.15, 0.39)	-0.04 (-0.43, 0.36)
-2	-0.16 (-0.31, -0.01)	0.01 (-0.16, 0.18)	-0.01 (-0.22, 0.20)	0.08 (-0.16, 0.32)	0.04 (-0.27, 0.35)
0	-0.23 (-0.43, -0.04)	0.08 (-0.10, 0.26)	-0.04 (-0.23, 0.15)	-0.00 (-0.24, 0.23)	0.17 (-0.17, 0.52)
1	-0.03 (-0.59, 0.53)	0.40 (-0.15, 0.95)	-0.25 (-0.61, 0.11)	0.09 (-0.14, 0.33)	0.35 (-0.62, 1.33)
2	-0.42 (-0.80, -0.05)	0.10 (-0.25, 0.44)	-0.19 (-0.54, 0.15)	-0.04 (-0.43, 0.34)	-0.22 (-0.68, 0.25)
3	-0.35 (-0.69, -0.01)	0.11 (-0.21, 0.43)	-0.12 (-0.43, 0.19)	0.08 (-0.44, 0.60)	-0.41 (-1.00, 0.18)
4	-0.31 (-0.78, 0.16)	0.24 (0.09, 0.39)	0.08 (-0.32, 0.49)	-0.05 (-0.45, 0.36)	-0.33 (-1.04, 0.38)

Note: “Event_time” Relates to periods from four years prior to- and four years after- the relaxation of cannabis legislation.

P-Values

Event_time	NHWhite	NHBlack	Hispanic	NHAsPI	NHAIAN
-4	0.4597	0.4642	0.6596	0.6401	0.9912
-3	0.0843	0.8639	0.9456	0.3656	0.8468
-2	0.0346	0.8885	0.9276	0.5129	0.7812
0	0.0199	0.3527	0.6790	0.9686	0.3048
1	0.9043	0.148	0.1654	0.4158	0.4612
2	0.0298	0.5646	0.2569	0.8167	0.3393
3	0.0468	0.4831	0.4435	0.7474	0.1636
4	0.1849	0.0032	0.6709	0.8037	0.3468

eTable 17.: SDR–TMLE Modelling of Effect Doubling Cannabis Exposure on ASDR by Ethnicity (from emmeans)

Race	N	Δ_Log (C.I.)	Δ_RR (C.I.)	Δ% (C.I.)
Hispanic	321	0.47 (0.19, 0.76)	1.60 (1.21, 2.13)	60.18 (20.56, 112.82)
NHWhite	331	0.32 (0.05, 0.58)	1.37 (1.05, 1.79)	37.35 (5.40, 79.98)
NHBlack	322	0.31 (0.02, 0.61)	1.37 (1.02, 1.84)	36.91 (2.11, 83.56)
Overall	1551	0.27 (-0.02, 0.56)	1.31 (0.98, 1.74)	30.58 (-2.14, 74.24)
NHAIAN	259	0.25 (NA, NA)	1.28 (NA, NA)	28.07 (NA, NA)
NHAsPI	318	0.08 (-1.06, 1.21)	1.08 (0.35, 3.36)	8.14 (-65.20, 236.03)

N = 1551
N_States = 42; NHAIAN uses only 39 states
Adjusted for: Cannabis, Income, Ascertainment, Legal Status, Year

eTable 18.: Sensitivity Testing: SDR–TMLE Modelling of Effect of Doubling Cannabis Exposure on ASDR

Model	N	N_States	Naïve State	Doubling State	Δ_Log (C.I.)	Δ_RR (C.I.)	Δ% (C.I.)
Model A	1551	42	3.86 (3.56, 4.17)	4.05 (3.77, 4.32)	0.18 (0.17, 0.20)	1.20 (1.18, 1.22)	20.01 (18.39, 21.65)
Model B	843	22	3.81 (3.34, 4.28)	4.00 (3.59, 4.42)	0.19 (0.17, 0.21)	1.21 (1.19, 1.23)	21.10 (18.98, 23.26)
Model C	1203	31	3.82 (3.56, 4.07)	3.95 (3.72, 4.18)	0.13 (0.12, 0.14)	1.14 (1.13, 1.15)	13.80 (12.50, 15.11)
Model D	1551	42	3.86 (3.56, 4.17)	4.10 (3.81, 4.39)	0.23 (0.21, 0.24)	1.26 (1.24, 1.28)	25.73 (23.84, 27.65)

A) Main SDR–TMLE (adjusted; doubling)
B) Legalization-change states only
C) Exclude minimal within-state cannabis change
D) TMLE without Status in covariate list

Covariate list includes: income, cannabis, ethnicity, case ascertainment, cannabis legal status, State and year.

eTable 19.: Generalized additive regression, Introductory models.

Parameters					Model					
**Term**	**edf**	**ref.df**	**Chi Squared**	**P-Value**	**AIC**	**BIC**	**DF**	**LogLik**	**Deviance**	**Adj.R.Squared**
Ethnicity										
ASD ~ Race										
Ethnicity	4.73	5	73.90	0.00E+00	8586	8617	5.73	-4287	3973	-2.06
Ethnic Cannabis										
ASD ~ eCannabis										
Ethn.Cannabis	8.56	8.94	615.00	0.00E+00	8040	8092	9.56	-4010	3419	-0.95
Additive model										
ASD ~ Race + eCannabis										
Ethnicity	4.96	5	108.00	0.00E+00	7942	8020	14.2	-3957	3312	-0.629
Ethn.Cannabis	8.27	8.85	643.00	0.00E+00						
Interaction - Direct										
ASD ~ Race * eCannabis										
Ethnicity	0.0024	5	0.0003	0.0063	7852	8026	31.5	-3895	3188	-0.557
Ethn.Cannabis	6.09	6.69	77.10	1.33E-06						
Ethn.Cannabis: Race	24.4	28	294.00	0.00E+00						
Interaction - as a Factor										
ASD ~ Race * eCannabis										
Ethnicity	2.17	5	9.08	0.0042	7872	8014	25.7	-3910	3219	-0.497
Ethn.Cannabis	8.05	8.64	106.00	0.00E+00						
Ethn.Cannabis: RaceNHAsPI	1.9	1.96	30.10	7.93E-07						
Ethn.Cannabis: RaceHispanic	4	4	50.20	0.00E+00						

eTable 20.: Modelling of ASD Rates in Nevada by ethnic cannabis exposure as mixed effects, survey regression, and polynomial quintic and quadratic functions.

Table Note: A quadratic function was used as only the first two levels of the quintic function were statistically significant.

Parameter			Model	
**Model & Terms**	**Estimate (C.I.)**	**P-Value**		
Mixed Effects Model				
ASDRt ~ eCigarettes + eCannabis + eBing.Alcohol + eAnalgesics + eCocaine + MHY + Race, Year, Random				
eBng.Alcohol	-2.12 (-4.05, -0.19)	0.0392	AIC	-24.2
eCannabis	0.51 (0.12, 0.9)	0.0162	BIC	-5.91
eAnalgesics	-8.44 (-16.12, -0.76)	0.0390	LogLik	23.1
Race.NHBlack	0.33 (0.07, 0.59)	0.0162	S.D.	0.0628
Survey GLM				
ASDRt ~ eCigarettes + eCannabis + eBing.Alcohol + eAnalgesics + eCocaine + MHY + Race				
eCannabis	11 (8.04, 13.96)	6.79E-06	AIC	36.4
eAnalgesics	-56.4 (-70.41, -42.39)	1.94E-14	BIC	35.3
Race.NHAsPI	-1.44 (-2.61, -0.27)	1.36E-06	deviance	4.36
Race.NHWhite	-0.27 (-0.6, 0.06)	1.08E-10		
Race.NHBlack	-0.77 (-1.24, -0.3)	1.81E-06		
Race.Hispanic	-0.43 (-0.92, 0.06)	0.0001		
Race.Total	-0.41 (-0.9, 0.09)	4.55E-08		
Polynomial, Quintic				
ASDRt ~ eCigarettes + eCannabis + eBing.Alcohol + eAnalgesics + eCocaine + MHY + Race + Period ^5				
eCannabis	3598 (558.04, 6637.96)	0.0262	AIC	493
Race.NHAIAN	-57 (-92.48, -21.52)	0.0033	BIC	517
Race.NHAsPI	39.4 (3.92, 74.88)	0.0360	LogLik	-233
Race.NHBlack	85.3 (49.82, 120.78)	3.53E-05	Adj.R.Squared	0.974
poly(Period, 5)1	719 (256.44, 1181.56)	0.0044	S.D.	36.1
poly(Period, 5)2	343 (117.6, 568.4)	0.0049	Model P-Value	2.98E-27
Polynomial, Quadratic				
ASDRt ~ eCigarettes + eCannabis + eBing.Alcohol + eAnalgesics + eCocaine + MHY + Race + Period ^2				
eCannabis	0.92 (0.74, 1.1)	2.48E-12	AIC	-60.4
eAnalgesics	-17.8 (-25.52, -10.08)	5.71E-05	BIC	-41.7
Race.NHAsPI	1.07 (0.48, 1.66)	0.0010	LogLik	40.2
Race.NHWhite	0.26 (0.09, 0.43)	0.0063	Adj.R.Squared	0.979
Race.Hispanic	0.35 (0.09, 0.62)	0.0132	S.D.	0.116
Race.Total	0.31 (0.07, 0.55)	0.0139	Model P-Value	6.09E-32
Period	0.16 (0.14, 0.18)	6.15E-16		

eTable 21.: Historical and predicted ASD rates for each of the exponential, quintic and supra-exponential models.

Year	Period	Observed	Quintic	Exponential	SupraExponential
2006	1	67.1	64.93	115.24	48.96
2007	2	69.1	77.58	115.68	56.77
2009	3	117.7	109.97	116.87	69.26
2010	4	165.0	155.94	120.13	89.29
2011	5	173.0	190.77	128.97	122.16
2013	6	245.7	228.90	218.31	277.44
2014	7	367.9	380.27	395.86	463.82
2018	8	884.0	881.16	878.48	834.54
2022	9	-	2,097.95	2,190.37	1,621.07
2026	10	-	4,571.99	5,756.48	3,409.45
2030	11	-	9,051.89	15,450.16	7,786.12
2034	12	-	16,525.81	41,800.32	19,359.42

SUPPLEMENTARY FIGURES

List of eFigures

eFigure 1.: ASD Rates by Ethnicity and State, A-M.

eFigure 2.: ASD Rates by Ethnicity and State, M-W.

eFigure 3.: Notched boxplots of ASD rates by Ethnicity

eFigure 4.: Bar Graph with confidence intervals of ASD rates by Ethnicity

eFigure 5.: Model comparisons from eTable 6. Prepared with the performance library in RStudio.

eFigure 6.: Figure 2.: Ethnic and Cannabinoid Metrics of ASD Rates. (A) Log (ASD rate) over time by dichotomized ethnicity; (B) ASD Rate by Ethnic Cannabis x Δ9THC exposure; (C) ASD Rate by Ethnic Cannabis x Δ9THC exposure by dichotomized ethnicity, loess curves fitted, (D) Log (ASD Rate) by Ethnic Cannabis x Δ9THC exposure by dichotomized ethnicity, regression lines fitted.

eFigure 7.: Bar Graph of Mixed Effects Multivariate Model with effect sizes and confidence intervals plotted from Model 4 eTable 5.

eFigure 8.: Predicted Values from Model 4 eTable 5.

eFigure 9.: Difference in Difference Chart by Ethnicity and Overall. Changes in ASDR listed as percent change.

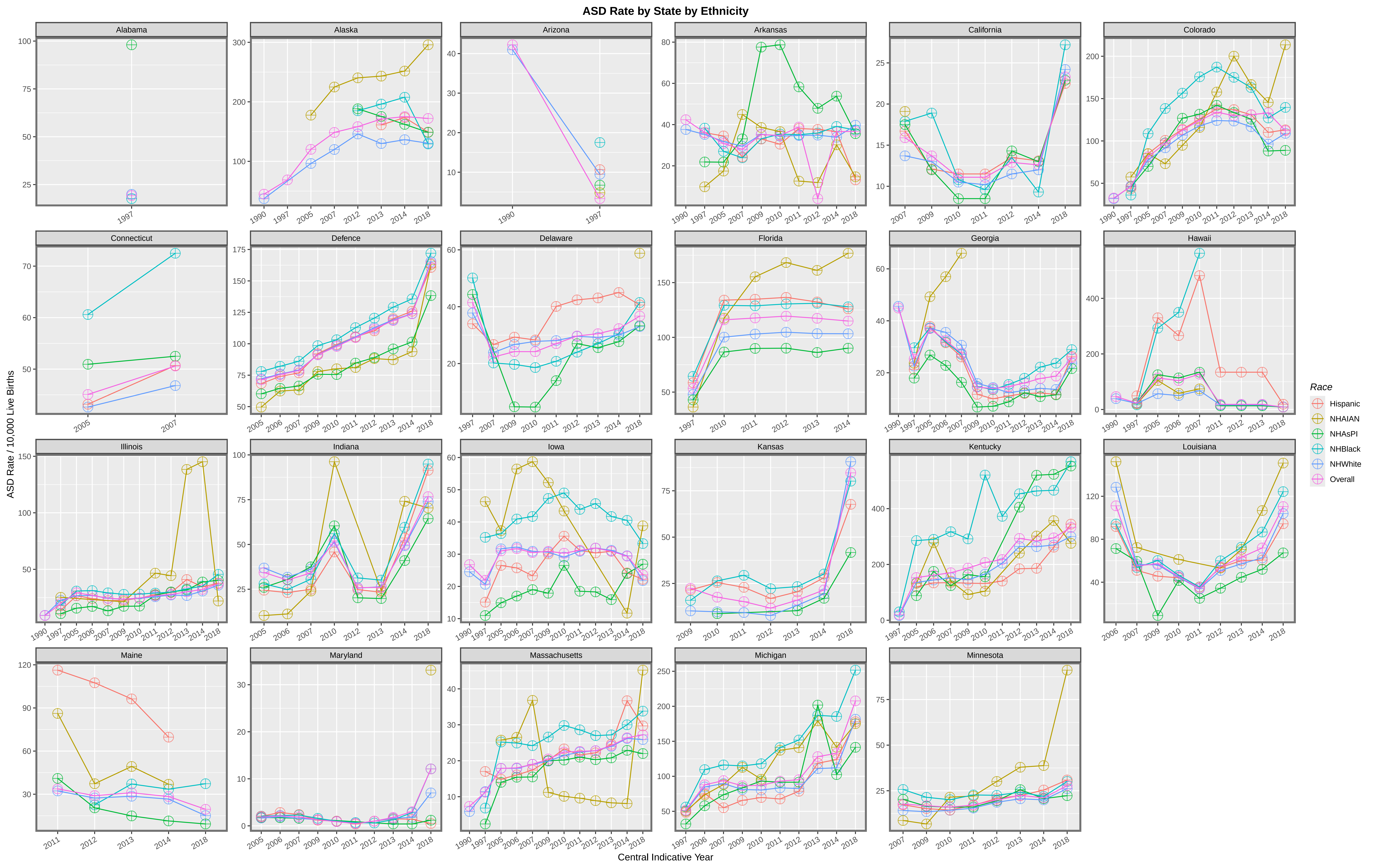
ASD Rate by State by Ethnicity
Alabama
Alaska
Arizona
Arkansas
California
Colorado
Connecticut
Defence
Delaware
Florida
Georgia
Hawaii
Illinois
Indiana
Iowa
Kansas
Kentucky
Louisiana
Maine
Maryland
Massachusetts
Michigan
Minnesota
ASD Rate / 10,000 Live Births
Central Indicative Year
Race
Hispanic
NHAIAN
NHAsPI
NHBlack
NHWhite
Overall

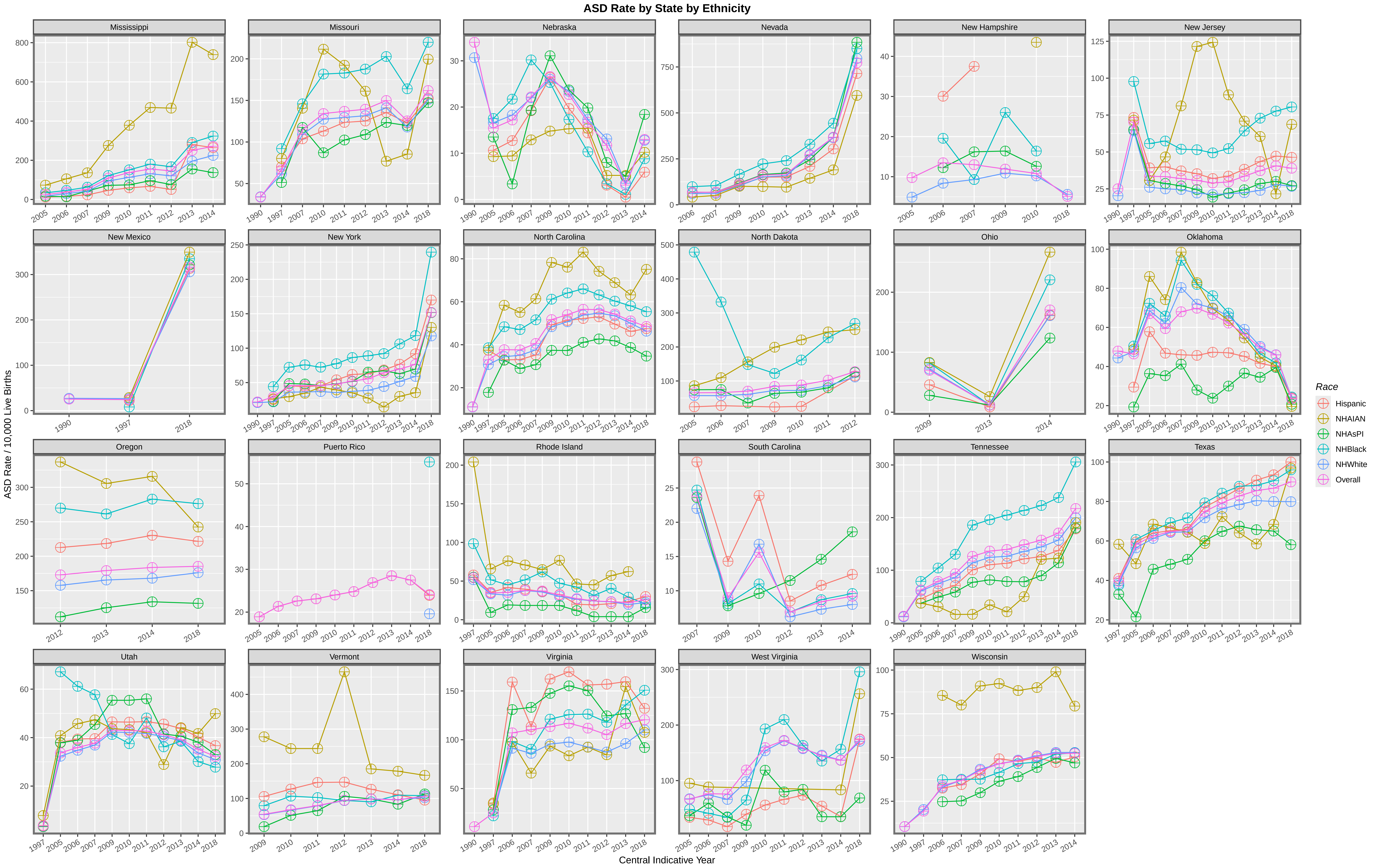

ASD Rate by State by Ethnicity
Mississippi
Missouri
Nebraska
Nevada
New Hampshire
New Jersey
New Mexico
New York
North Carolina
North Dakota
Ohio
Oklahoma
Oregon
Puerto Rico
Rhode Island
South Carolina
Tennessee
Texas
Utah
Vermont
Virginia
West Virginia
Wisconsin
ASD Rate / 10,000 Live Births
Central Indicative Year
Race
Hispanic
NHAIAN
NHAsPI
NHBlack
NHWhite
Overall

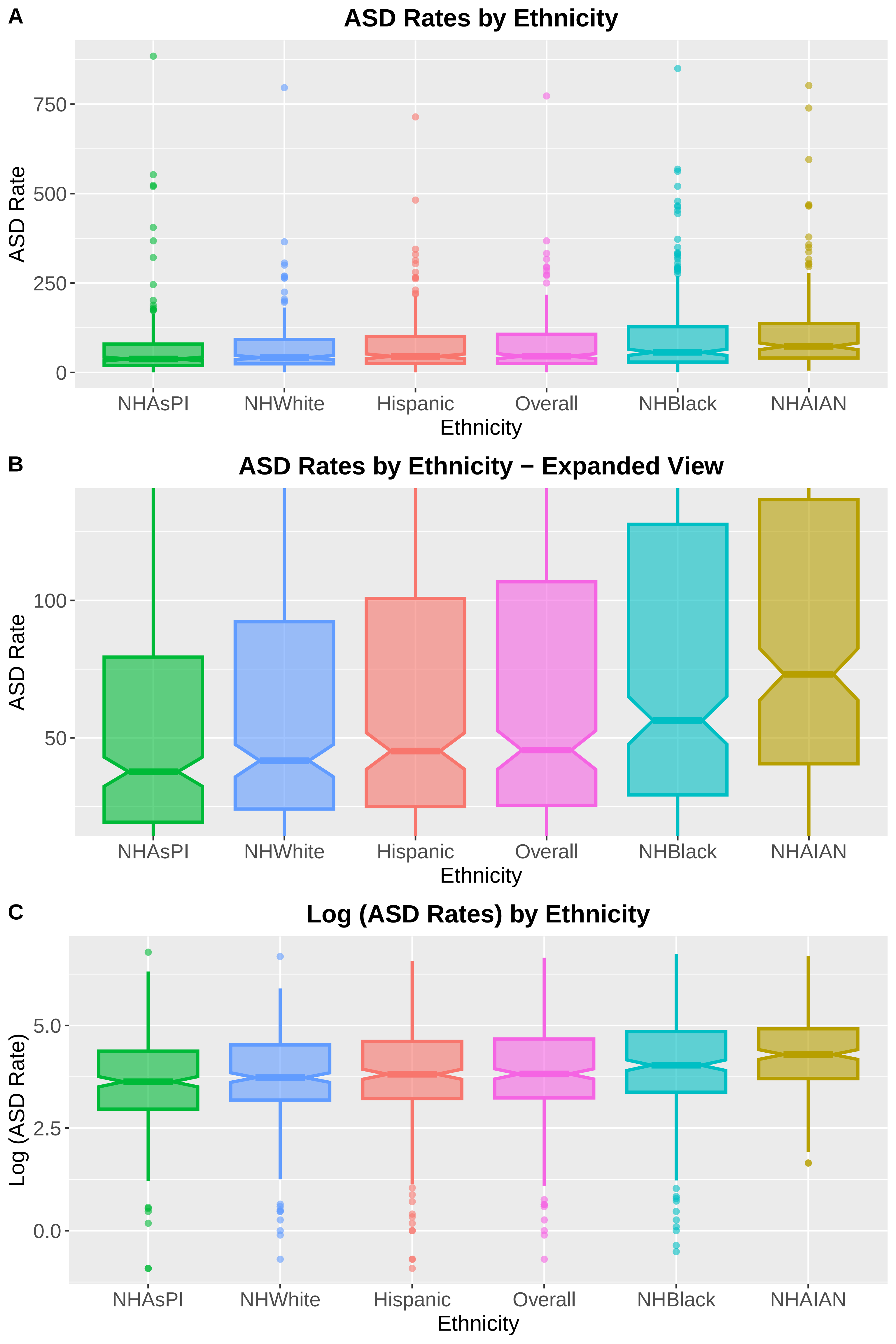
A
ASD Rates by Ethnicity
ASD Rate
0
250
500
750
NHAsPI
NHWhite
Hispanic
Overall
NHBlack
NHAIAN
Ethnicity
B
ASD Rates by Ethnicity − Expanded View
ASD Rate
50
100
NHAsPI
NHWhite
Hispanic
Overall
NHBlack
NHAIAN
Ethnicity
C
Log (ASD Rates) by Ethnicity
Log (ASD Rate)
0.0
2.5
5.0
NHAsPI
NHWhite
Hispanic
Overall
NHBlack
NHAIAN
Ethnicity

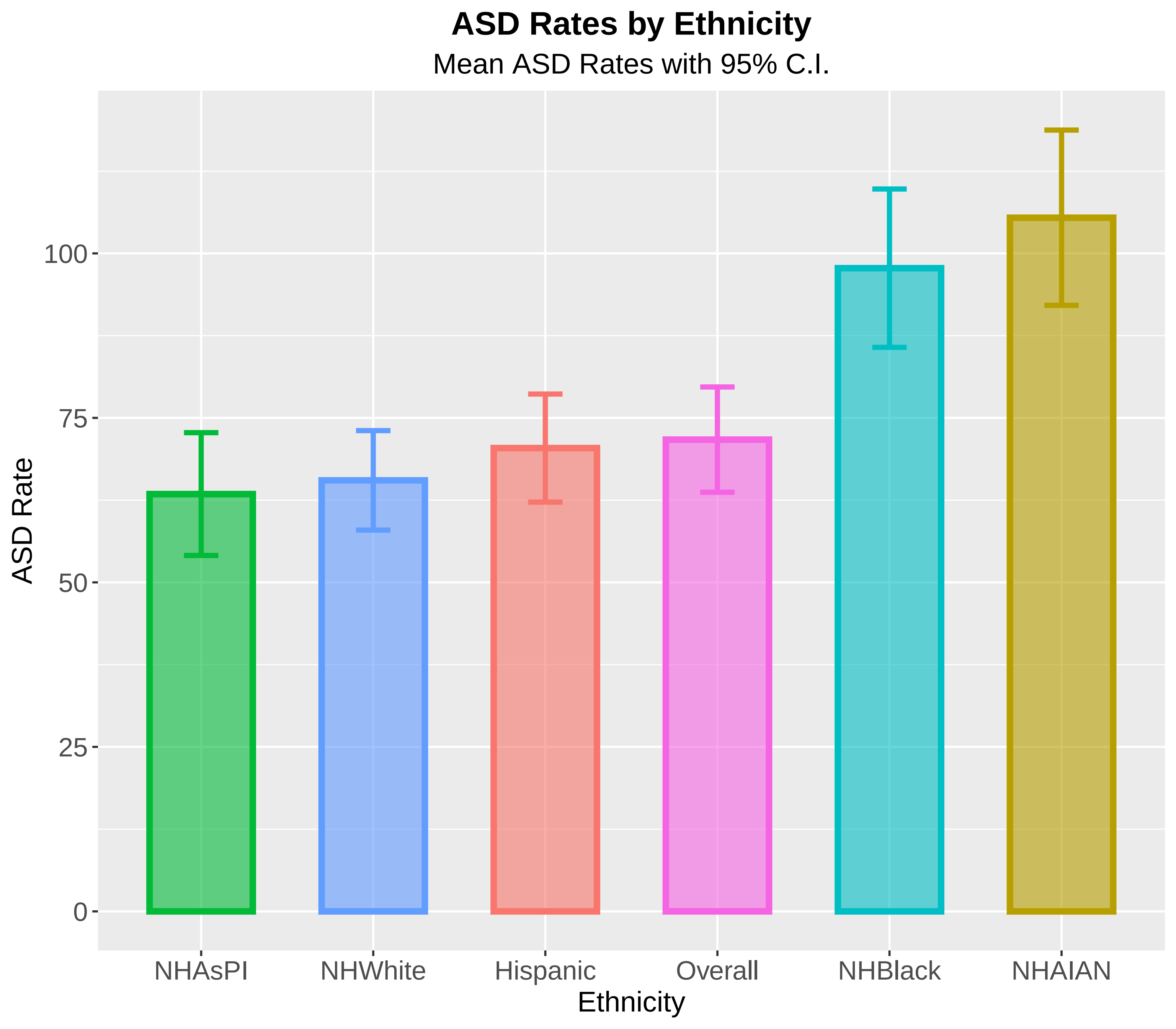
ASD Rates by Ethnicity
Mean ASD Rates with 95% C.I.
ASD Rate
100
75
50
25
0
NHAsPI
NHWhite
Hispanic
Overall
NHBlack
NHAIAN
Ethnicity

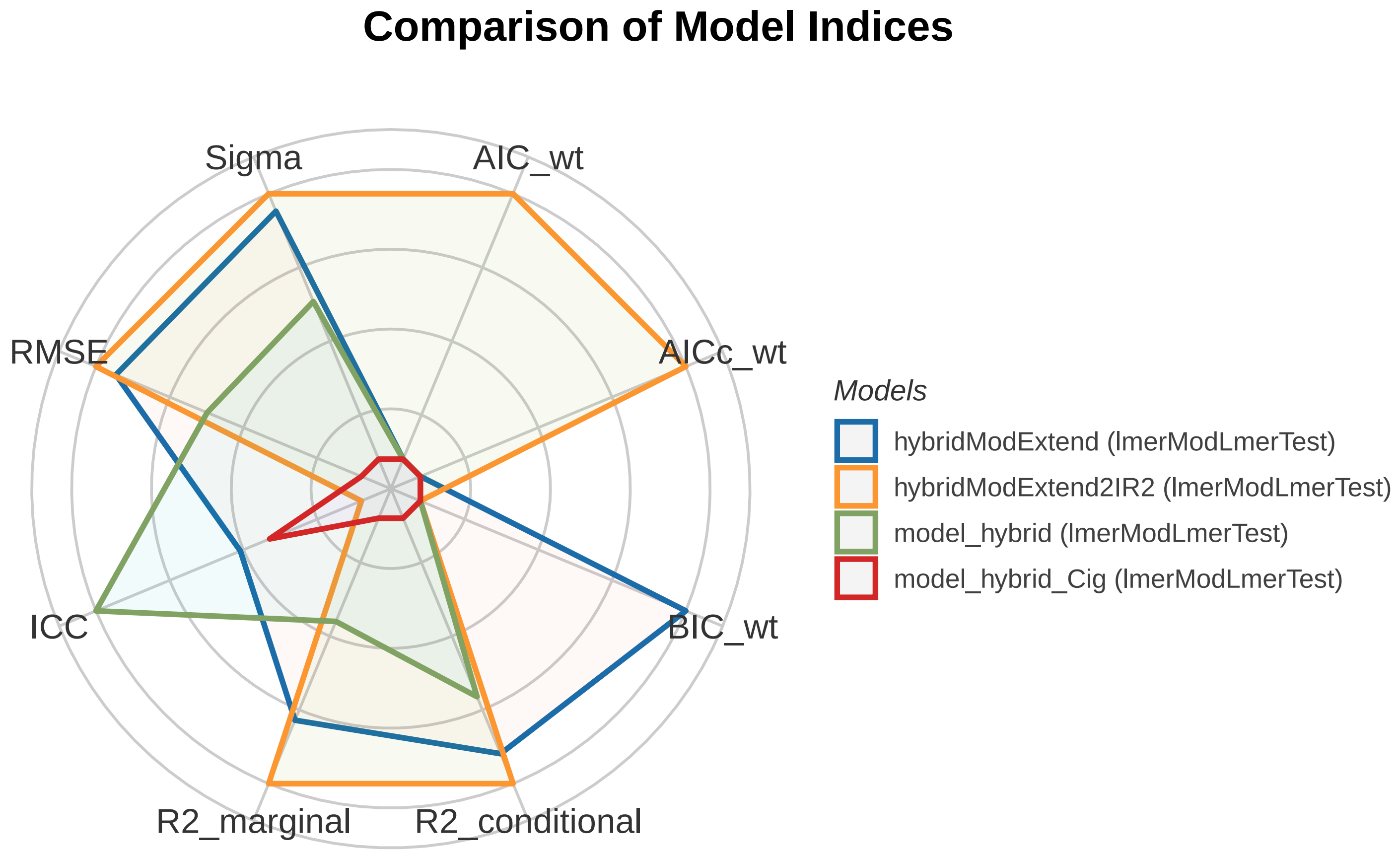
Comparison of Model Indices
Sigma
AIC_wt
AICc_wt
BIC_wt
R2_conditional
R2_marginal
ICC
RMSE
Models
hybridModExtend (lmerModLmerTest)
hybridModExtend2IR2 (lmerModLmerTest)
model_hybrid (lmerModLmerTest)
model_hybrid_Cig (lmerModLmerTest)

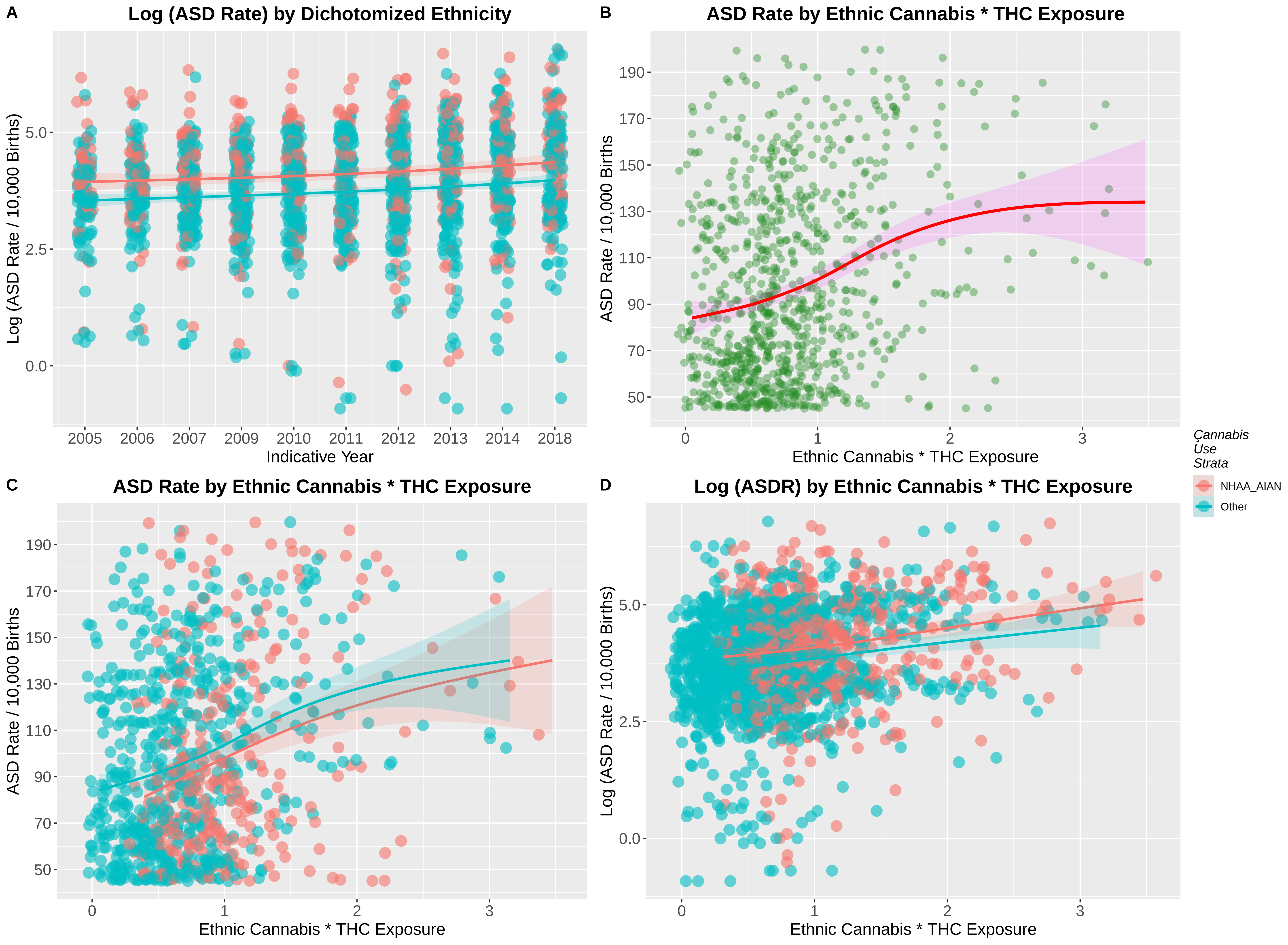

A
Log (ASD Rate) by Dichotomized Ethnicity
Log (ASD Rate / 10,000 Births)
5.0
2.5
0.0
2005
2006
2007
2009
2010
2011
2012
2013
2014
2018
Indicative Year
B
ASD Rate by Ethnic Cannabis * THC Exposure
ASD Rate / 10,000 Births
190
170
150
130
110
90
70
50
0
1
2
3
Ethnic Cannabis * THC Exposure
C
ASD Rate by Ethnic Cannabis * THC Exposure
ASD Rate / 10,000 Births
Ethnic Cannabis * THC Exposure
D
Log (ASDR) by Ethnic Cannabis * THC Exposure
Log (ASD Rate / 10,000 Births)
Ethnic Cannabis * THC Exposure
Cannabis
Use
Strata
NHAA_AIAN
Other

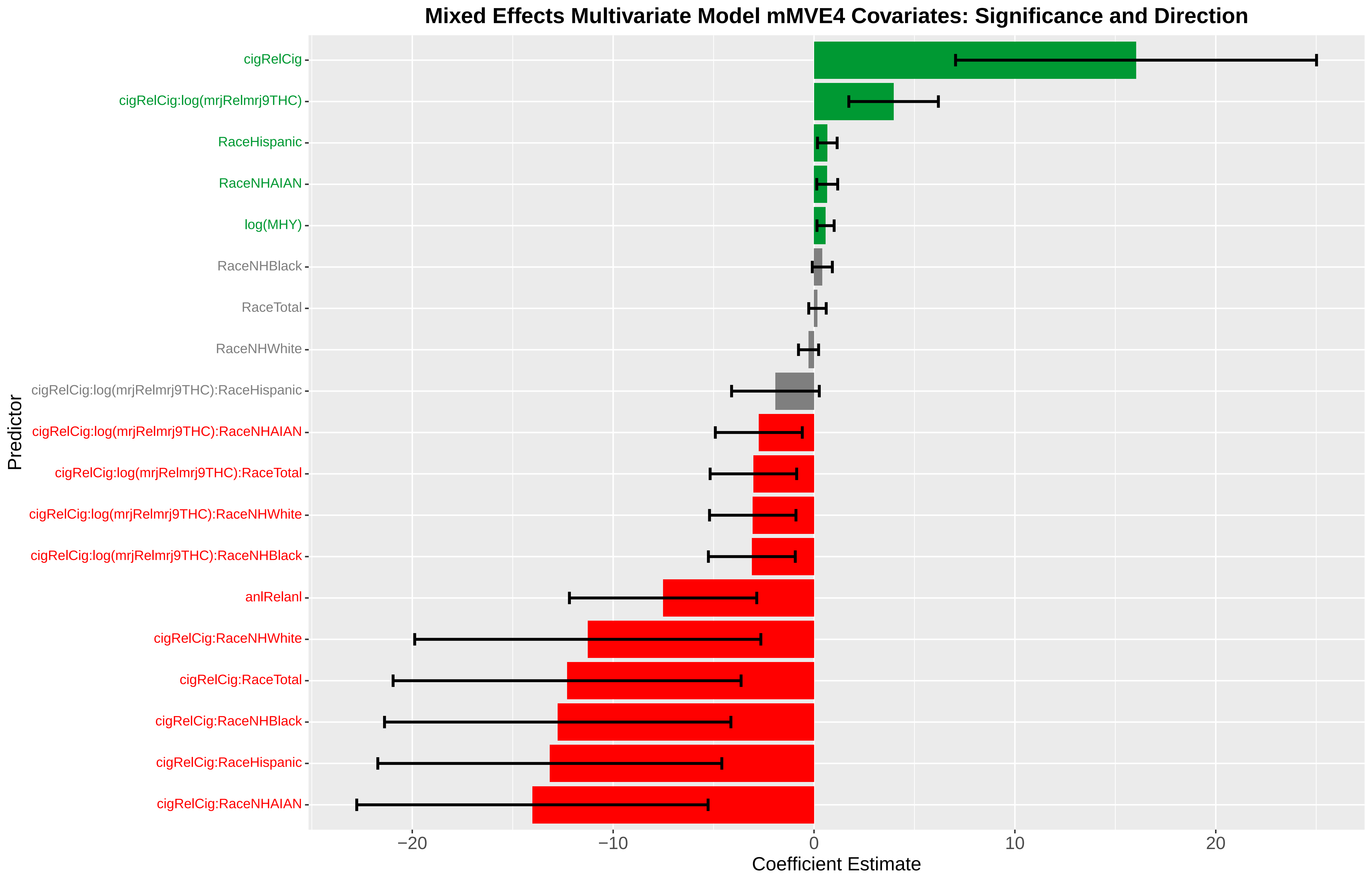

Mixed Effects Multivariate Model mMVE4 Covariates: Significance and Direction
cigRelCig
cigRelCig:log(mrjRelmrj9THC)
RaceHispanic
RaceNHAIAN
log(MHY)
RaceNHBlack
RaceTotal
RaceNHWhite
cigRelCig:log(mrjRelmrj9THC):RaceHispanic
cigRelCig:log(mrjRelmrj9THC):RaceNHAIAN
cigRelCig:log(mrjRelmrj9THC):RaceTotal
cigRelCig:log(mrjRelmrj9THC):RaceNHWhite
cigRelCig:log(mrjRelmrj9THC):RaceNHBlack
anlRelanl
cigRelCig:RaceNHWhite
cigRelCig:RaceTotal
cigRelCig:RaceNHBlack
cigRelCig:RaceHispanic
cigRelCig:RaceNHAIAN
Predictor
−20
−10
0
10
20
Coefficient Estimate

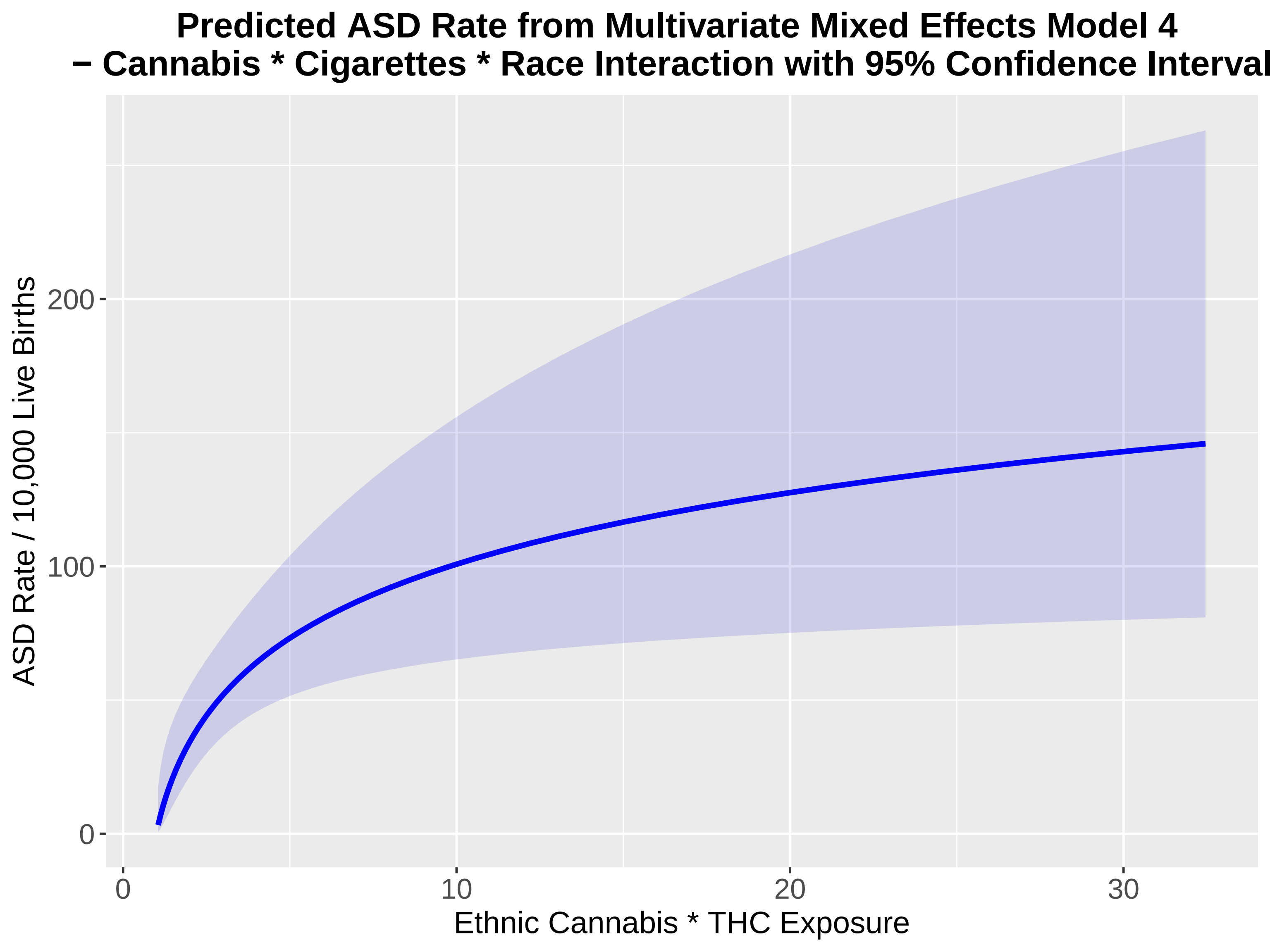

Predicted ASD Rate from Multivariate Mixed Effects Model 4
− Cannabis * Cigarettes * Race Interaction with 95% Confidence Interval
ASD Rate / 10,000 Live Births
200
100
0
0
10
20
30
Ethnic Cannabis * THC Exposure

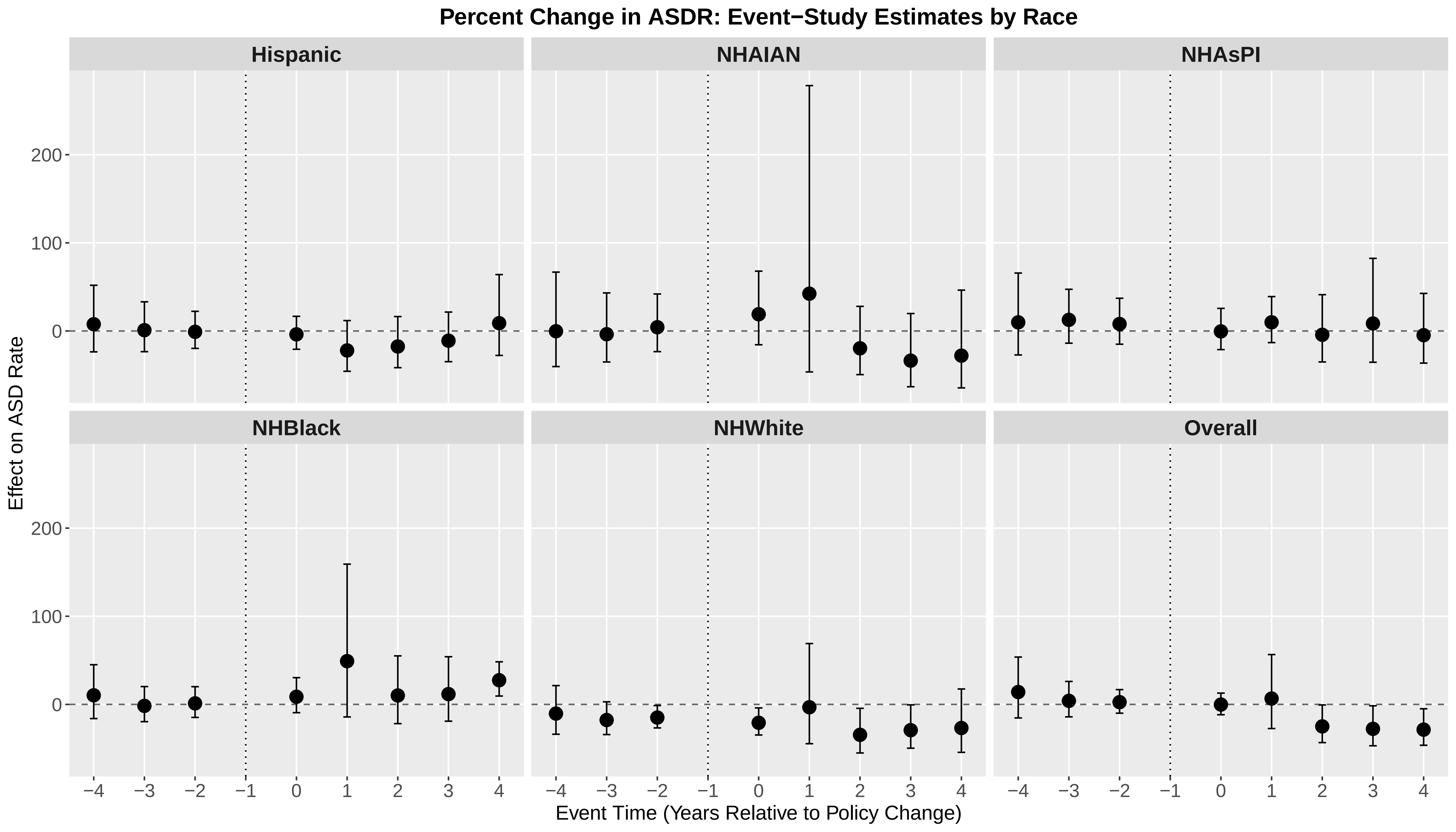
Percent Change in ASDR: Event−Study Estimates by Race
Hispanic
NHAIAN
NHAsPI
NHBlack
NHWhite
Overall
Effect on ASD Rate
200
100
0
−4
−3
−2
−1
0
1
2
3
4
Event Time (Years Relative to Policy Change)
